# Supplementary material for: NRF2 supports non-small cell lung cancer growth independently of CBP/p300-enhanced glutathione synthesis
Source: EMBO Rep. 2025 May 14;26(12):3106–37. doi: 10.1038/s44319-025-00463-z (PMC12187939; doi:10.1038/s44319-025-00463-z)
Supplement: Supplementary file 3 — Appendix [file 44319_2025_463_MOESM3_ESM.pdf]

Appendix

NRF2 supports non-small cell lung cancer growth independently of CBP/p300-enhanced glutathione synthesis

Conrad et al 2025

Genome browser snapshots of NRF2 signature genes including simulated H3K27ac Hi-ChIP and NRF2 knockdown PRO-seq

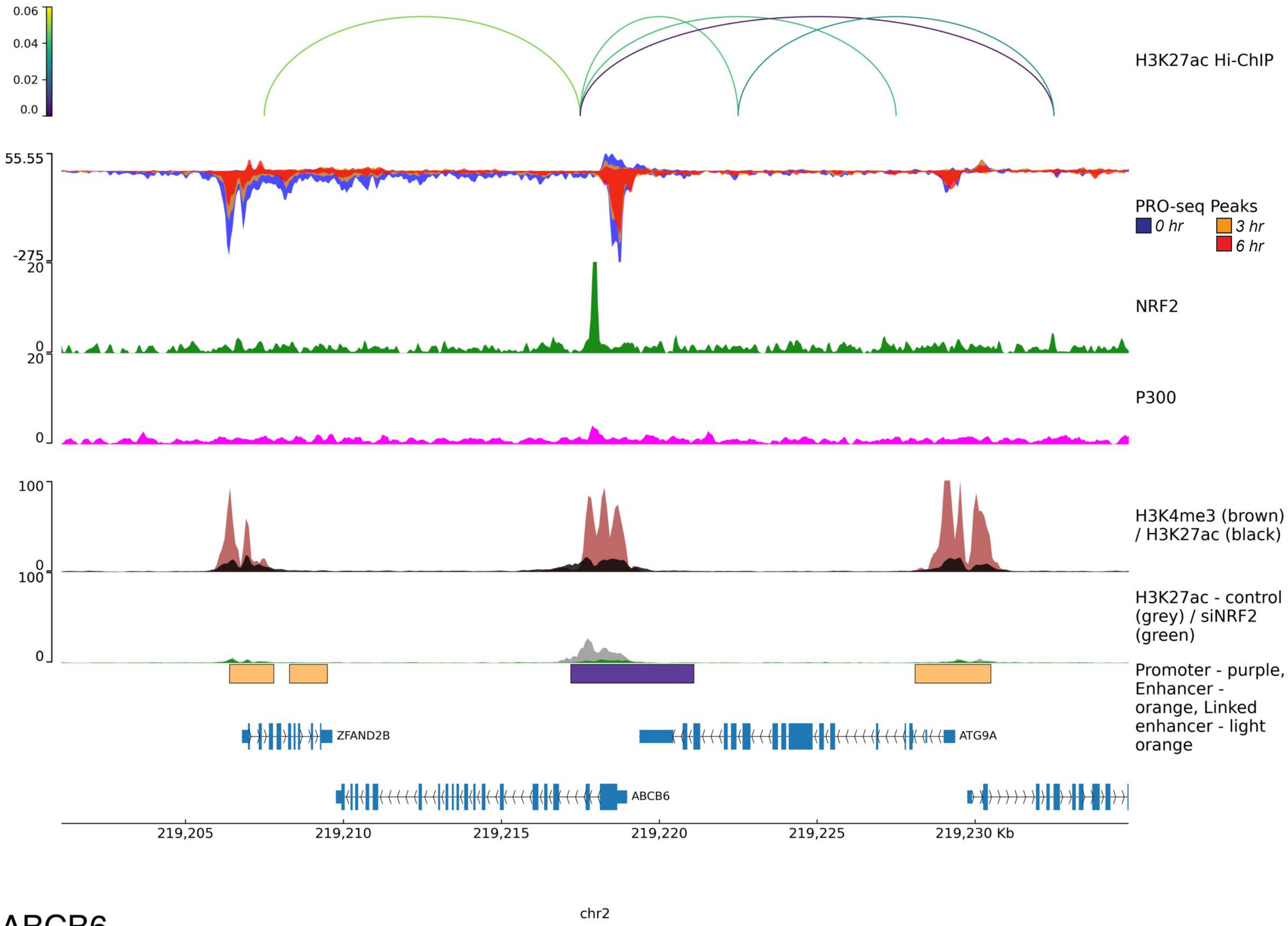

0.06
0.04
0.02
0.0
H3K27ac Hi-ChIP
55.55
-275
PRO-seq Peaks
0 hr
3 hr
6 hr
20
0
NRF2
20
0
P300
100
0
H3K4me3 (brown) / H3K27ac (black)
100
0
H3K27ac - control (grey) / siNRF2 (green)
Promoter - purple, Enhancer - orange, Linked enhancer - light orange
ZFAND2B
ATG9A
ABCB6
219,205
219,210
219,215
219,220
219,225
219,230 Kb
chr2

ABCB6

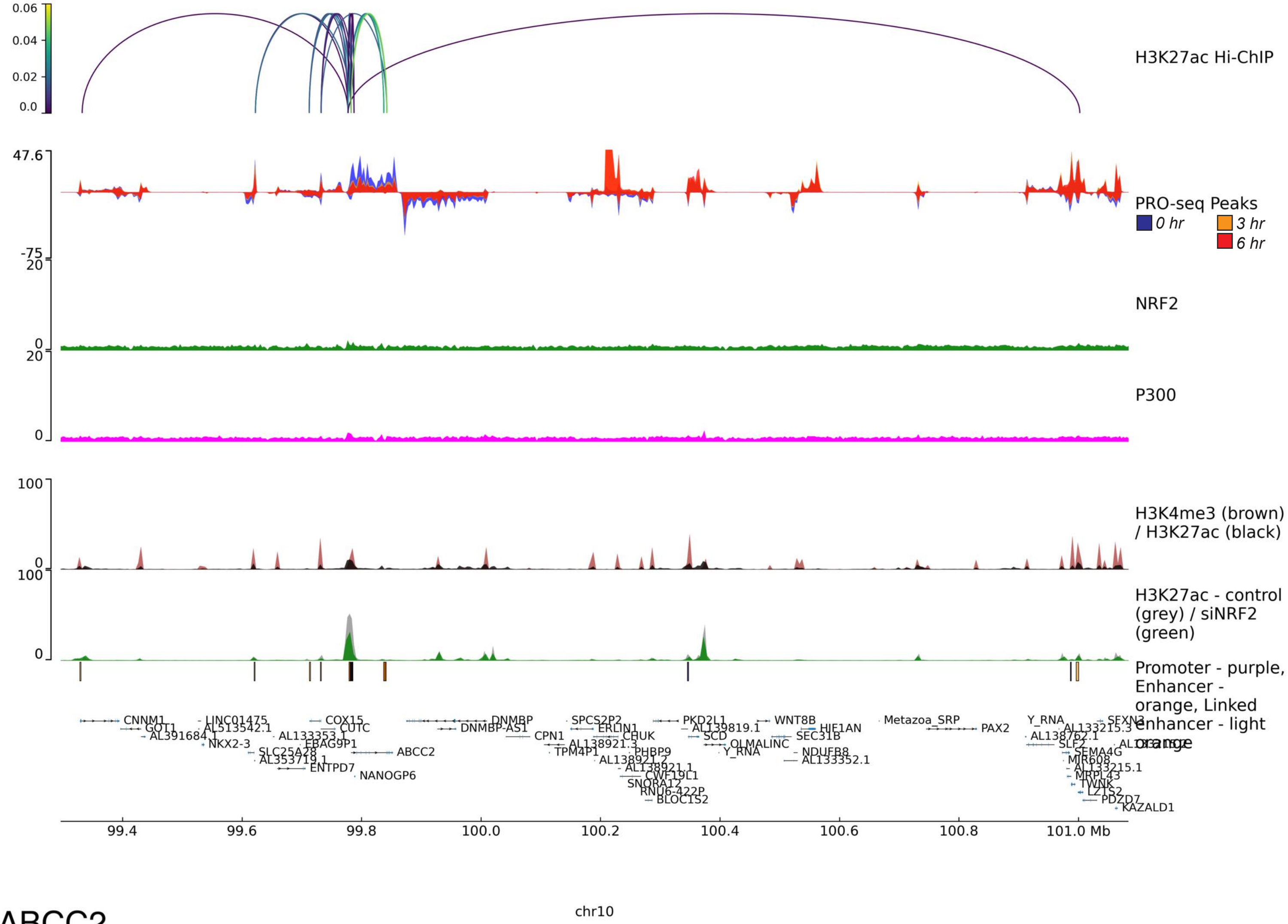
H3K27ac Hi-ChIP
0.06
0.04
0.02
0.0
47.6
-75
PRO-seq Peaks
0 hr
3 hr
6 hr
20
0
NRF2
20
0
P300
100
0
H3K4me3 (brown) / H3K27ac (black)
100
0
H3K27ac - control (grey) / siNRF2 (green)
Promoter - purple, Enhancer - orange, Linked enhancer - light orange
CNNM1
GOT1
AL391684.1
LINC01475
AL513542.1
NKX2-3
SLC25A28
AL353719.1
AL133353.1
ENTPD7
COX15
CUTC
EBAG9P1
ABCC2
NANOGP6
DNMBP
DNMBP-AS1
CPN1
SPCS2P2
ERLIN1
TPM4P1
CHUK
AL138921.3
AL138921.2
PHBP9
AL138921.1
CWF19L1
SNORA12
RNU6-422P
BLOC1S2
PKD2L1
AL139819.1
SCD
OLMALINC
Y_RNA
WNT8B
HIF1AN
SEC31B
NDUFB8
AL133352.1
Metazoa_SRP
PAX2
Y_RNA
AL133215.3
AL138762.1
SLF2
SEMA4G
MIR608
AL133215.1
MRPL43
TWNK
LZTS2
PDZD7
KAZALD1
SFXN3
99.4
99.6
99.8
100.0
100.2
100.4
100.6
100.8
101.0 Mb
chr10

ABCC2

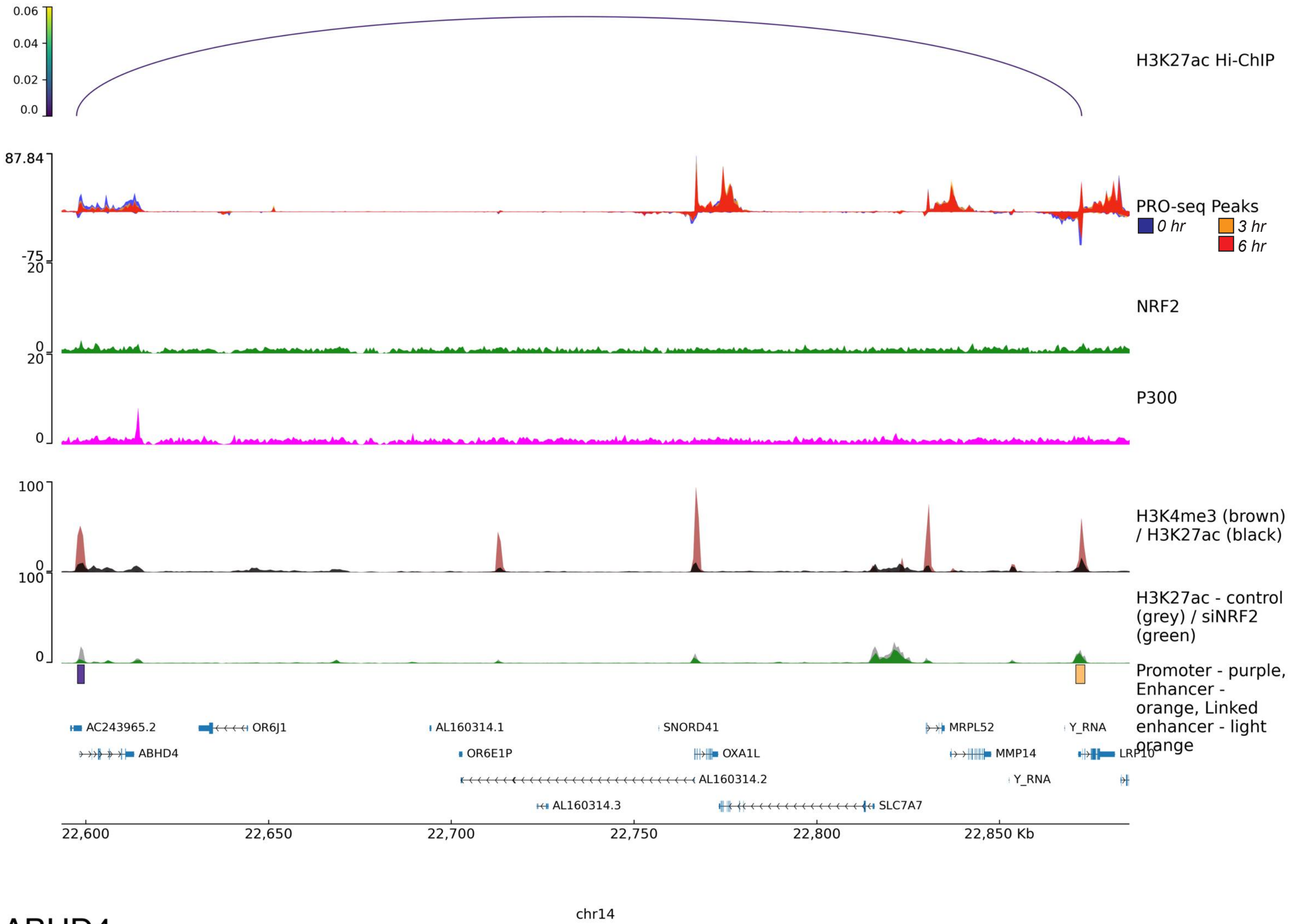
0.06
0.04
0.02
0.0
H3K27ac Hi-ChIP
87.84
-75
PRO-seq Peaks
0 hr
3 hr
6 hr
20
0
NRF2
20
0
P300
100
0
H3K4me3 (brown) / H3K27ac (black)
100
0
H3K27ac - control (grey) / siNRF2 (green)
Promoter - purple, Enhancer - orange, Linked enhancer - light orange
AC243965.2
OR6J1
AL160314.1
SNORD41
MRPL52
Y_RNA
ABHD4
OR6E1P
OXA1L
MMP14
LRP10
AL160314.2
Y_RNA
AL160314.3
SLC7A7
22,600
22,650
22,700
22,750
22,800
22,850 Kb
chr14

ABHD4

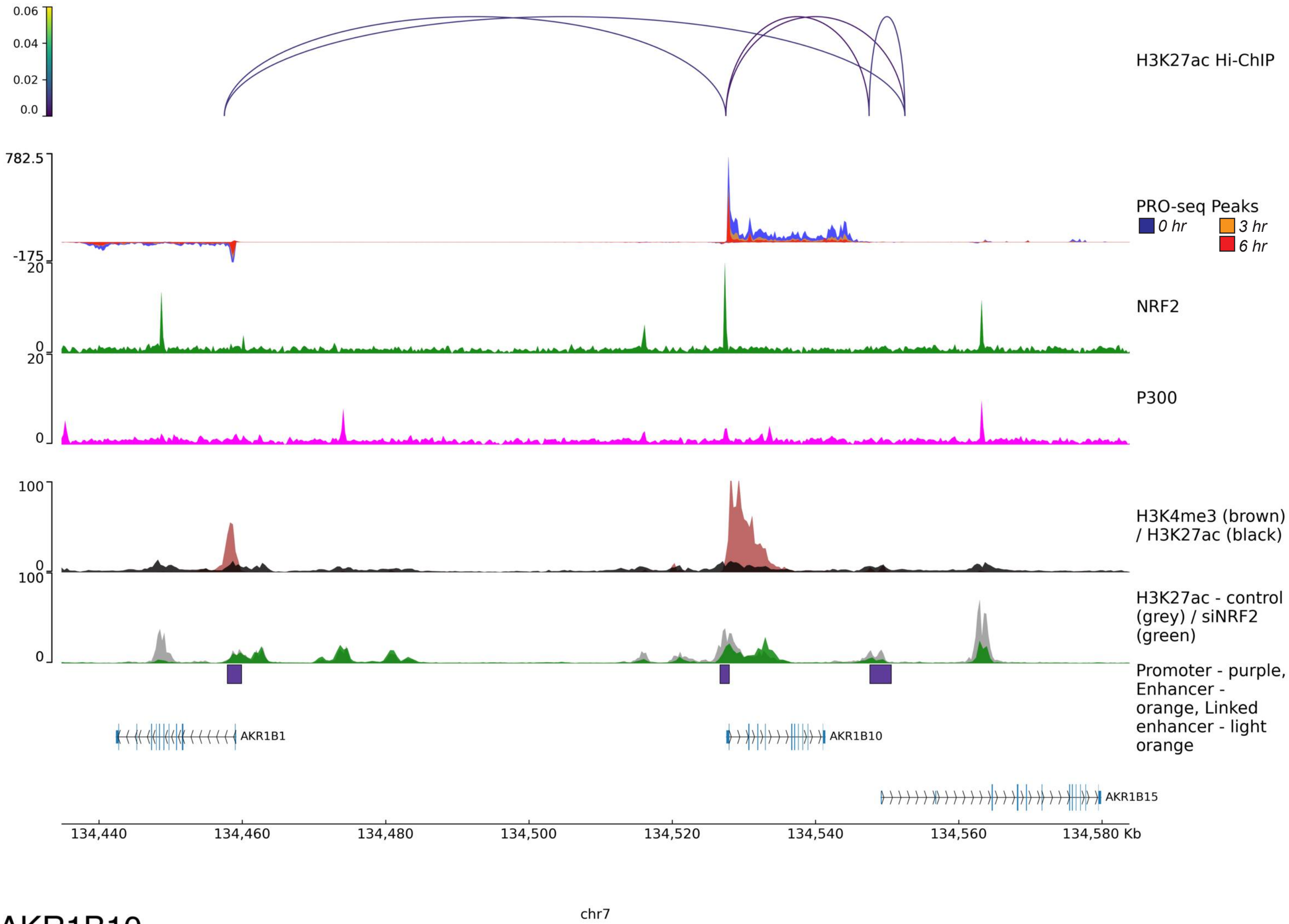
0.06
0.04
0.02
0.0
H3K27ac Hi-ChIP
782.5
-175
PRO-seq Peaks
0 hr
3 hr
6 hr
20
0
NRF2
20
0
P300
100
0
H3K4me3 (brown) / H3K27ac (black)
100
0
H3K27ac - control (grey) / siNRF2 (green)
Promoter - purple, Enhancer - orange, Linked enhancer - light orange
AKR1B1
AKR1B10
AKR1B15
134,440
134,460
134,480
134,500
134,520
134,540
134,560
134,580 Kb
chr7

AKR1B10

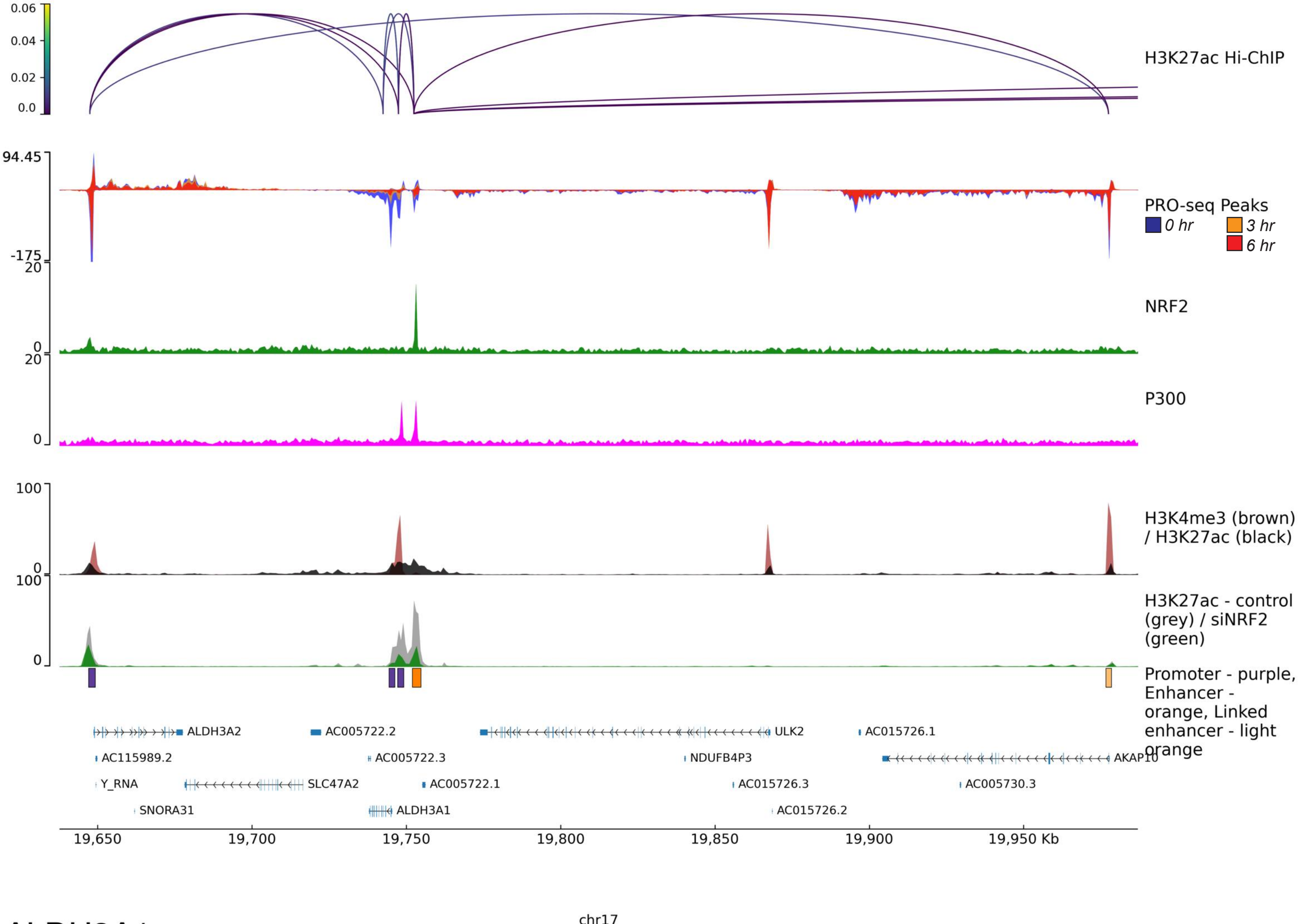

H3K27ac Hi-ChIP
PRO-seq Peaks
0 hr
3 hr
6 hr
NRF2
P300
H3K4me3 (brown) / H3K27ac (black)
H3K27ac - control (grey) / siNRF2 (green)
Promoter - purple, Enhancer - orange, Linked enhancer - light orange
ALDH3A2
AC005722.2
ULK2
AC015726.1
AC115989.2
AC005722.3
NDUFB4P3
AKAP10
Y_RNA
SLC47A2
AC005722.1
AC015726.3
AC005730.3
SNORA31
ALDH3A1
AC015726.2
19,650
19,700
19,750
19,800
19,850
19,900
19,950 Kb
chr17

ALDH3A1

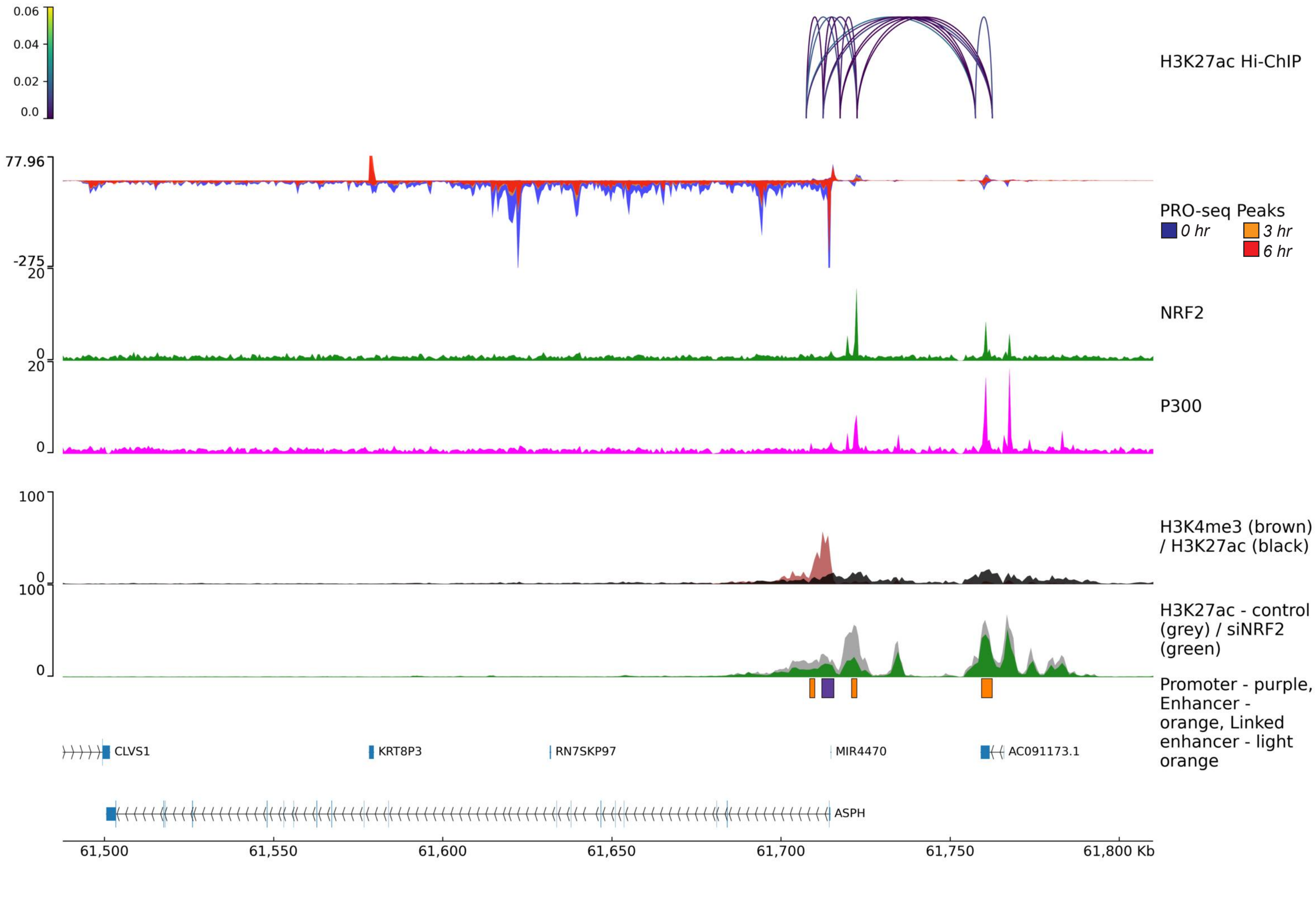

H3K27ac Hi-ChIP
PRO-seq Peaks
0 hr
3 hr
6 hr
NRF2
P300
H3K4me3 (brown) / H3K27ac (black)
H3K27ac - control (grey) / siNRF2 (green)
Promoter - purple, Enhancer - orange, Linked enhancer - light orange
CLVS1
KRT8P3
RN7SKP97
MIR4470
AC091173.1
ASPH
61,500
61,550
61,600
61,650
61,700
61,750
61,800 Kb
chr8

ASPH

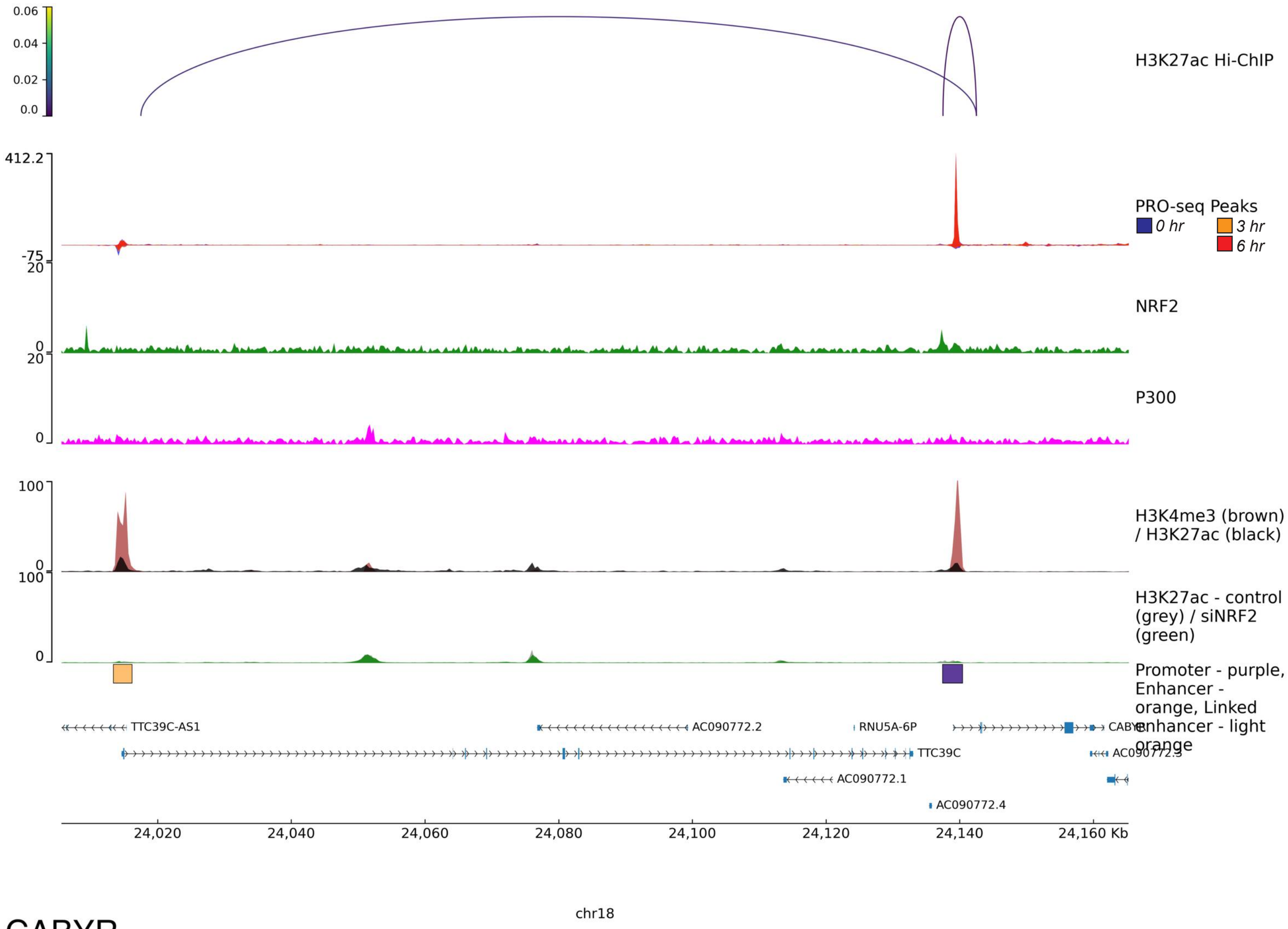
H3K27ac Hi-ChIP
PRO-seq Peaks
0 hr
3 hr
6 hr
NRF2
P300
H3K4me3 (brown) / H3K27ac (black)
H3K27ac - control (grey) / siNRF2 (green)
Promoter - purple, Enhancer - orange, Linked enhancer - light orange
TTC39C-AS1
AC090772.2
RNU5A-6P
CABYR
TTC39C
AC090772.3
AC090772.1
AC090772.4
24,020
24,040
24,060
24,080
24,100
24,120
24,140
24,160 Kb
chr18

CABYR

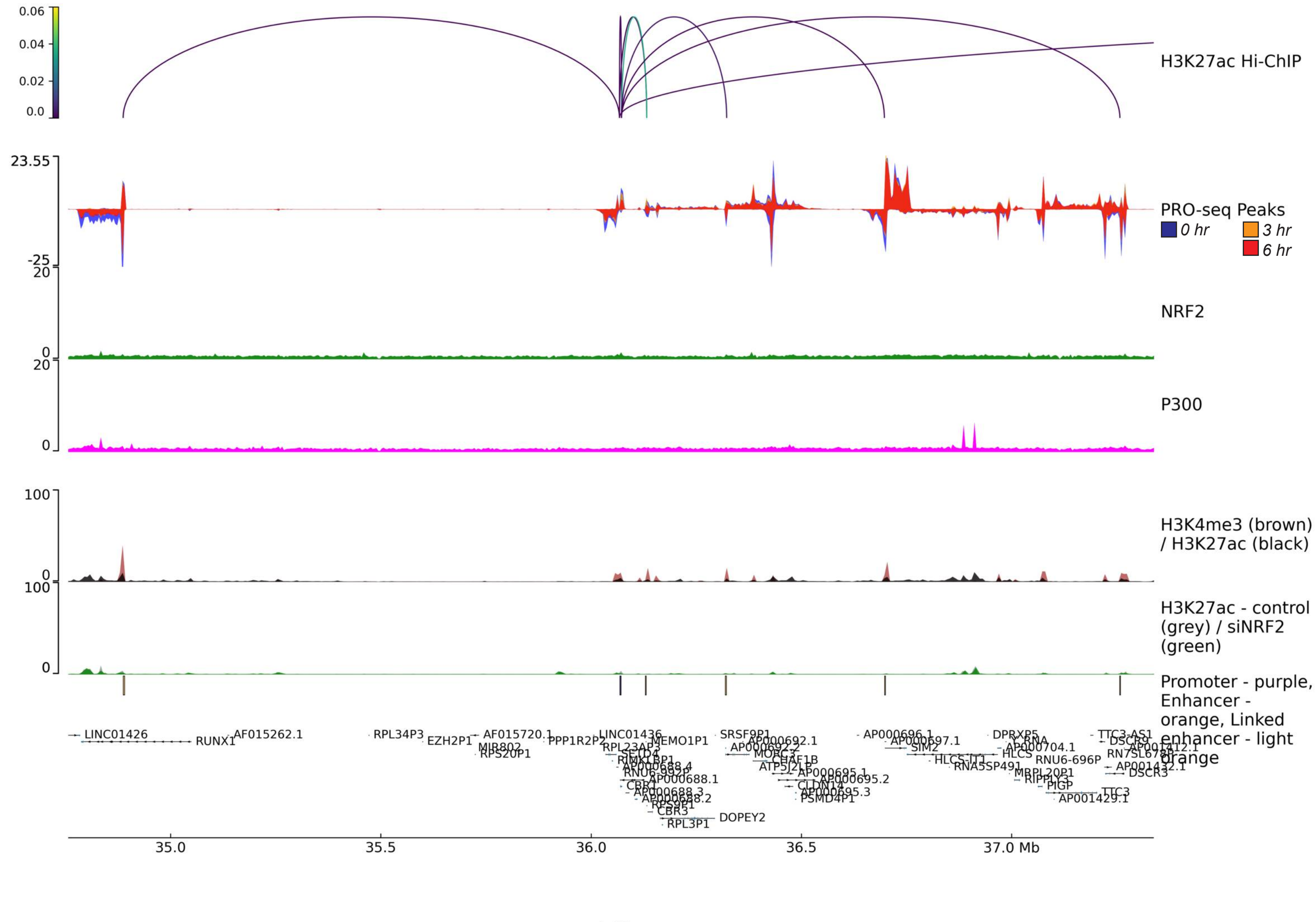

0.06
0.04
0.02
0.0
H3K27ac Hi-ChIP
23.55
-25
PRO-seq Peaks
0 hr
3 hr
6 hr
20
0
NRF2
20
0
P300
100
0
H3K4me3 (brown) / H3K27ac (black)
100
0
H3K27ac - control (grey) / siNRF2 (green)
Promoter - purple, Enhancer - orange, Linked enhancer - light orange
LINC01426
RUNX1
AF015262.1
RPL34P3
EZH2P1
AF015720.1
MIR802
RPS20P1
PPP1R2P2
LINC01436
MEMO1P1
SRSF9P1
AP000692.1
AP000696.1
AP000697.1
SIM2
DPRXP5
AP000704.1
HLCS
TTC3-AS1
DSCR9
CBR1
CBR3
DOPEY2
RPL3P1
CLDN14
PSMD4P1
HLCS-IT1
RNA5SP491
RNU6-696P
RN7SL678P
DSCR3
TTC3
AP001429.1
35.0
35.5
36.0
36.5
37.0 Mb
chr21

CBR1

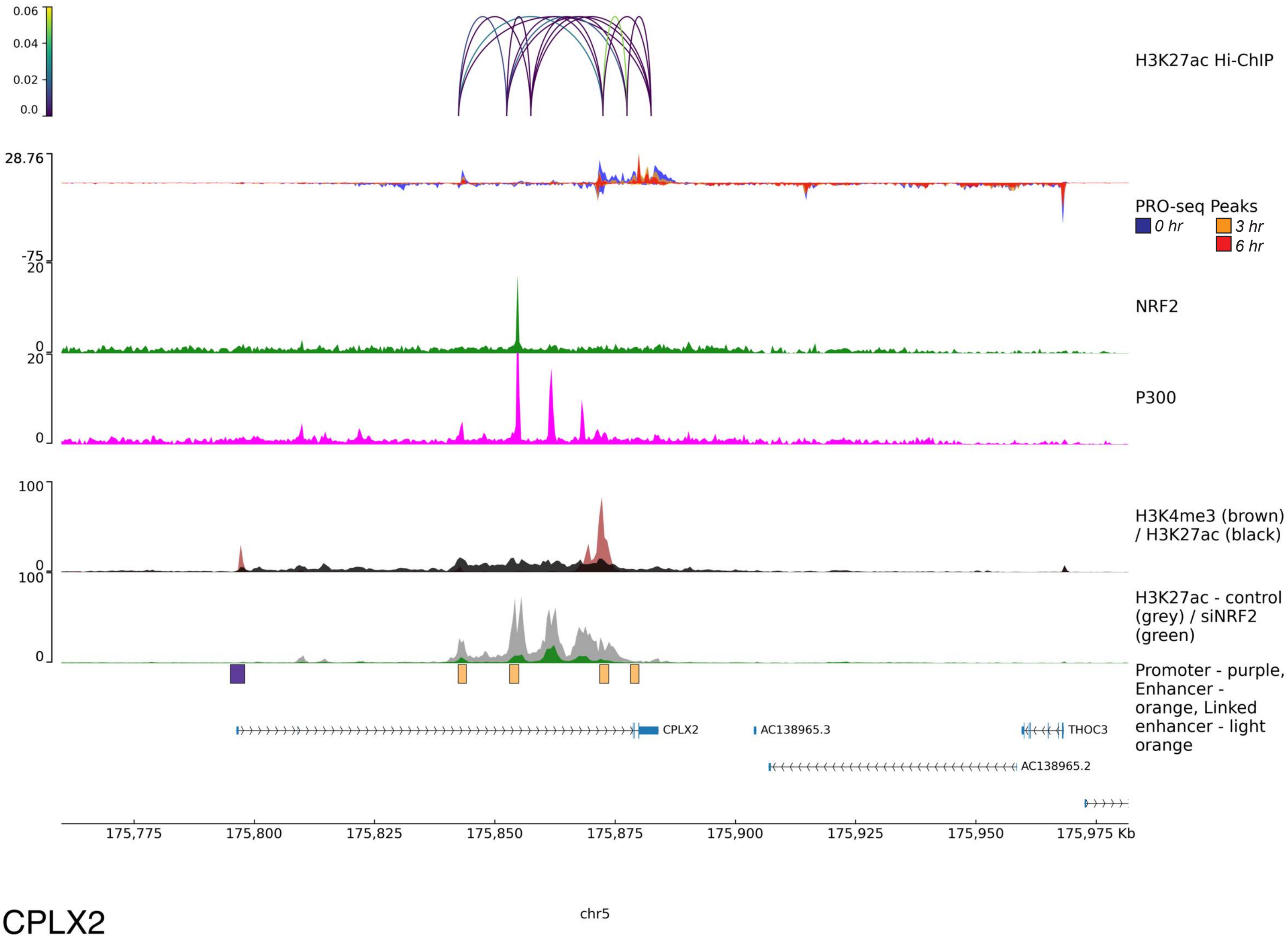

0.06
0.04
0.02
0.0
H3K27ac Hi-ChIP
28.76
-75
PRO-seq Peaks
0 hr
3 hr
6 hr
20
0
NRF2
20
0
P300
100
0
H3K4me3 (brown) / H3K27ac (black)
100
0
H3K27ac - control (grey) / siNRF2 (green)
Promoter - purple, Enhancer - orange, Linked enhancer - light orange
CPLX2
AC138965.3
THOC3
AC138965.2
175,775
175,800
175,825
175,850
175,875
175,900
175,925
175,950
175,975 Kb
chr5

CPLX2

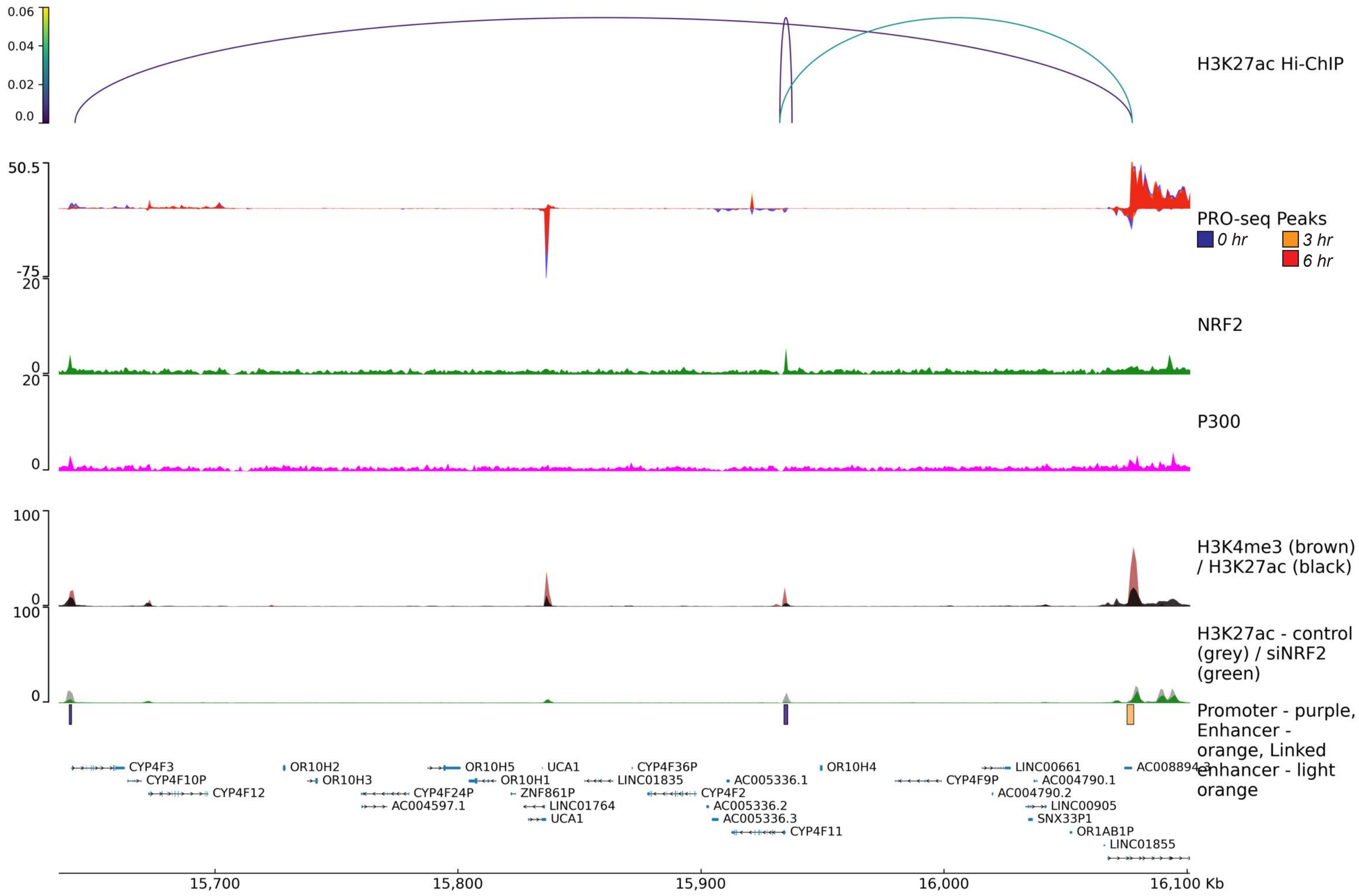

CYP4F3_CYP4F11

chr19

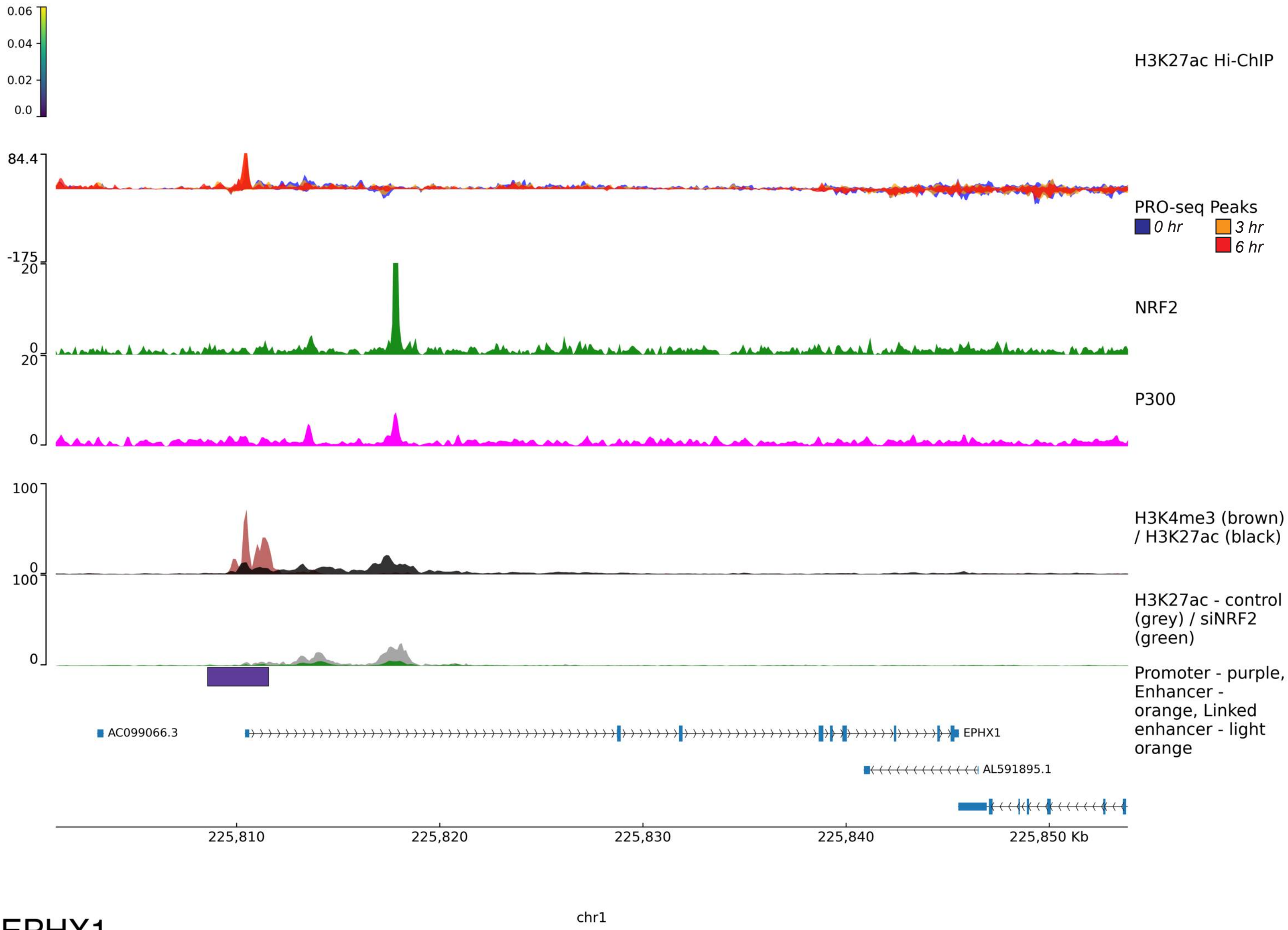
H3K27ac Hi-ChIP
0.06
0.04
0.02
0.0
84.4
-175
PRO-seq Peaks
0 hr
3 hr
6 hr
20
0
NRF2
20
0
P300
100
0
H3K4me3 (brown) / H3K27ac (black)
100
0
H3K27ac - control (grey) / siNRF2 (green)
Promoter - purple, Enhancer - orange, Linked enhancer - light orange
AC099066.3
EPHX1
AL591895.1
225,810
225,820
225,830
225,840
225,850 Kb
chr1

EPHX1

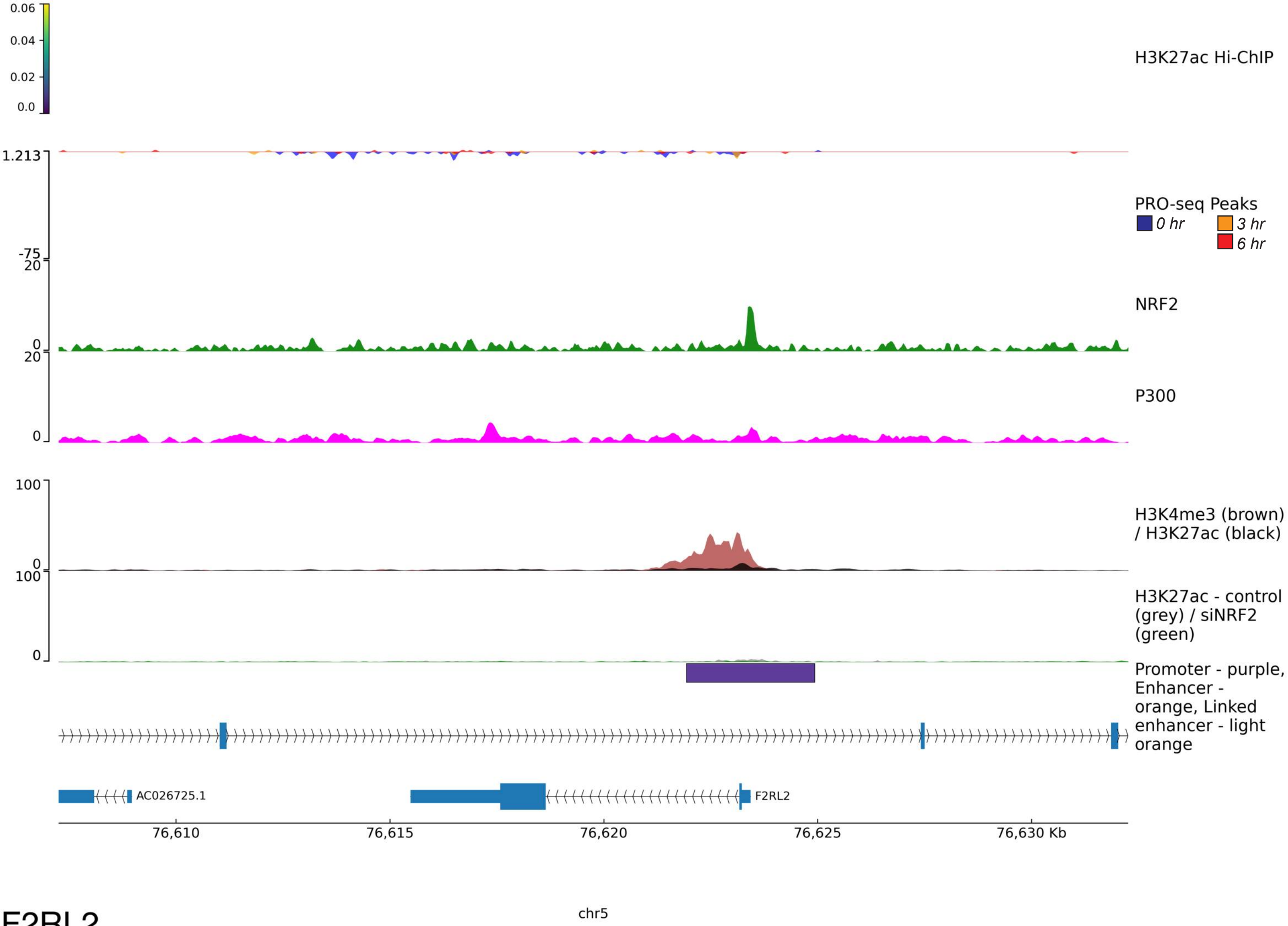
0.06
0.04
0.02
0.0
H3K27ac Hi-ChIP
1.213
-75
PRO-seq Peaks
0 hr
3 hr
6 hr
20
0
NRF2
20
0
P300
100
0
H3K4me3 (brown) / H3K27ac (black)
100
0
H3K27ac - control (grey) / siNRF2 (green)
Promoter - purple, Enhancer - orange, Linked enhancer - light orange
AC026725.1
F2RL2
76,610
76,615
76,620
76,625
76,630 Kb
chr5

F2RL2

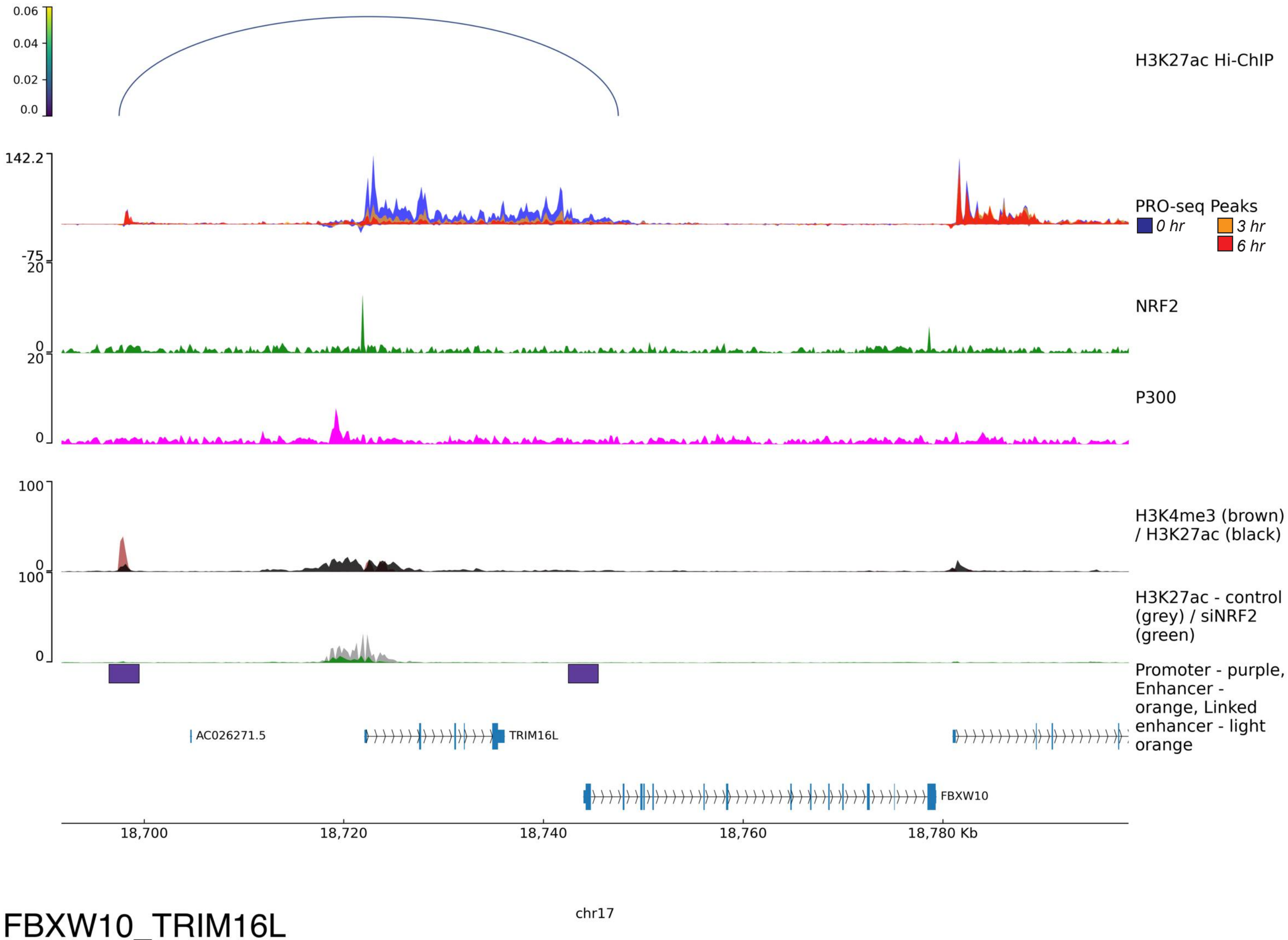

FBXW10_TRIM16L

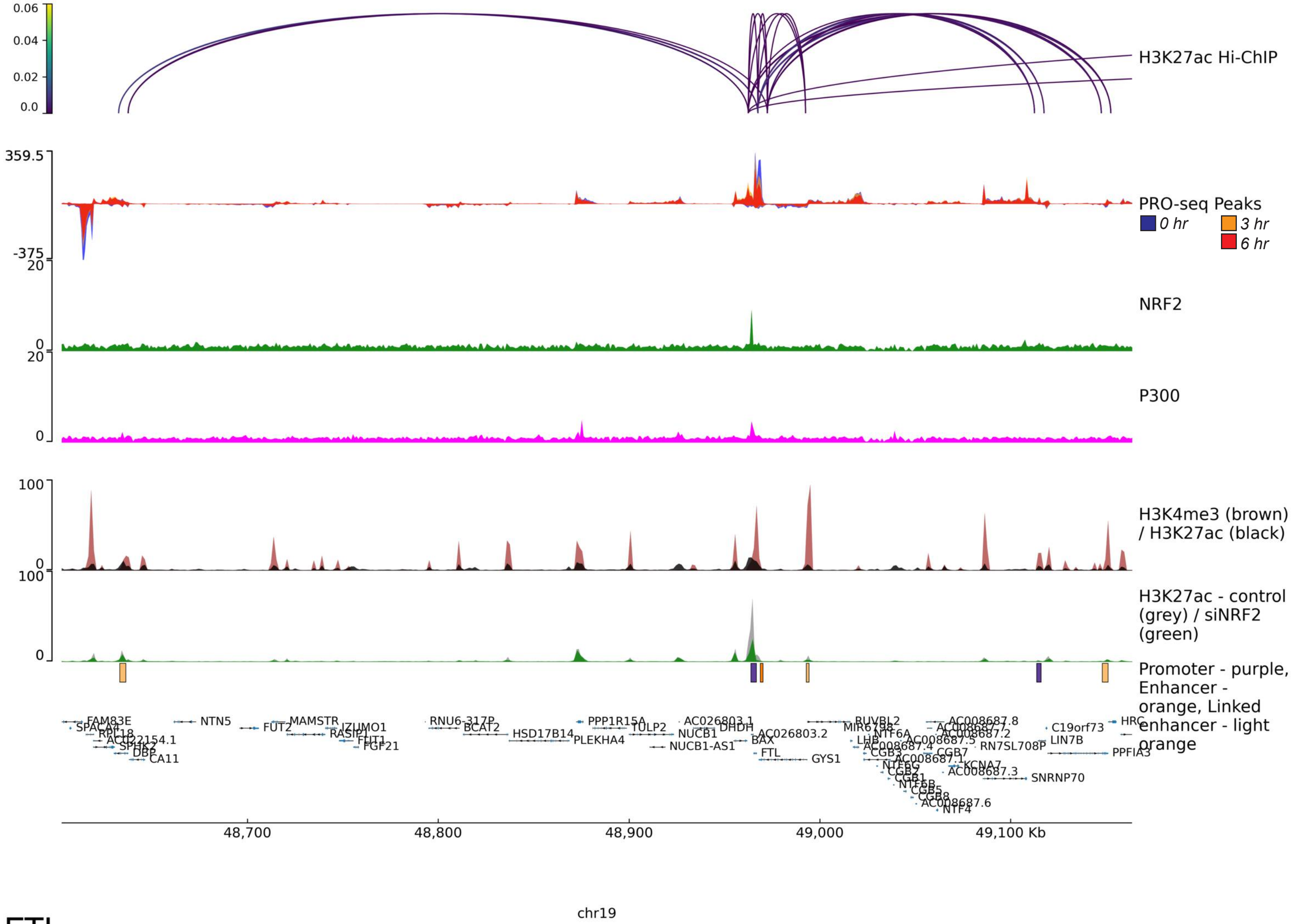

H3K27ac Hi-ChIP
PRO-seq Peaks
0 hr
3 hr
6 hr
NRF2
P300
H3K4me3 (brown) / H3K27ac (black)
H3K27ac - control (grey) / siNRF2 (green)
Promoter - purple, Enhancer - orange, Linked enhancer - light orange
48,700
48,800
48,900
49,000
49,100 Kb
chr19

FTL

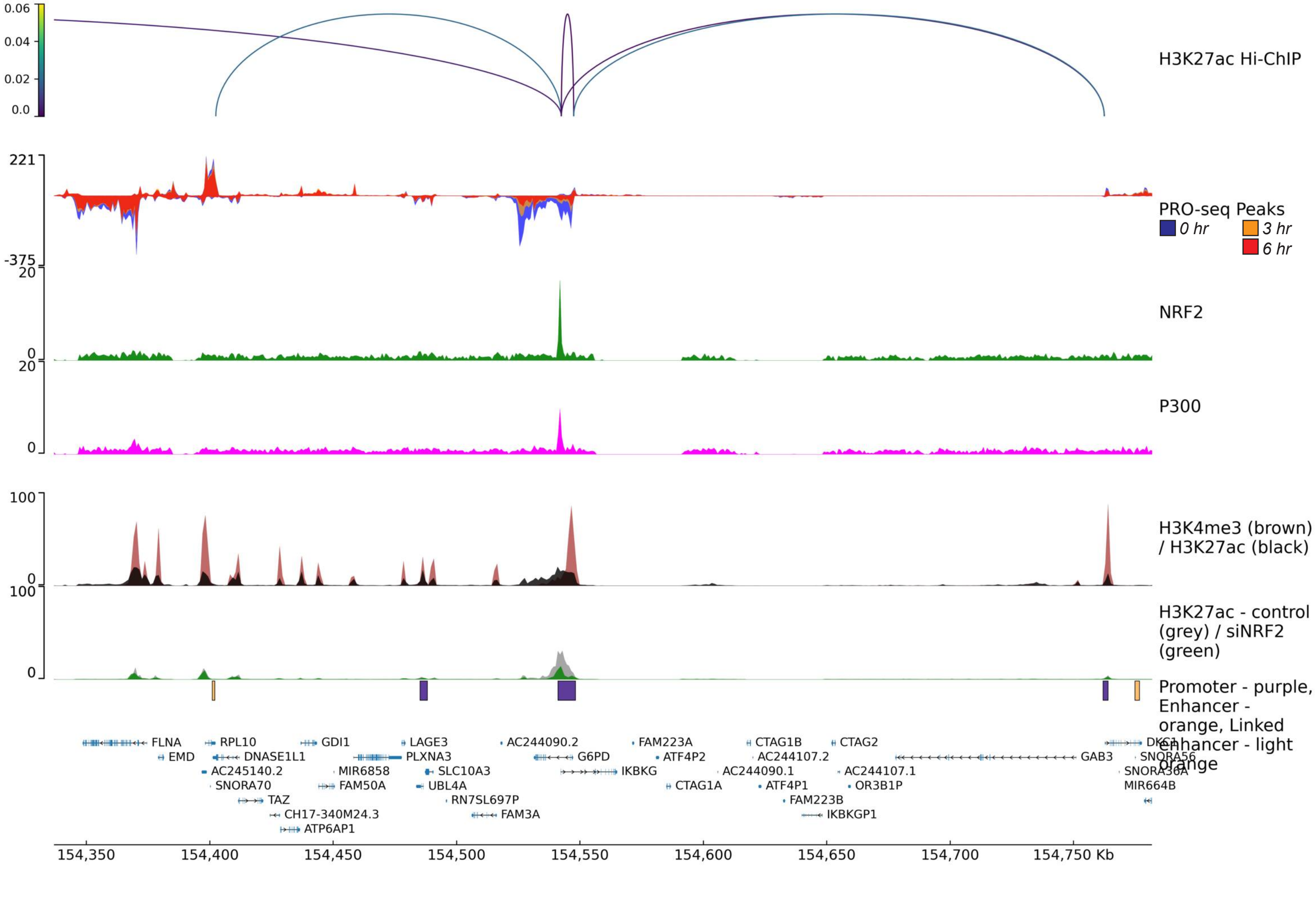

H3K27ac Hi-ChIP
PRO-seq Peaks
0 hr
3 hr
6 hr
NRF2
P300
H3K4me3 (brown) / H3K27ac (black)
H3K27ac - control (grey) / siNRF2 (green)
Promoter - purple, Enhancer - orange, Linked enhancer - light orange
FLNA
EMD
RPL10
DNASE1L1
AC245140.2
SNORA70
TAZ
CH17-340M24.3
ATP6AP1
GDI1
MIR6858
FAM50A
LAGE3
PLXNA3
SLC10A3
UBL4A
RN7SL697P
FAM3A
AC244090.2
G6PD
IKBKG
FAM223A
ATF4P2
AC244090.1
CTAG1A
CTAG1B
AC244107.2
ATF4P1
FAM223B
IKBKGP1
CTAG2
AC244107.1
OR3B1P
GAB3
DKC1
SNORA56
SNORA36A
MIR664B
154,350
154,400
154,450
154,500
154,550
154,600
154,650
154,700
154,750 Kb
chrX

G6PD

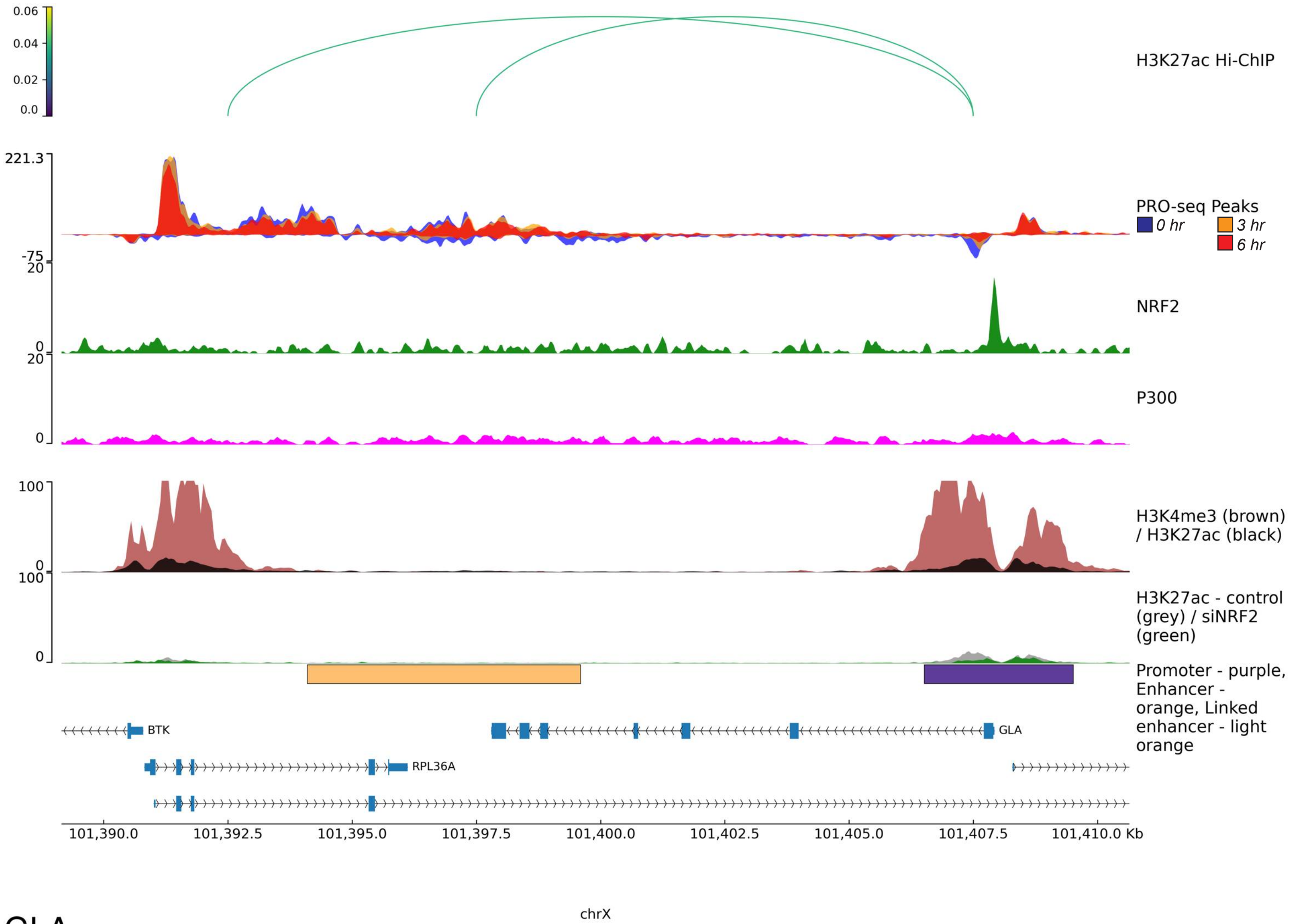
0.06
0.04
0.02
0.0
H3K27ac Hi-ChIP
221.3
-75
PRO-seq Peaks
0 hr
3 hr
6 hr
20
0
NRF2
20
0
P300
100
0
H3K4me3 (brown) / H3K27ac (black)
100
0
H3K27ac - control (grey) / siNRF2 (green)
Promoter - purple, Enhancer - orange, Linked enhancer - light orange
BTK
GLA
RPL36A
101,390.0
101,392.5
101,395.0
101,397.5
101,400.0
101,402.5
101,405.0
101,407.5
101,410.0 Kb
chrX

GLA

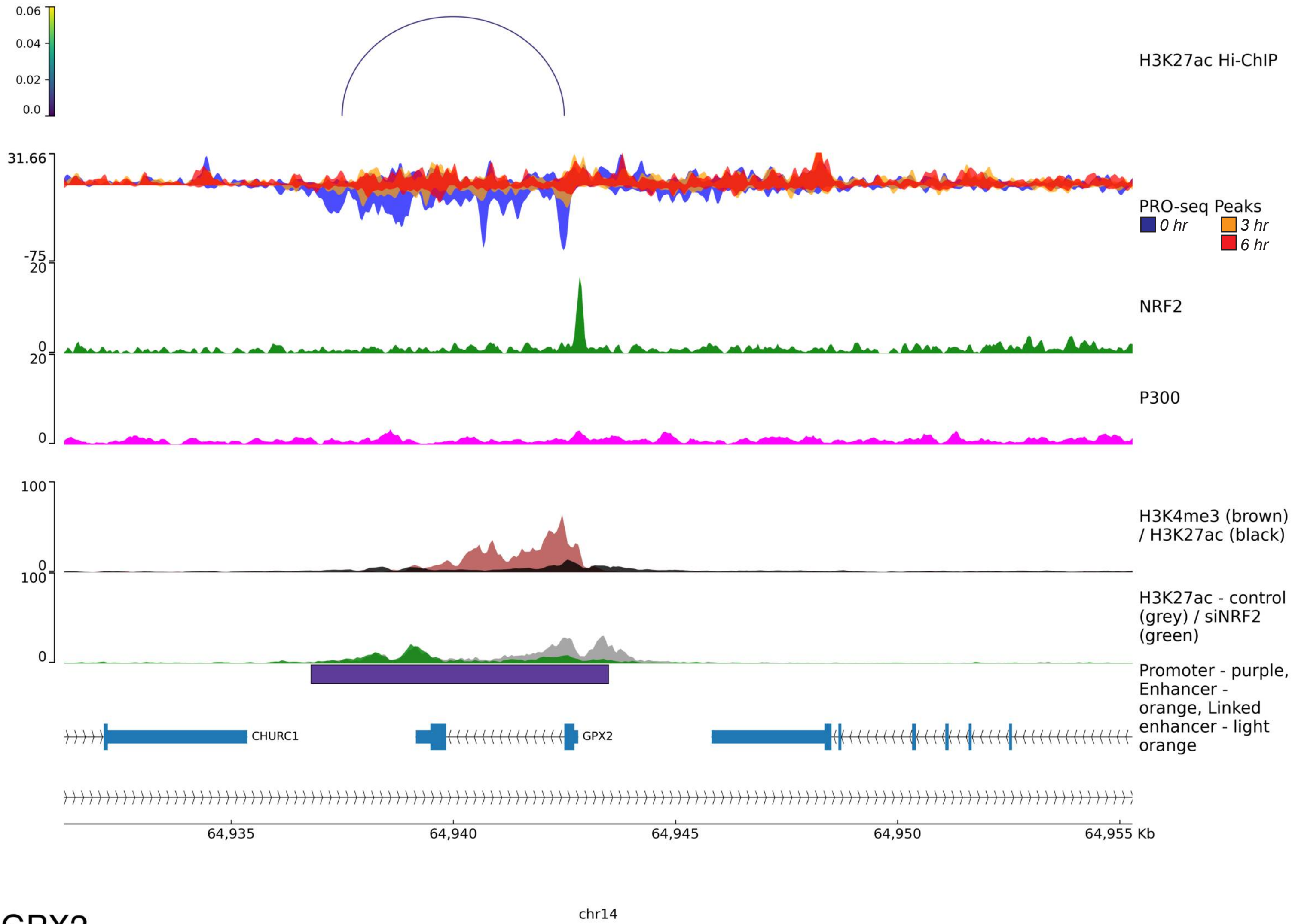

0.06
0.04
0.02
0.0
H3K27ac Hi-ChIP
31.66
-75
PRO-seq Peaks
0 hr
3 hr
6 hr
20
0
NRF2
20
0
P300
100
0
H3K4me3 (brown) / H3K27ac (black)
100
0
H3K27ac - control (grey) / siNRF2 (green)
Promoter - purple, Enhancer - orange, Linked enhancer - light orange
CHURC1
GPX2
64,935
64,940
64,945
64,950
64,955 Kb
chr14

GPX2

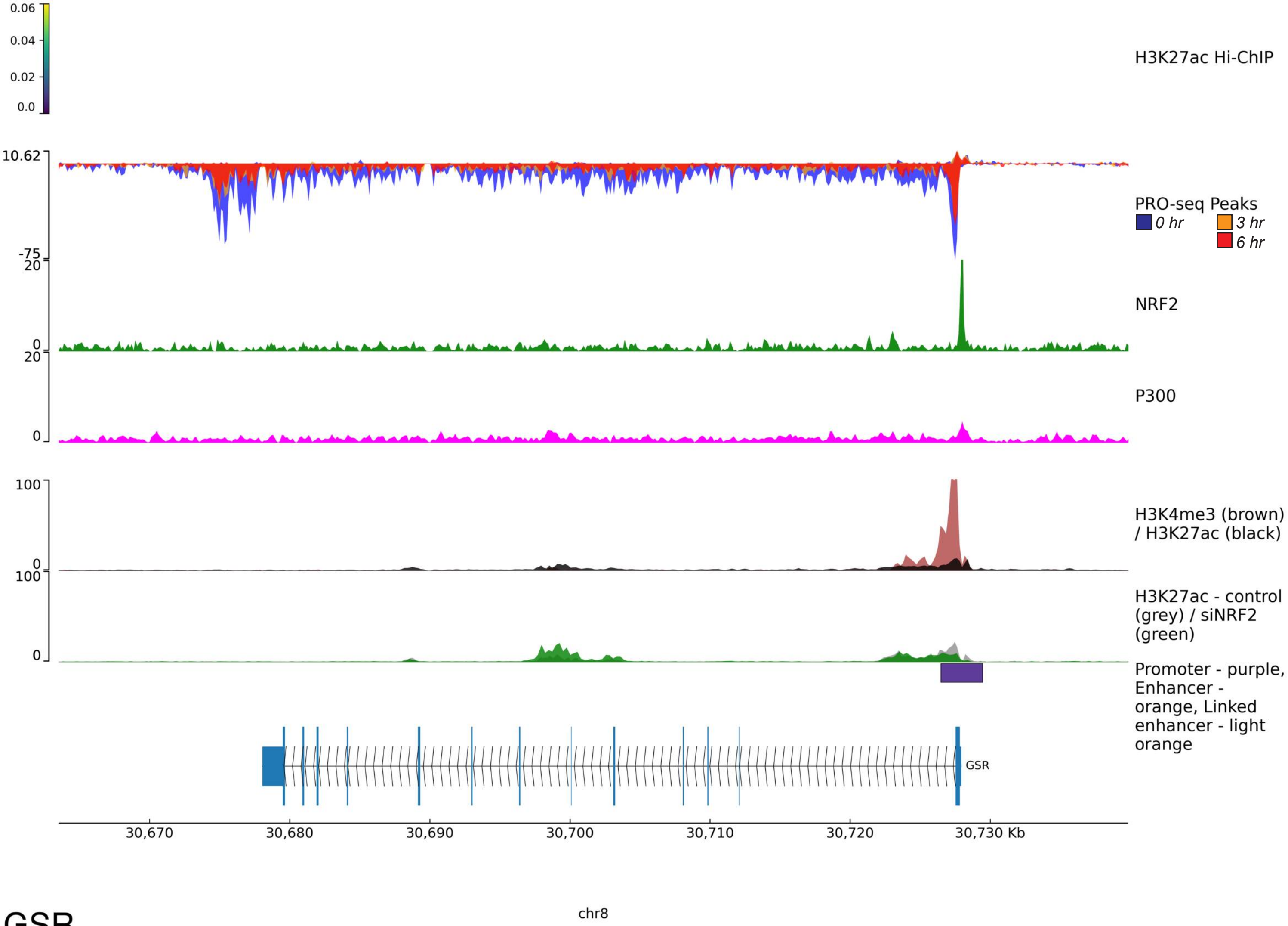
0.06
0.04
0.02
0.0
H3K27ac Hi-ChIP
10.62
-75
PRO-seq Peaks
0 hr
3 hr
6 hr
20
0
NRF2
20
0
P300
100
0
H3K4me3 (brown) / H3K27ac (black)
100
0
H3K27ac - control (grey) / siNRF2 (green)
Promoter - purple, Enhancer - orange, Linked enhancer - light orange
GSR
30,670
30,680
30,690
30,700
30,710
30,720
30,730 Kb
chr8

GSR

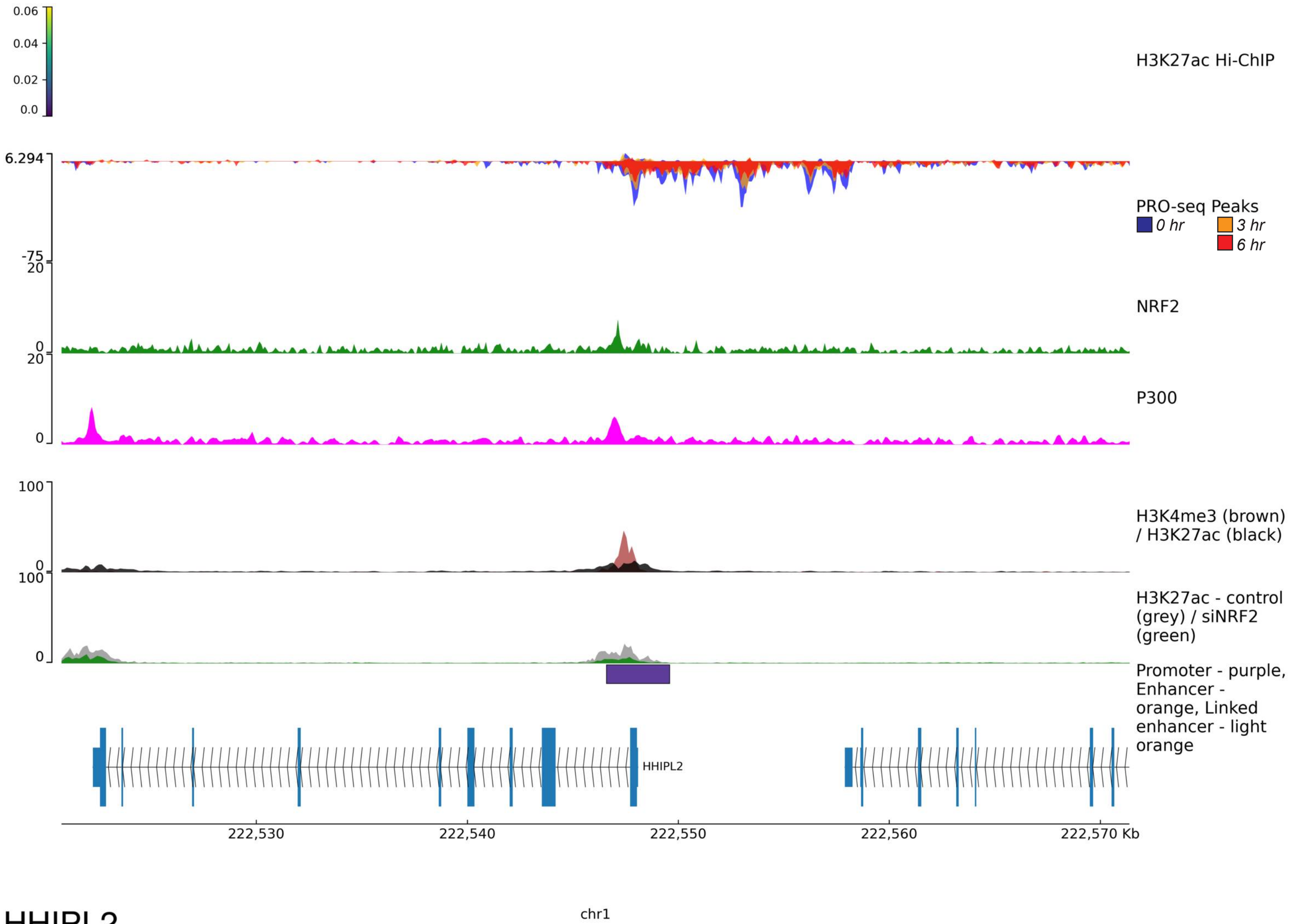

H3K27ac Hi-ChIP
0.06
0.04
0.02
0.0
6.294
-75
PRO-seq Peaks
0 hr
3 hr
6 hr
20
0
NRF2
20
0
P300
100
0
H3K4me3 (brown) / H3K27ac (black)
100
0
H3K27ac - control (grey) / siNRF2 (green)
Promoter - purple, Enhancer - orange, Linked enhancer - light orange
HHIPL2
222,530
222,540
222,550
222,560
222,570 Kb
chr1

HHIPL2

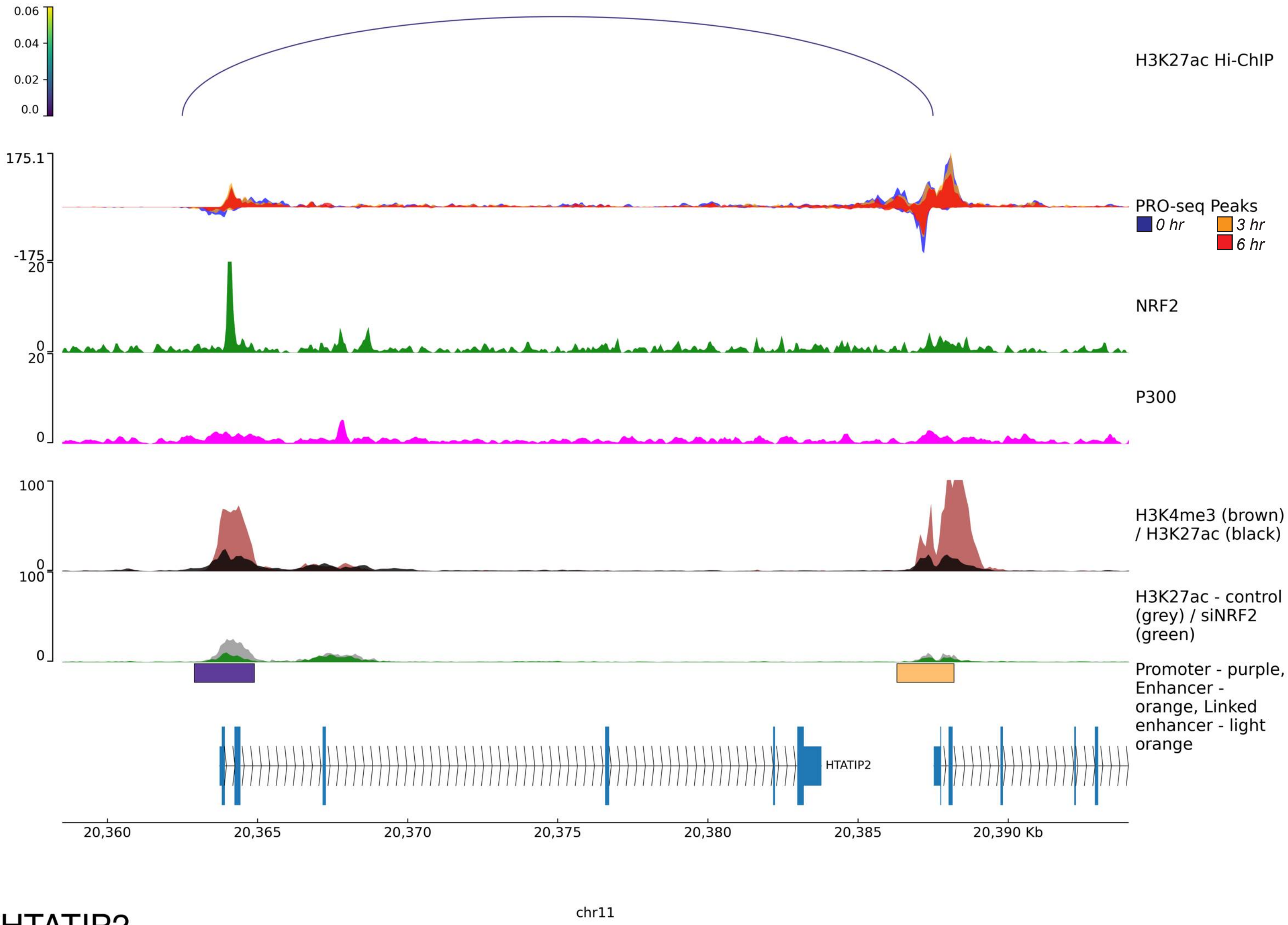
0.06
0.04
0.02
0.0
H3K27ac Hi-ChIP
175.1
-175
PRO-seq Peaks
0 hr
3 hr
6 hr
20
0
NRF2
20
0
P300
100
0
H3K4me3 (brown) / H3K27ac (black)
100
0
H3K27ac - control (grey) / siNRF2 (green)
Promoter - purple, Enhancer - orange, Linked enhancer - light orange
HTATIP2
20,360
20,365
20,370
20,375
20,380
20,385
20,390 Kb
chr11

HTATIP2

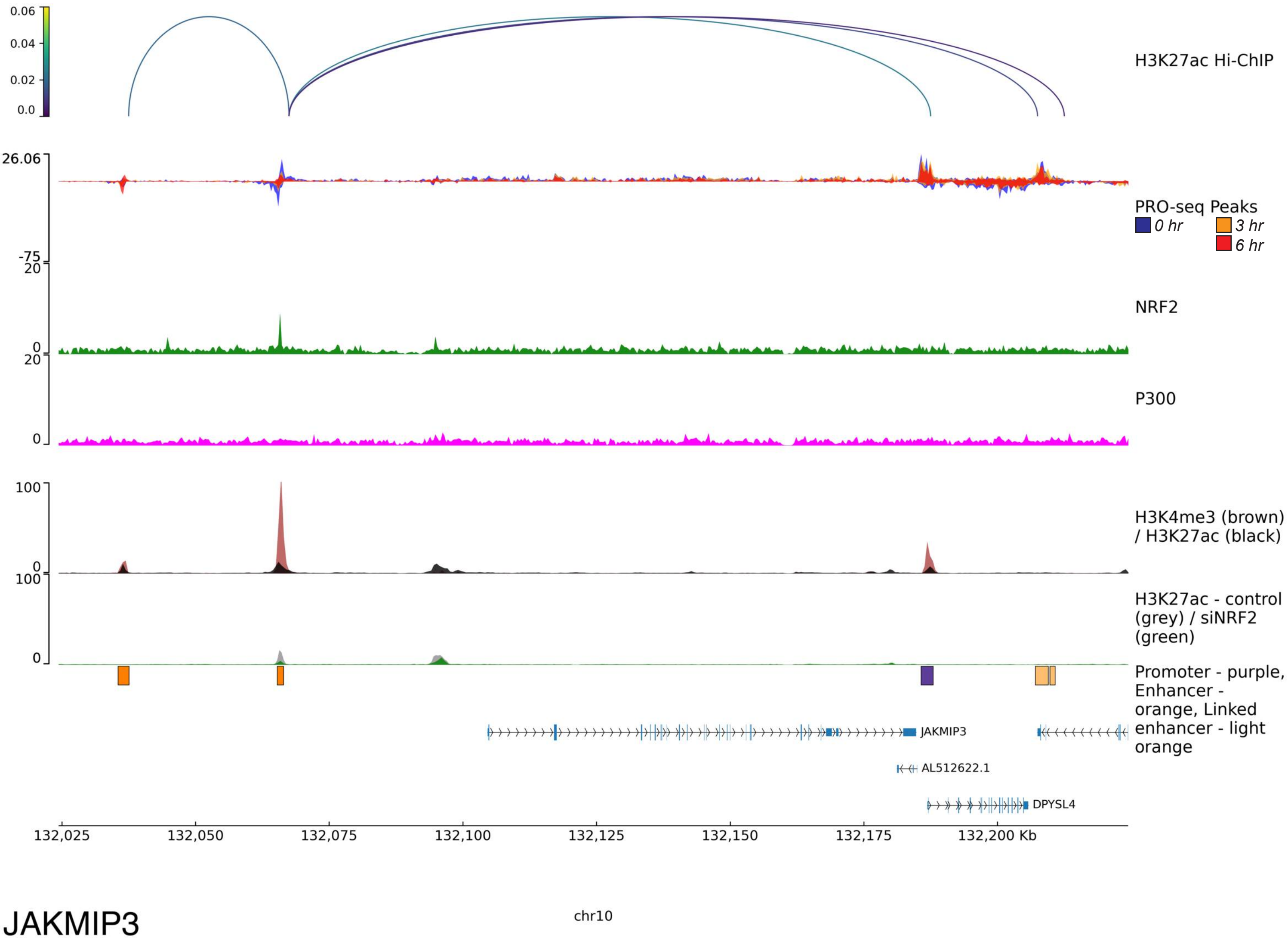
0.06
0.04
0.02
0.0
H3K27ac Hi-ChIP
26.06
-75
PRO-seq Peaks
0 hr
3 hr
6 hr
20
0
NRF2
20
0
P300
100
0
H3K4me3 (brown) / H3K27ac (black)
100
0
H3K27ac - control (grey) / siNRF2 (green)
Promoter - purple, Enhancer - orange, Linked enhancer - light orange
JAKMIP3
AL512622.1
DPYSL4
132,025
132,050
132,075
132,100
132,125
132,150
132,175
132,200 Kb
chr10

JAKMIP3

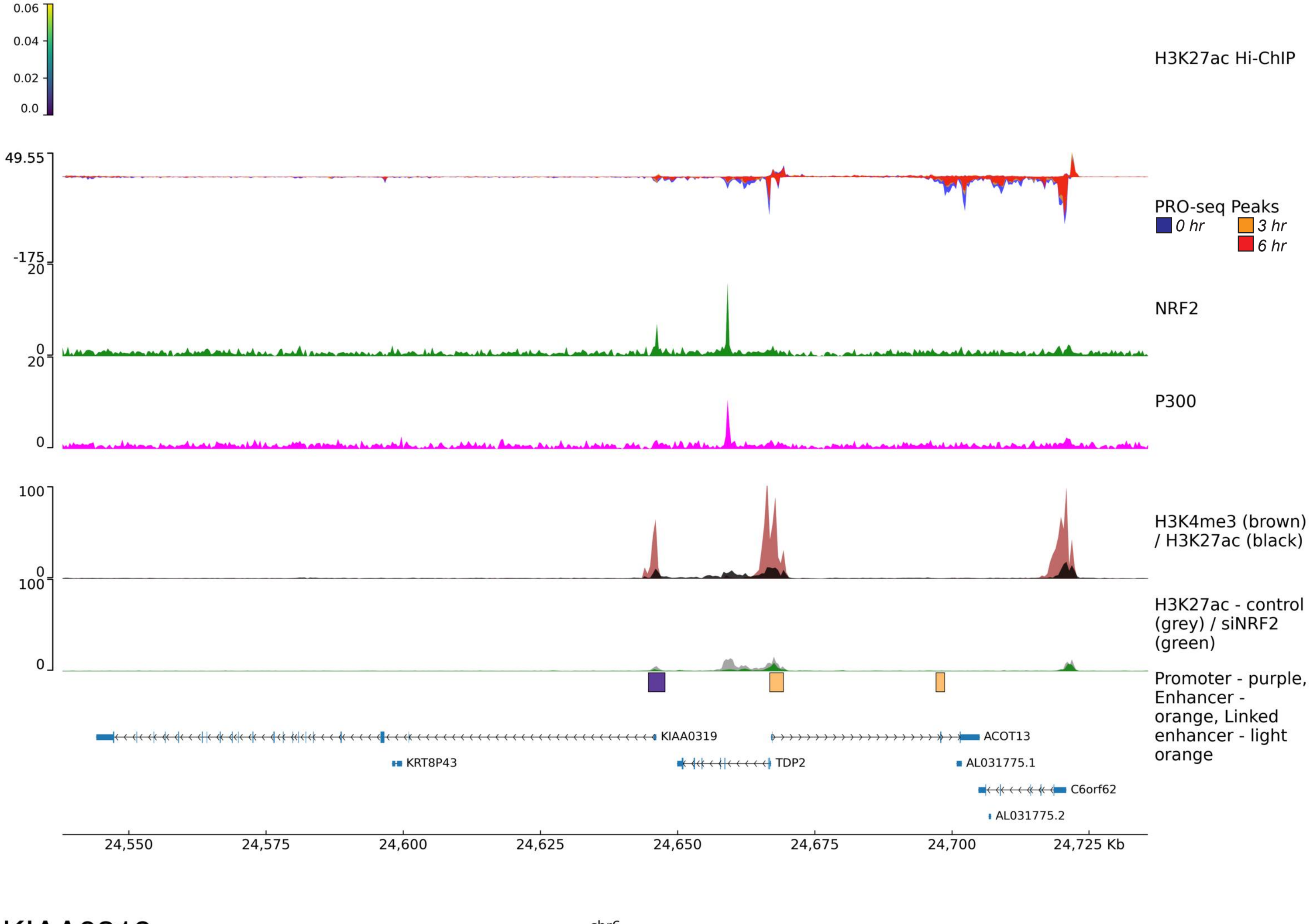

0.06
0.04
0.02
0.0
H3K27ac Hi-ChIP
49.55
-175
PRO-seq Peaks
0 hr
3 hr
6 hr
20
0
NRF2
20
0
P300
100
0
H3K4me3 (brown) / H3K27ac (black)
100
0
H3K27ac - control (grey) / siNRF2 (green)
Promoter - purple, Enhancer - orange, Linked enhancer - light orange
KIAA0319
ACOT13
KRT8P43
TDP2
AL031775.1
C6orf62
AL031775.2
24,550
24,575
24,600
24,625
24,650
24,675
24,700
24,725 Kb
chr6

KIAA0319

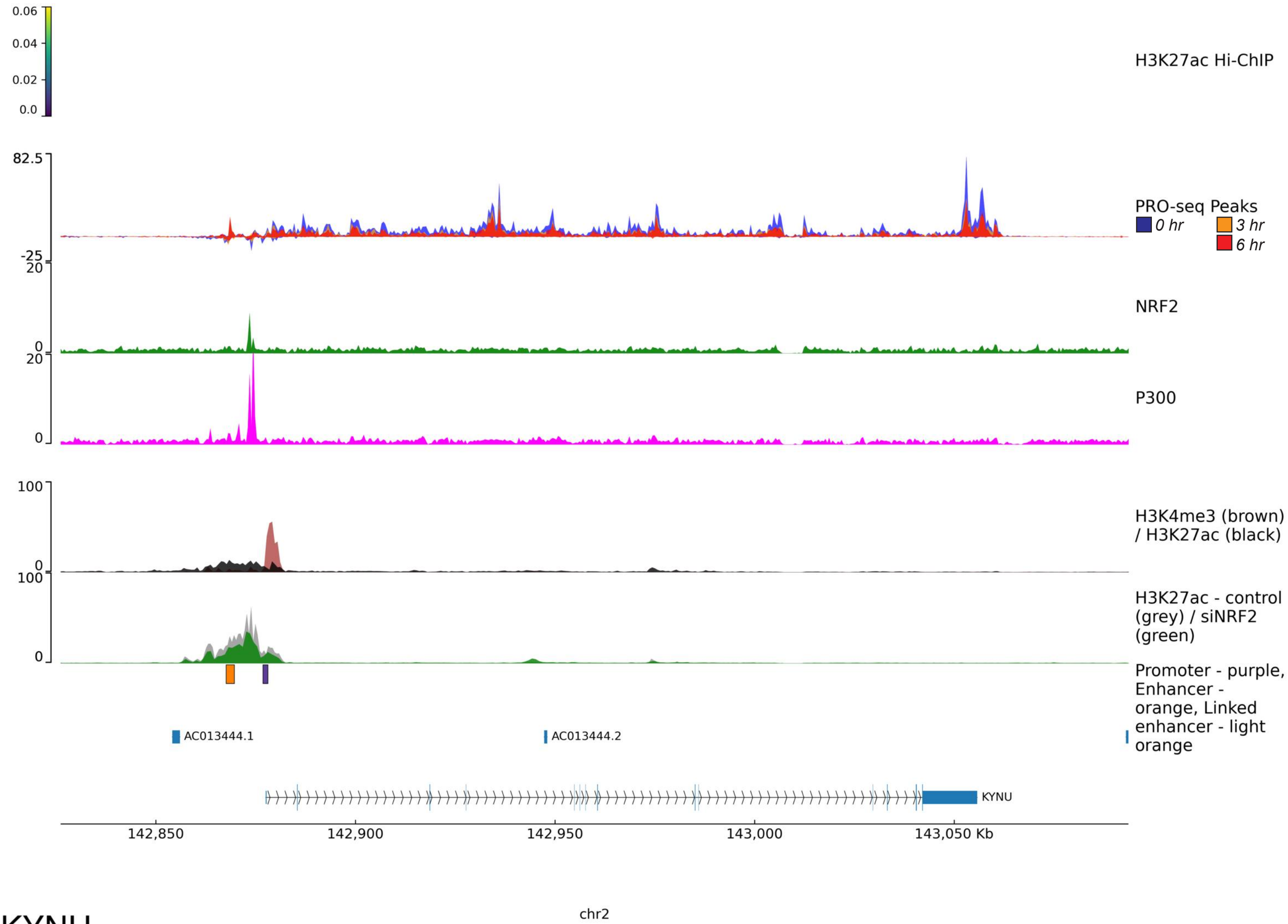
0.06
0.04
0.02
0.0
H3K27ac Hi-ChIP
82.5
-25
PRO-seq Peaks
0 hr
3 hr
6 hr
20
0
NRF2
20
0
P300
100
0
H3K4me3 (brown) / H3K27ac (black)
100
0
H3K27ac - control (grey) / siNRF2 (green)
Promoter - purple, Enhancer - orange, Linked enhancer - light orange
AC013444.1
AC013444.2
KYNU
142,850
142,900
142,950
143,000
143,050 Kb
chr2

KYNU

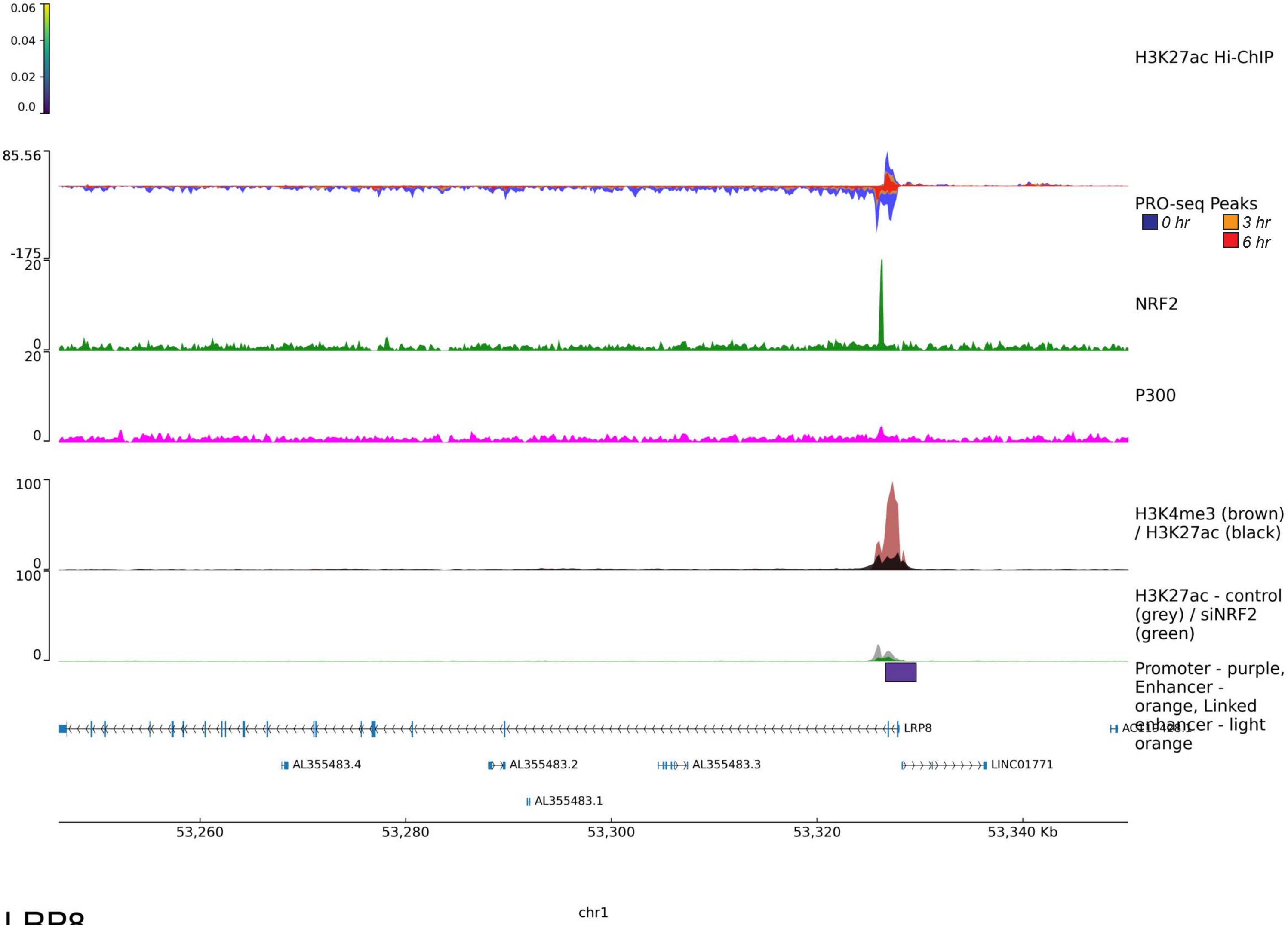

0.06
0.04
0.02
0.0
H3K27ac Hi-ChIP
85.56
-175
PRO-seq Peaks
0 hr
3 hr
6 hr
20
0
NRF2
20
0
P300
100
0
H3K4me3 (brown) / H3K27ac (black)
100
0
H3K27ac - control (grey) / siNRF2 (green)
Promoter - purple, Enhancer - orange, Linked enhancer - light orange
LRP8
AL355483.4
AL355483.2
AL355483.3
LINC01771
AL355483.1
53,260
53,280
53,300
53,320
53,340 Kb
chr1

LRP8

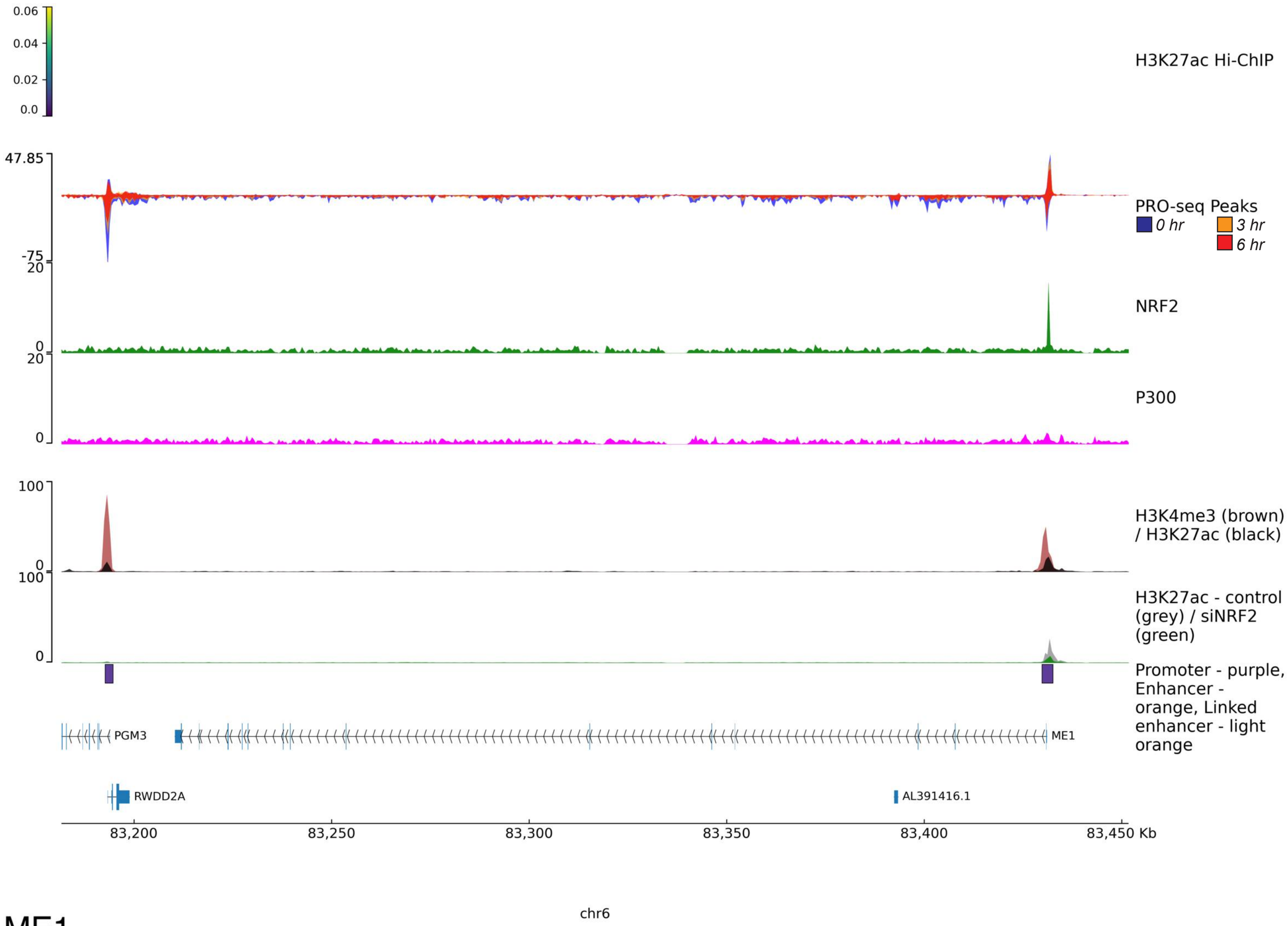
0.06
0.04
0.02
0.0
H3K27ac Hi-ChIP
47.85
-75
PRO-seq Peaks
0 hr
3 hr
6 hr
20
0
NRF2
20
0
P300
100
0
H3K4me3 (brown) / H3K27ac (black)
100
0
H3K27ac - control (grey) / siNRF2 (green)
Promoter - purple, Enhancer - orange, Linked enhancer - light orange
PGM3
ME1
RWDD2A
AL391416.1
83,200
83,250
83,300
83,350
83,400
83,450 Kb
chr6

ME1

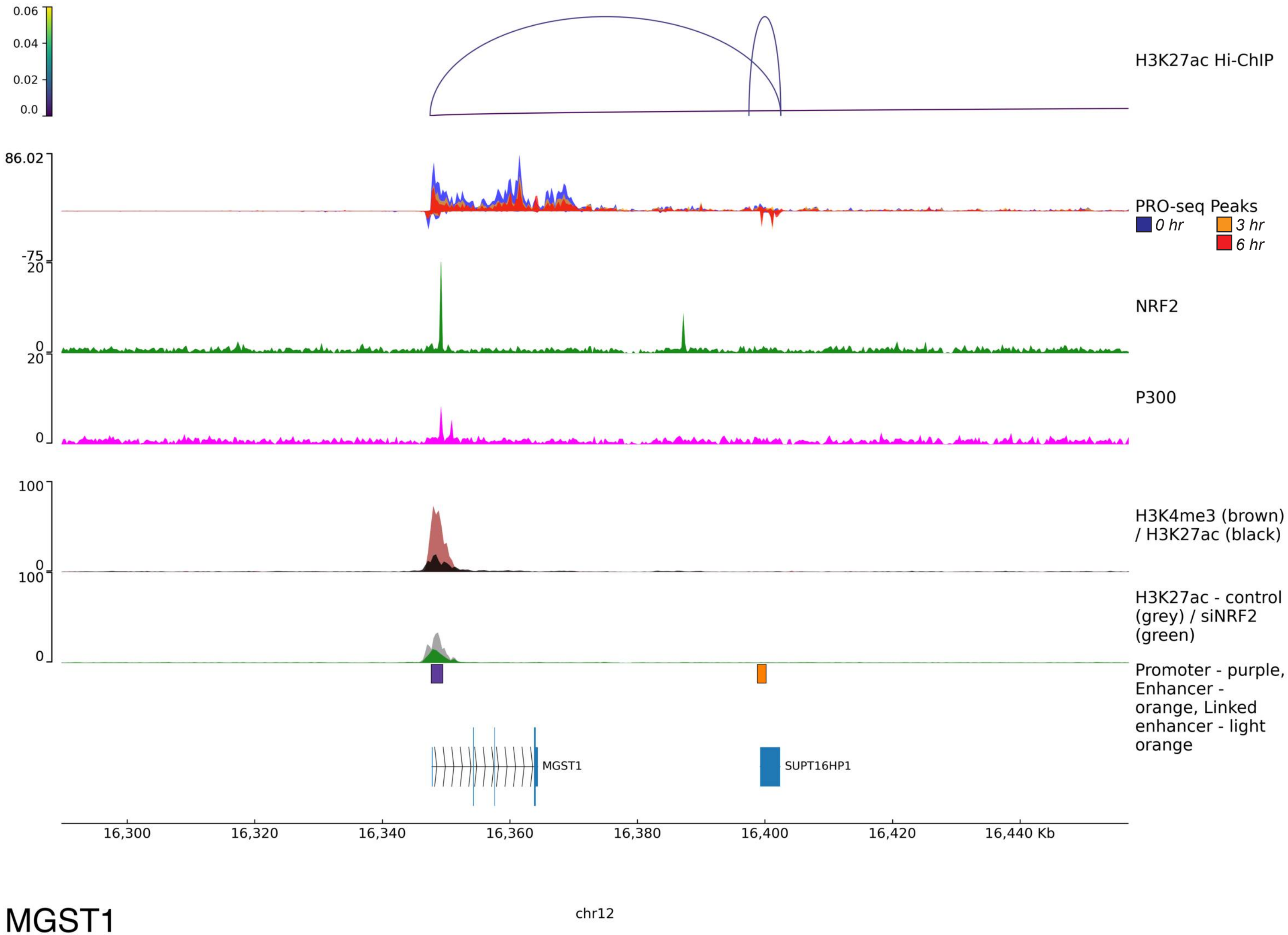
0.06
0.04
0.02
0.0
H3K27ac Hi-ChIP
86.02
-75
PRO-seq Peaks
0 hr
3 hr
6 hr
20
0
NRF2
20
0
P300
100
0
H3K4me3 (brown) / H3K27ac (black)
100
0
H3K27ac - control (grey) / siNRF2 (green)
Promoter - purple, Enhancer - orange, Linked enhancer - light orange
MGST1
SUPT16HP1
16,300
16,320
16,340
16,360
16,380
16,400
16,420
16,440 Kb
chr12

MGST1

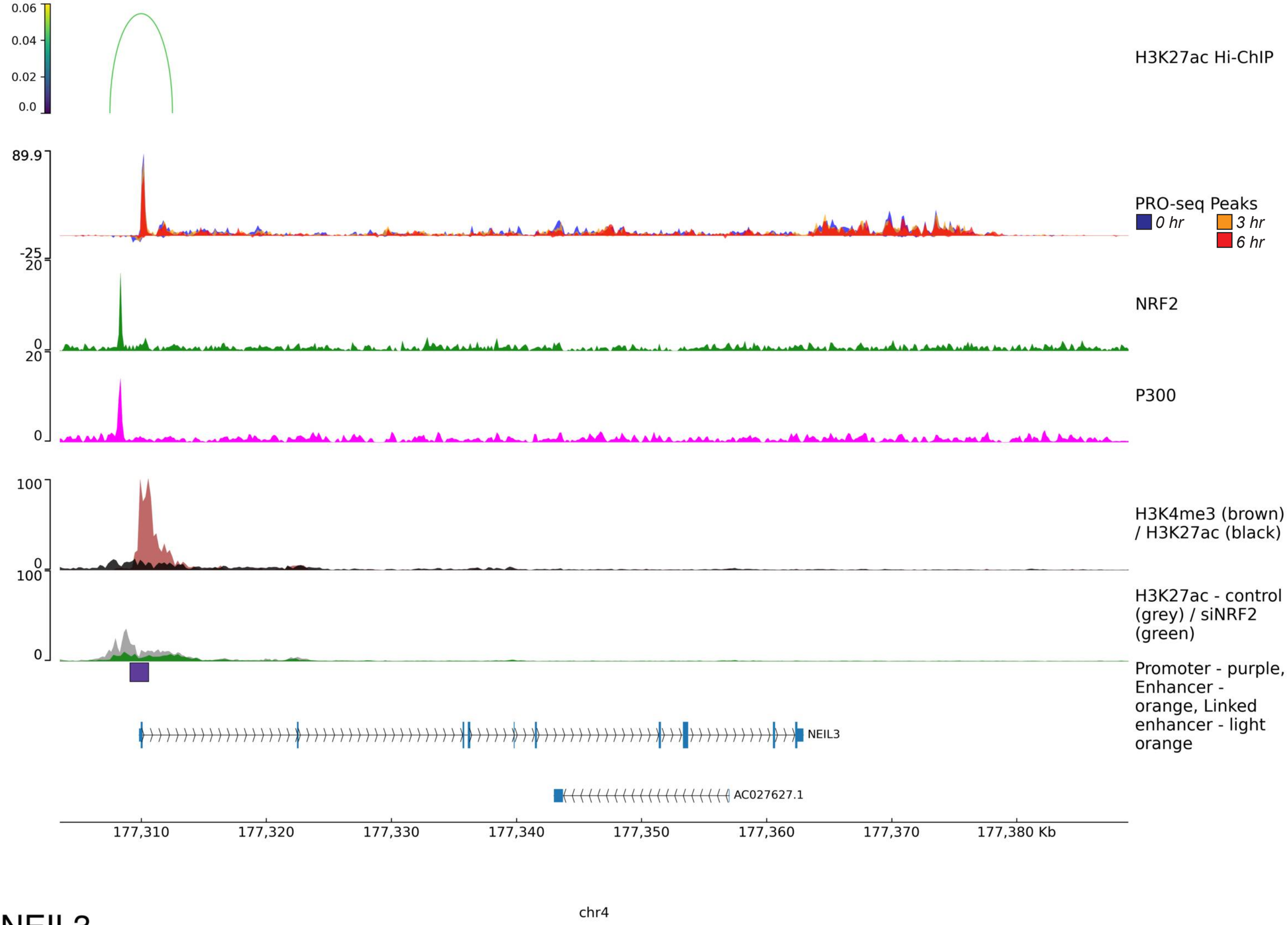

0.06
0.04
0.02
0.0
H3K27ac Hi-ChIP
89.9
-25
PRO-seq Peaks
0 hr
3 hr
6 hr
20
0
NRF2
20
0
P300
100
0
H3K4me3 (brown) / H3K27ac (black)
100
0
H3K27ac - control (grey) / siNRF2 (green)
Promoter - purple, Enhancer - orange, Linked enhancer - light orange
NEIL3
AC027627.1
177,310
177,320
177,330
177,340
177,350
177,360
177,370
177,380 Kb
chr4

NEIL3

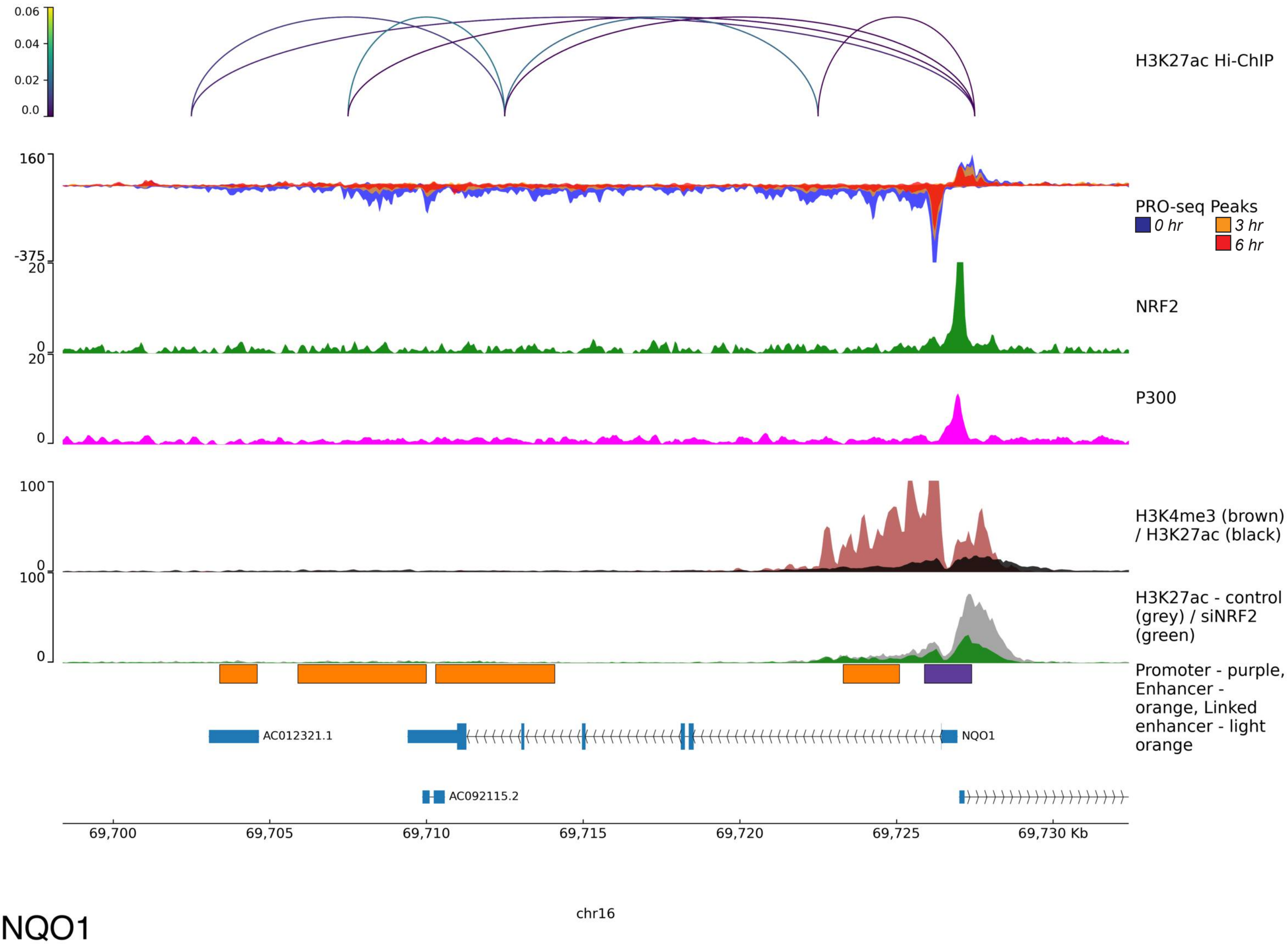
0.06
0.04
0.02
0.0
H3K27ac Hi-ChIP
160
-375
PRO-seq Peaks
0 hr
3 hr
6 hr
20
0
NRF2
20
0
P300
100
0
H3K4me3 (brown) / H3K27ac (black)
100
0
H3K27ac - control (grey) / siNRF2 (green)
Promoter - purple, Enhancer - orange, Linked enhancer - light orange
AC012321.1
NQO1
AC092115.2
69,700
69,705
69,710
69,715
69,720
69,725
69,730 Kb
chr16

NQO1

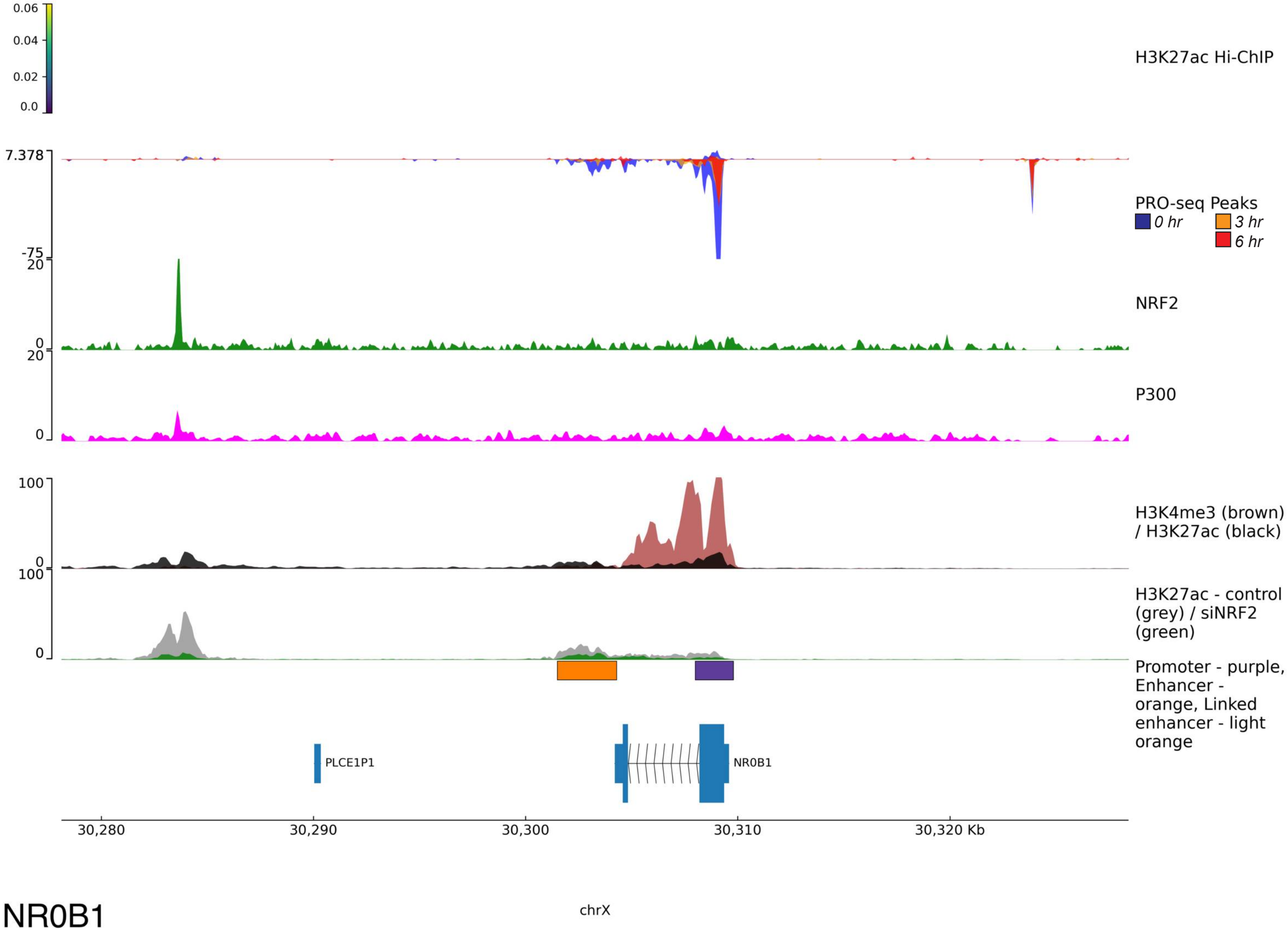

0.06
0.04
0.02
0.0
H3K27ac Hi-ChIP
7.378
-75
PRO-seq Peaks
0 hr
3 hr
6 hr
20
0
NRF2
20
0
P300
100
0
H3K4me3 (brown) / H3K27ac (black)
100
0
H3K27ac - control (grey) / siNRF2 (green)
Promoter - purple, Enhancer - orange, Linked enhancer - light orange
PLCE1P1
NR0B1
30,280
30,290
30,300
30,310
30,320 Kb
chrX

NR0B1

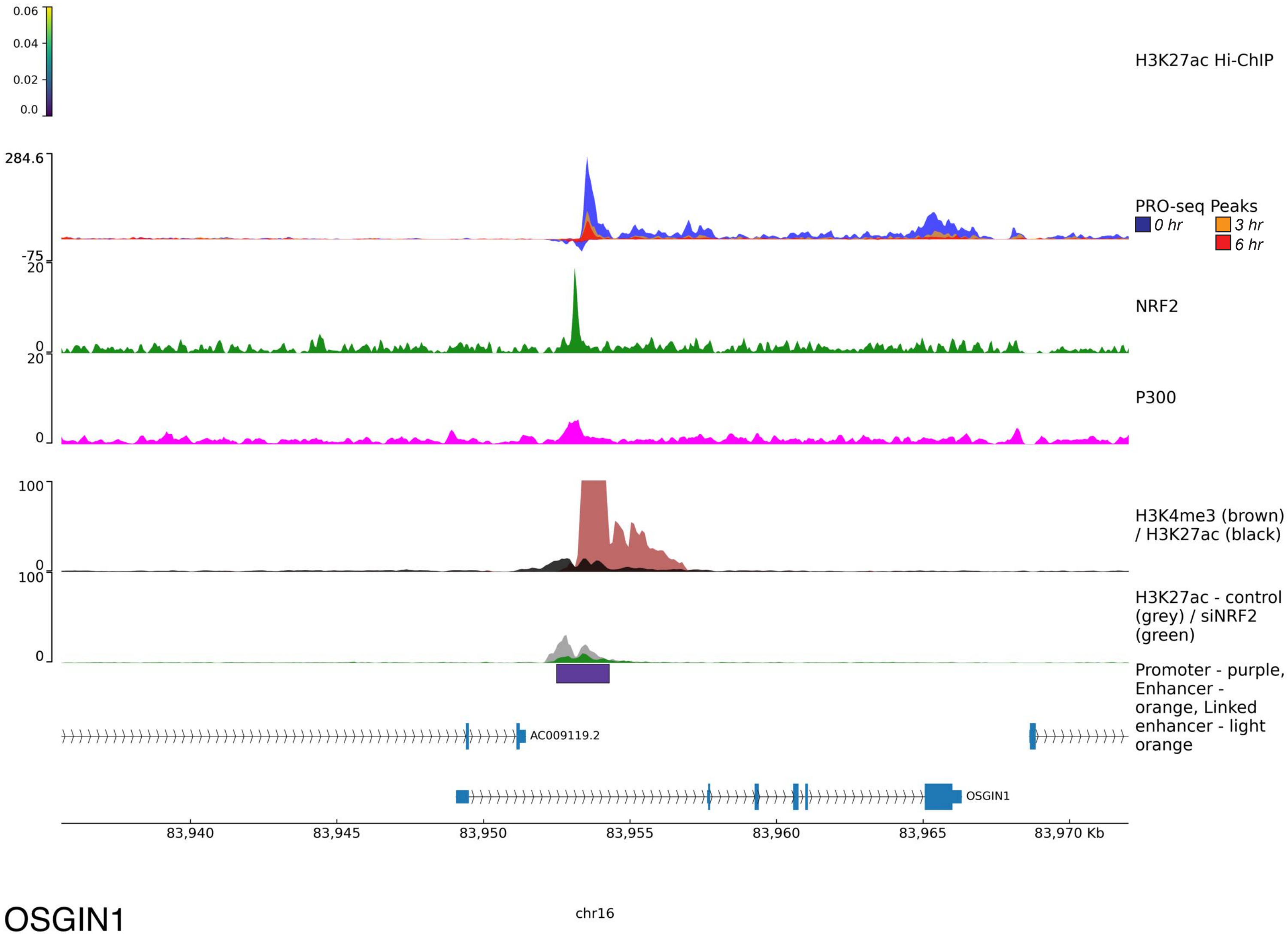
0.06
0.04
0.02
0.0
H3K27ac Hi-ChIP
284.6
-75
PRO-seq Peaks
0 hr
3 hr
6 hr
20
0
NRF2
20
0
P300
100
0
H3K4me3 (brown) / H3K27ac (black)
100
0
H3K27ac - control (grey) / siNRF2 (green)
Promoter - purple, Enhancer - orange, Linked enhancer - light orange
AC009119.2
OSGIN1
83,940
83,945
83,950
83,955
83,960
83,965
83,970 Kb
chr16

OSGIN1

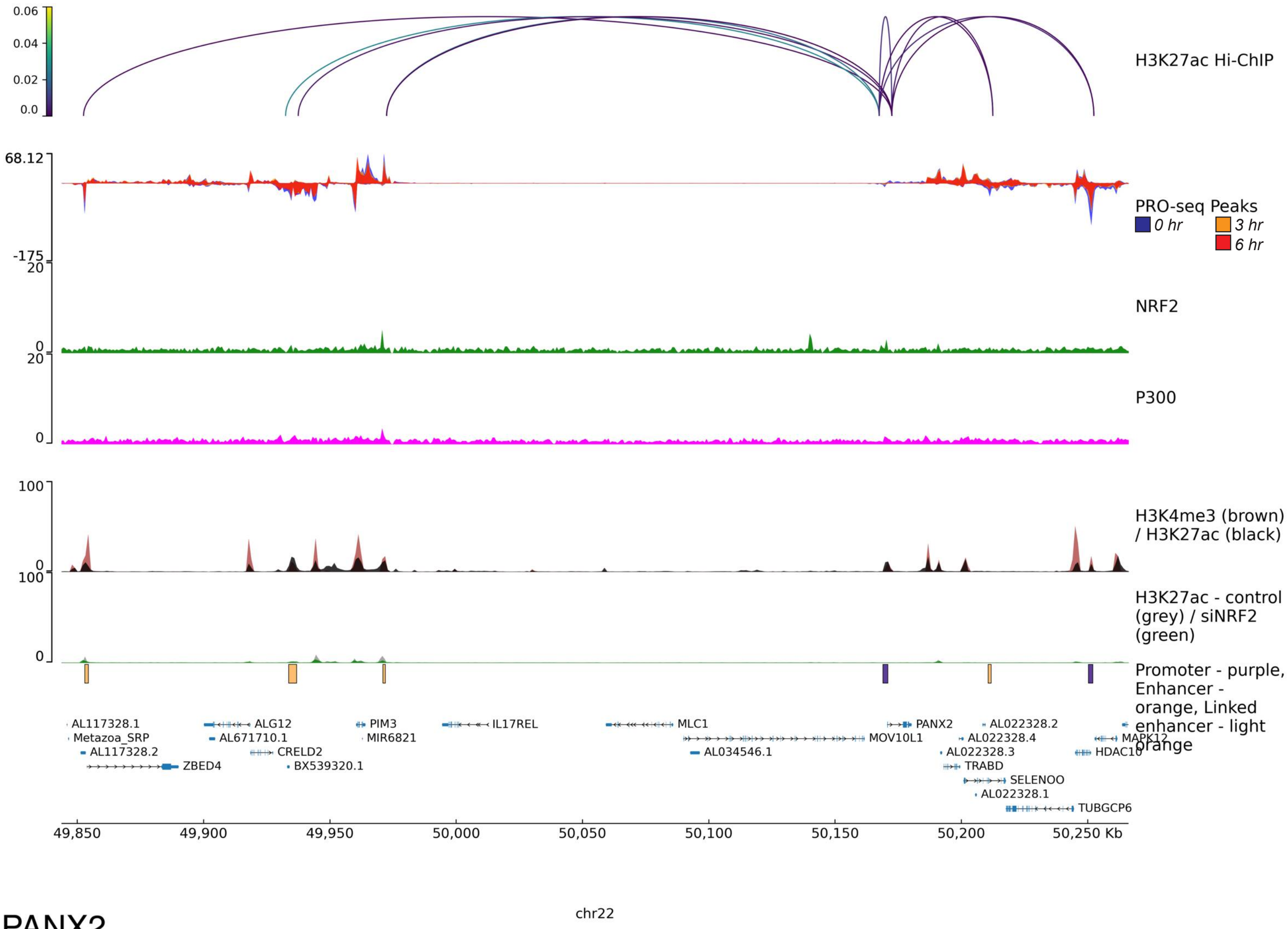
H3K27ac Hi-ChIP
PRO-seq Peaks
0 hr
3 hr
6 hr
NRF2
P300
H3K4me3 (brown) / H3K27ac (black)
H3K27ac - control (grey) / siNRF2 (green)
Promoter - purple, Enhancer - orange, Linked enhancer - light orange
AL117328.1
Metazoa_SRP
AL117328.2
ZBED4
ALG12
AL671710.1
CRELD2
BX539320.1
PIM3
MIR6821
IL17REL
MLC1
MOV10L1
AL034546.1
PANX2
AL022328.2
AL022328.4
AL022328.3
TRABD
SELENOO
AL022328.1
TUBGCP6
MAPK12
HDAC10
49,850
49,900
49,950
50,000
50,050
50,100
50,150
50,200
50,250 Kb
chr22

PANX2

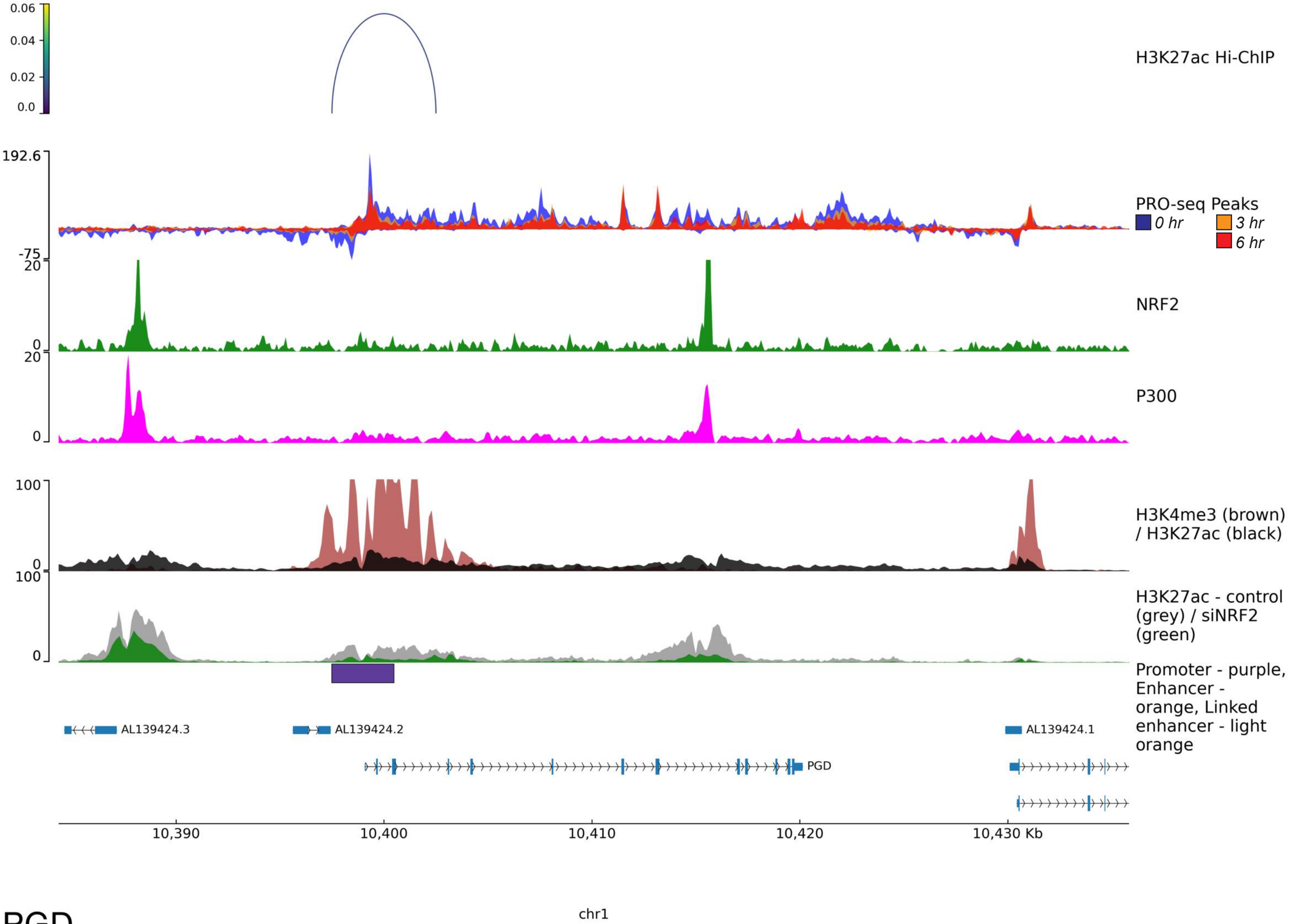
H3K27ac Hi-ChIP
PRO-seq Peaks
0 hr
3 hr
6 hr
NRF2
P300
H3K4me3 (brown) / H3K27ac (black)
H3K27ac - control (grey) / siNRF2 (green)
Promoter - purple, Enhancer - orange, Linked enhancer - light orange
AL139424.3
AL139424.2
AL139424.1
PGD
10,390
10,400
10,410
10,420
10,430 Kb
chr1

PGD

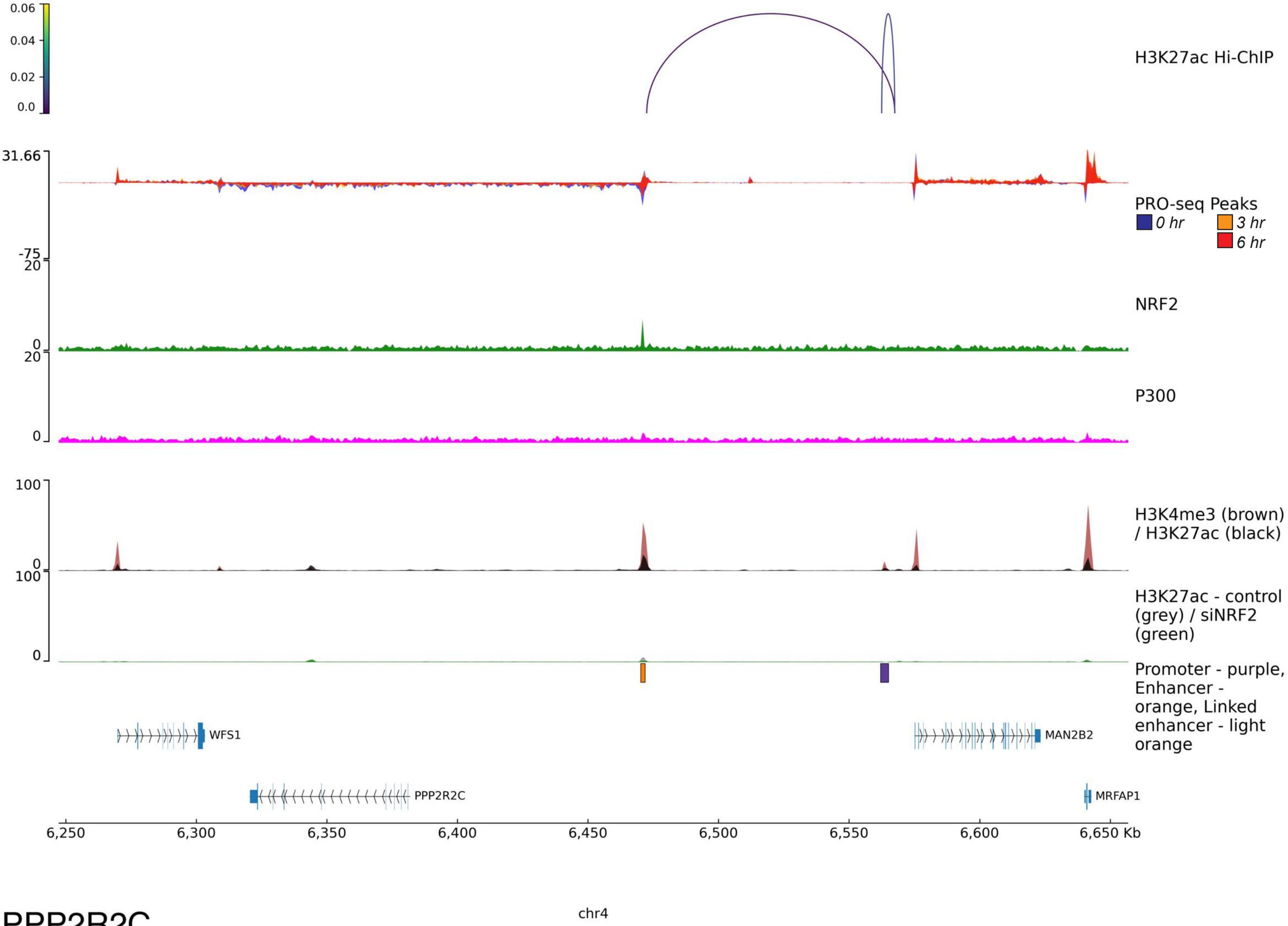
0.06
0.04
0.02
0.0
H3K27ac Hi-ChIP
31.66
-75
PRO-seq Peaks
0 hr
3 hr
6 hr
20
0
NRF2
20
0
P300
100
0
H3K4me3 (brown) / H3K27ac (black)
100
0
H3K27ac - control (grey) / siNRF2 (green)
Promoter - purple, Enhancer - orange, Linked enhancer - light orange
WFS1
MAN2B2
PPP2R2C
MRFAP1
6,250
6,300
6,350
6,400
6,450
6,500
6,550
6,600
6,650 Kb
chr4

PPP2R2C

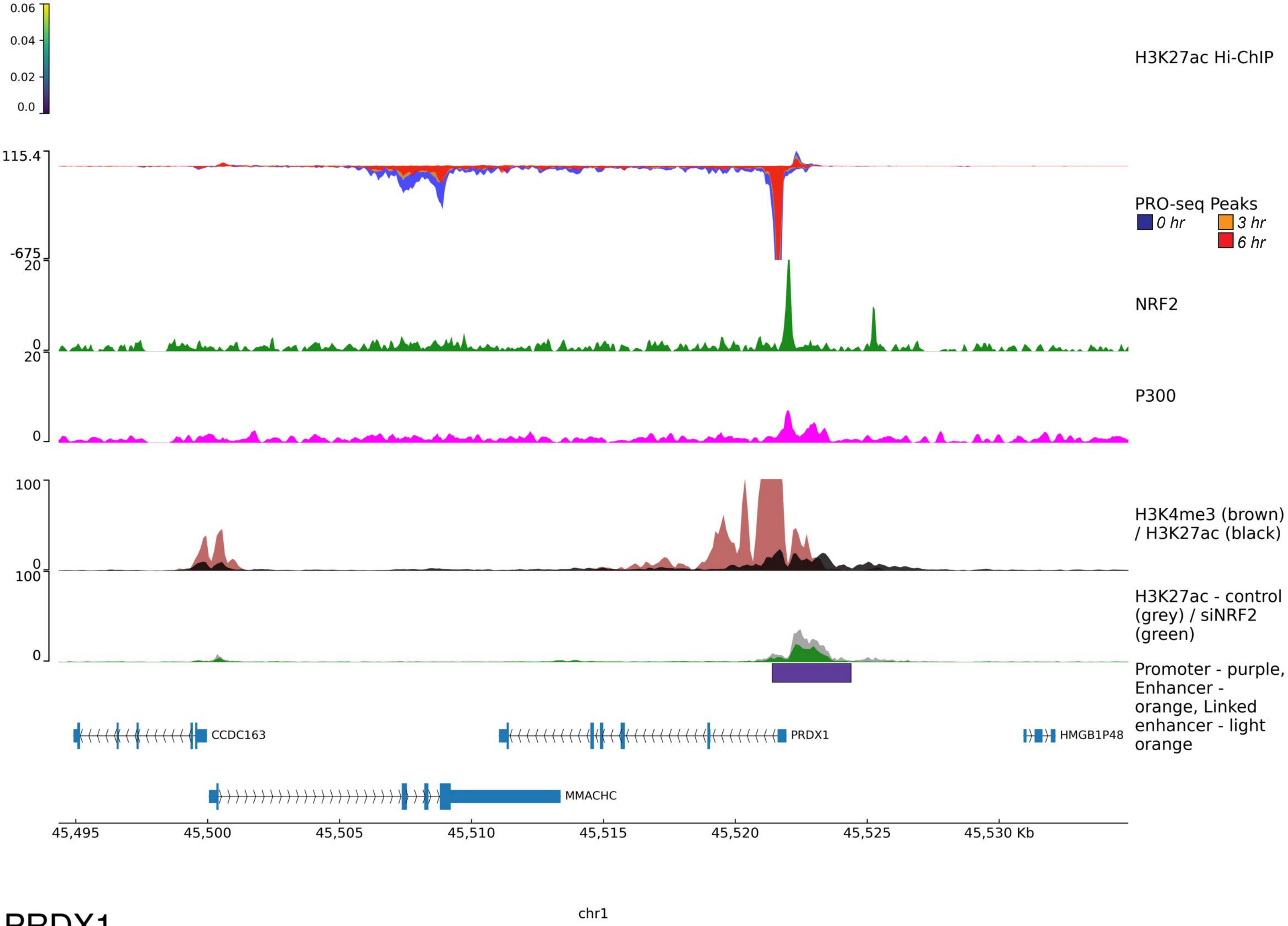

0.06
0.04
0.02
0.0
H3K27ac Hi-ChIP
115.4
-675
PRO-seq Peaks
0 hr
3 hr
6 hr
20
0
NRF2
20
0
P300
100
0
H3K4me3 (brown) / H3K27ac (black)
100
0
H3K27ac - control (grey) / siNRF2 (green)
Promoter - purple, Enhancer - orange, Linked enhancer - light orange
CCDC163
PRDX1
HMGB1P48
MMACHC
45,495
45,500
45,505
45,510
45,515
45,520
45,525
45,530 Kb
chr1

PRDX1

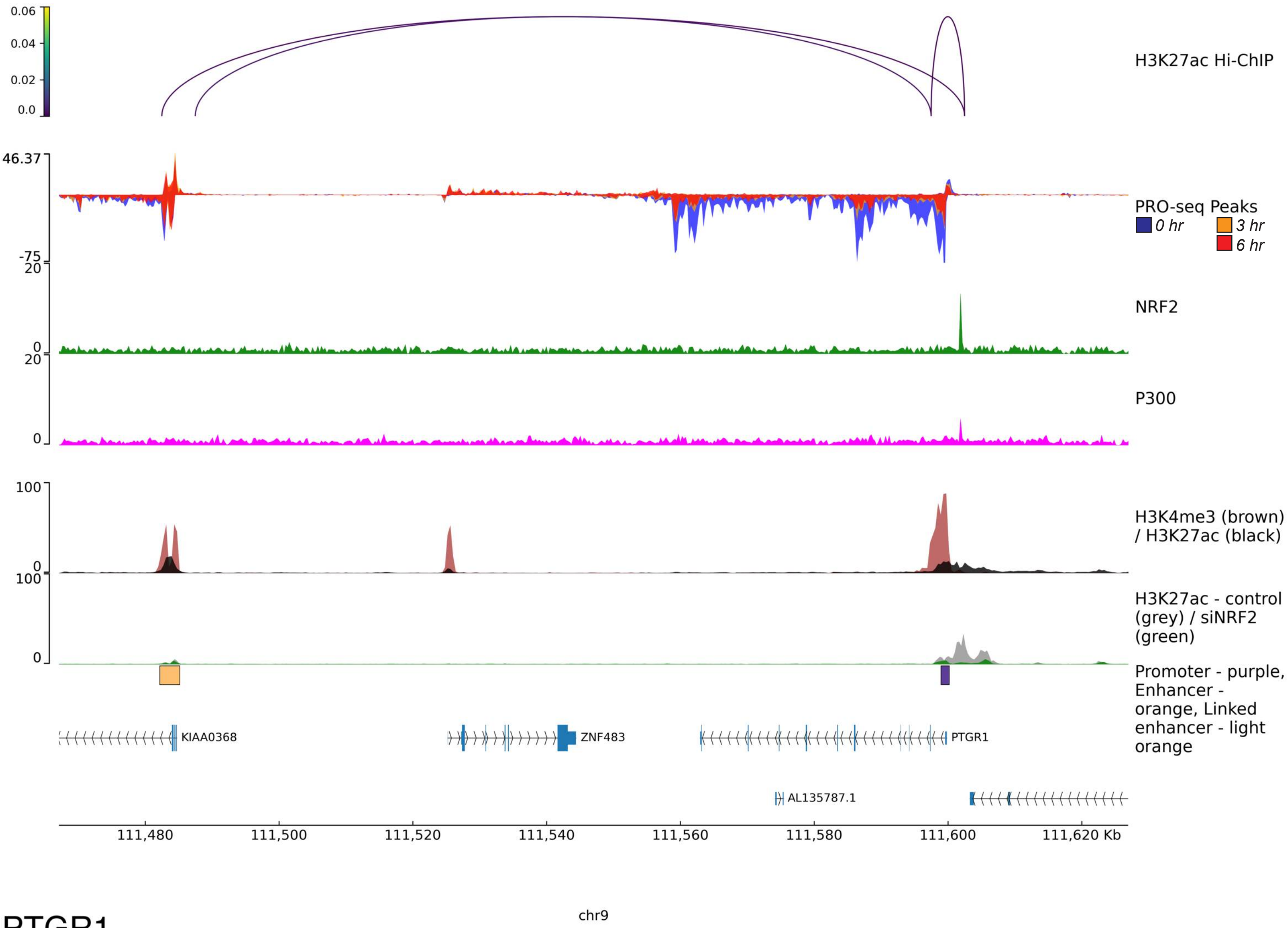

0.06
0.04
0.02
0.0
H3K27ac Hi-ChIP
46.37
-75
PRO-seq Peaks
0 hr
3 hr
6 hr
20
0
NRF2
20
0
P300
100
0
H3K4me3 (brown) / H3K27ac (black)
100
0
H3K27ac - control (grey) / siNRF2 (green)
Promoter - purple, Enhancer - orange, Linked enhancer - light orange
KIAA0368
ZNF483
PTGR1
AL135787.1
111,480
111,500
111,520
111,540
111,560
111,580
111,600
111,620 Kb
chr9

PTGR1

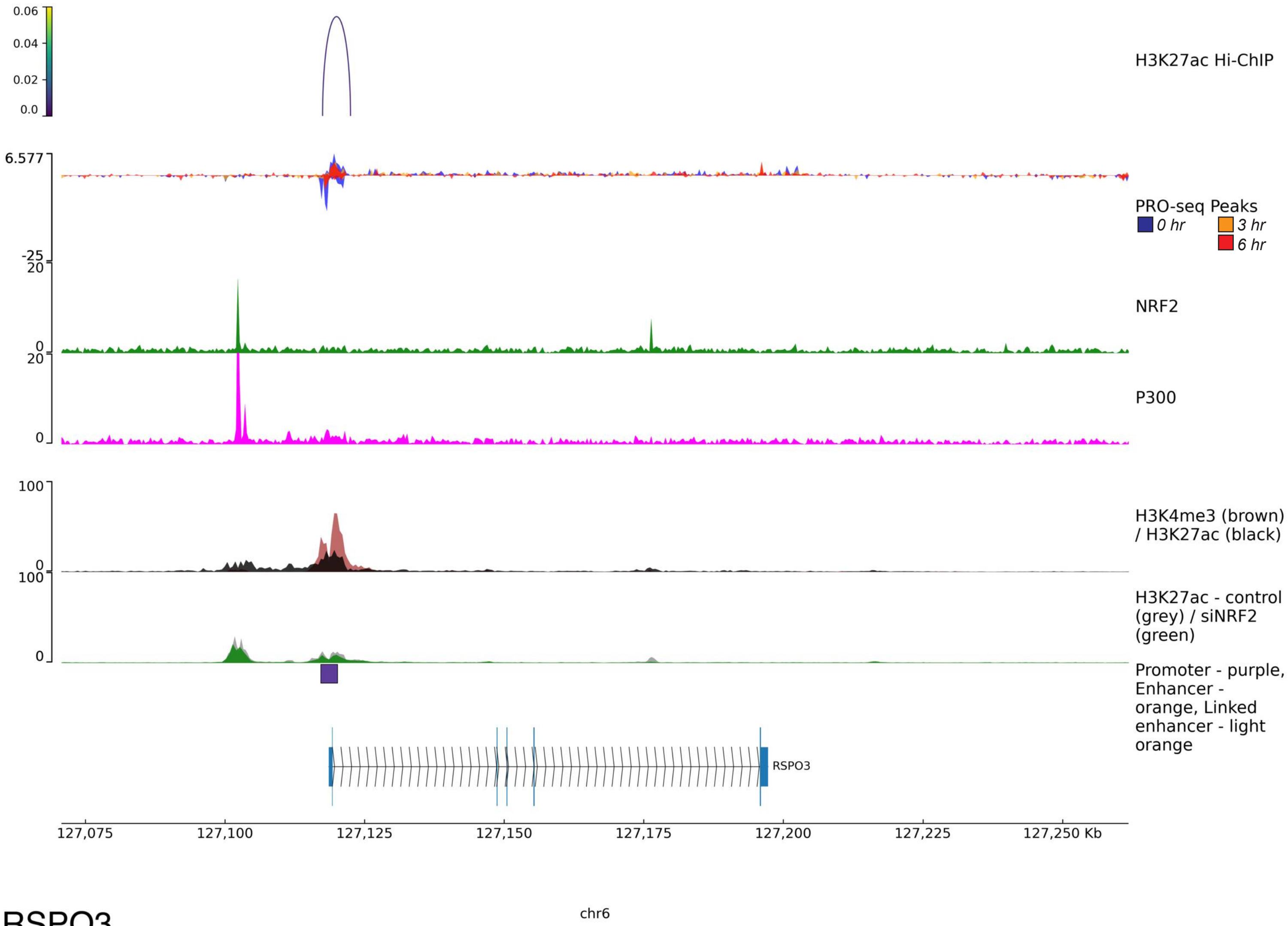
0.06
0.04
0.02
0.0
H3K27ac Hi-ChIP
6.577
-25
PRO-seq Peaks
0 hr
3 hr
6 hr
20
0
NRF2
20
0
P300
100
0
H3K4me3 (brown) / H3K27ac (black)
100
0
H3K27ac - control (grey) / siNRF2 (green)
Promoter - purple, Enhancer - orange, Linked enhancer - light orange
RSPO3
127,075
127,100
127,125
127,150
127,175
127,200
127,225
127,250 Kb
chr6

RSPO3

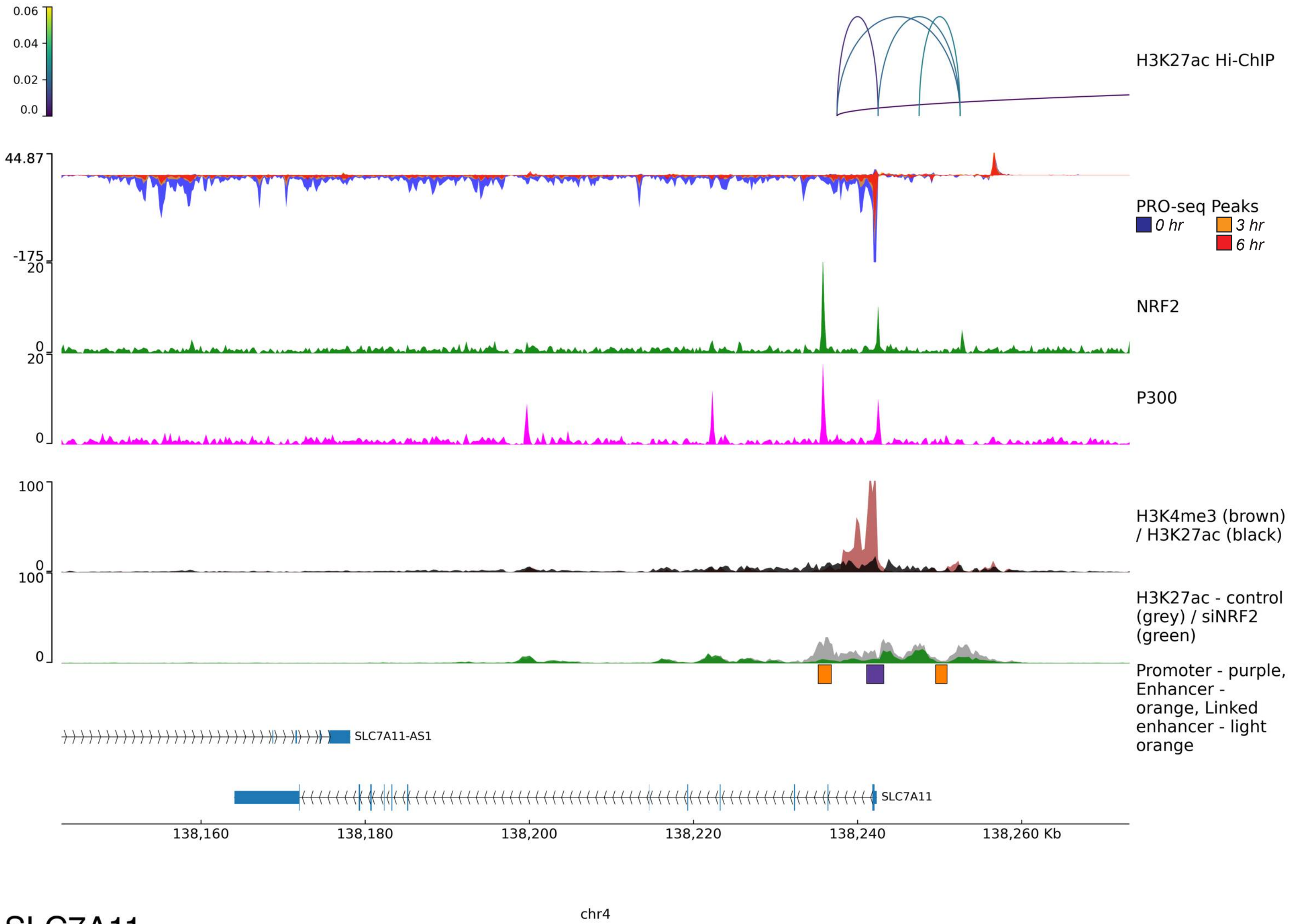

0.06
0.04
0.02
0.0
H3K27ac Hi-ChIP
44.87
-175
PRO-seq Peaks
0 hr
3 hr
6 hr
20
0
NRF2
20
0
P300
100
0
H3K4me3 (brown) / H3K27ac (black)
100
0
H3K27ac - control (grey) / siNRF2 (green)
Promoter - purple, Enhancer - orange, Linked enhancer - light orange
SLC7A11-AS1
SLC7A11
138,160
138,180
138,200
138,220
138,240
138,260 Kb
chr4

SLC7A11

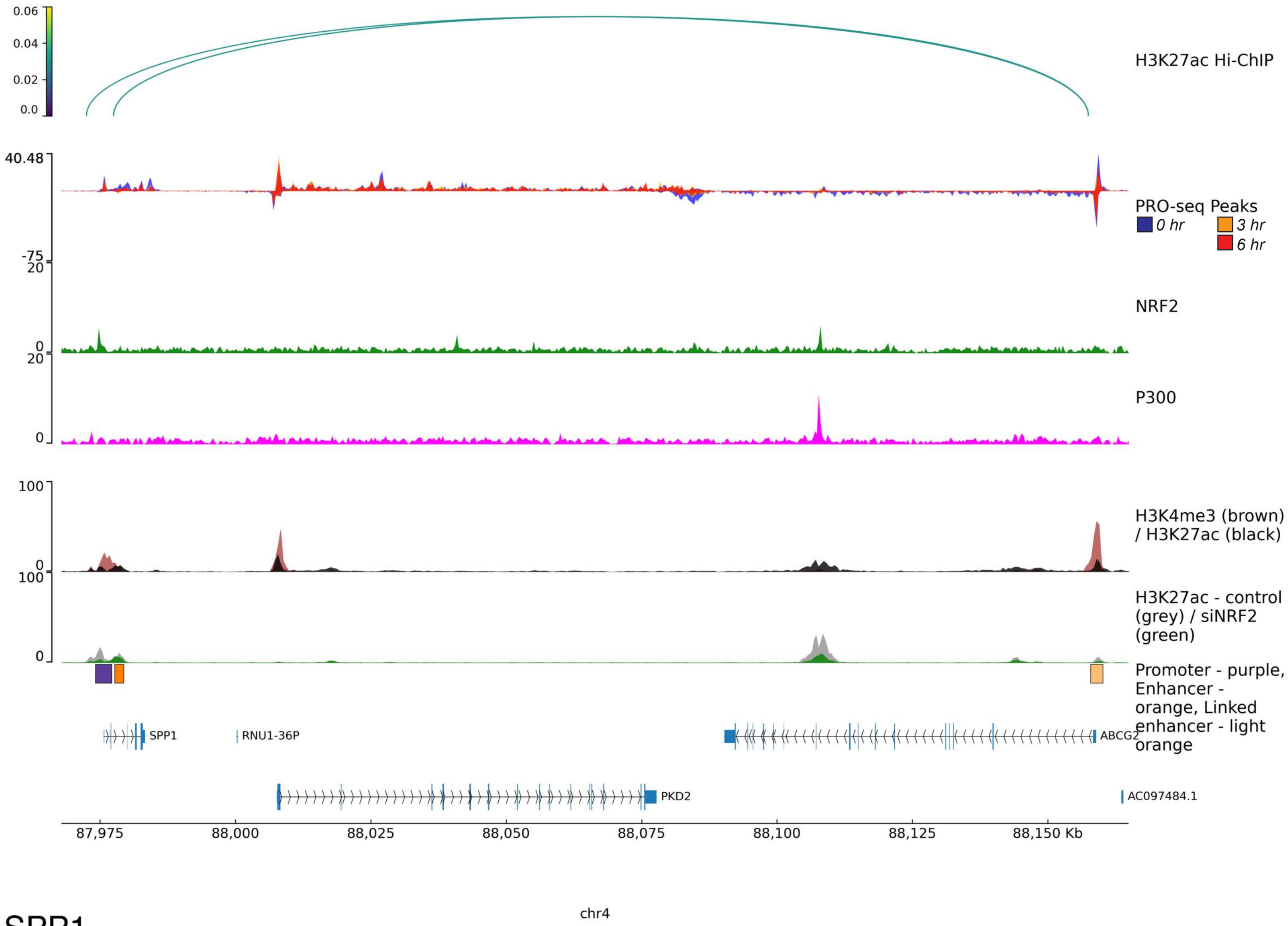
0.06
0.04
0.02
0.0
H3K27ac Hi-ChIP
40.48
-75
PRO-seq Peaks
0 hr
3 hr
6 hr
20
0
NRF2
20
0
P300
100
0
H3K4me3 (brown) / H3K27ac (black)
100
0
H3K27ac - control (grey) / siNRF2 (green)
Promoter - purple, Enhancer - orange, Linked enhancer - light orange
SPP1
RNU1-36P
ABCG2
PKD2
AC097484.1
87,975
88,000
88,025
88,050
88,075
88,100
88,125
88,150 Kb
chr4

SPP1

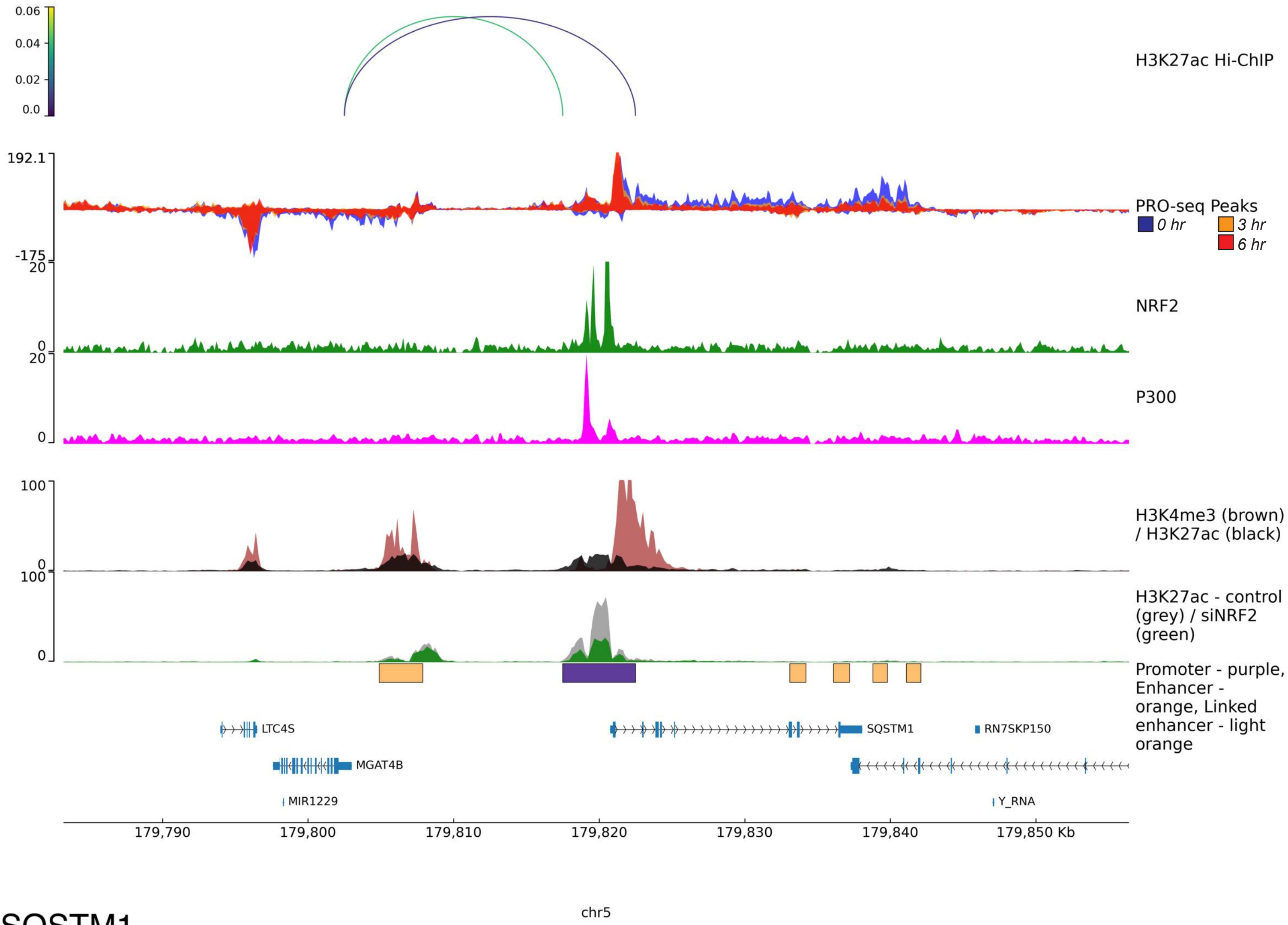
0.06
0.04
0.02
0.0
H3K27ac Hi-ChIP
192.1
-175
PRO-seq Peaks
0 hr
3 hr
6 hr
20
0
NRF2
20
0
P300
100
0
H3K4me3 (brown) / H3K27ac (black)
100
0
H3K27ac - control (grey) / siNRF2 (green)
Promoter - purple, Enhancer - orange, Linked enhancer - light orange
LTC4S
SQSTM1
RN7SKP150
MGAT4B
MIR1229
Y_RNA
179,790
179,800
179,810
179,820
179,830
179,840
179,850 Kb
chr5

SQSTM1

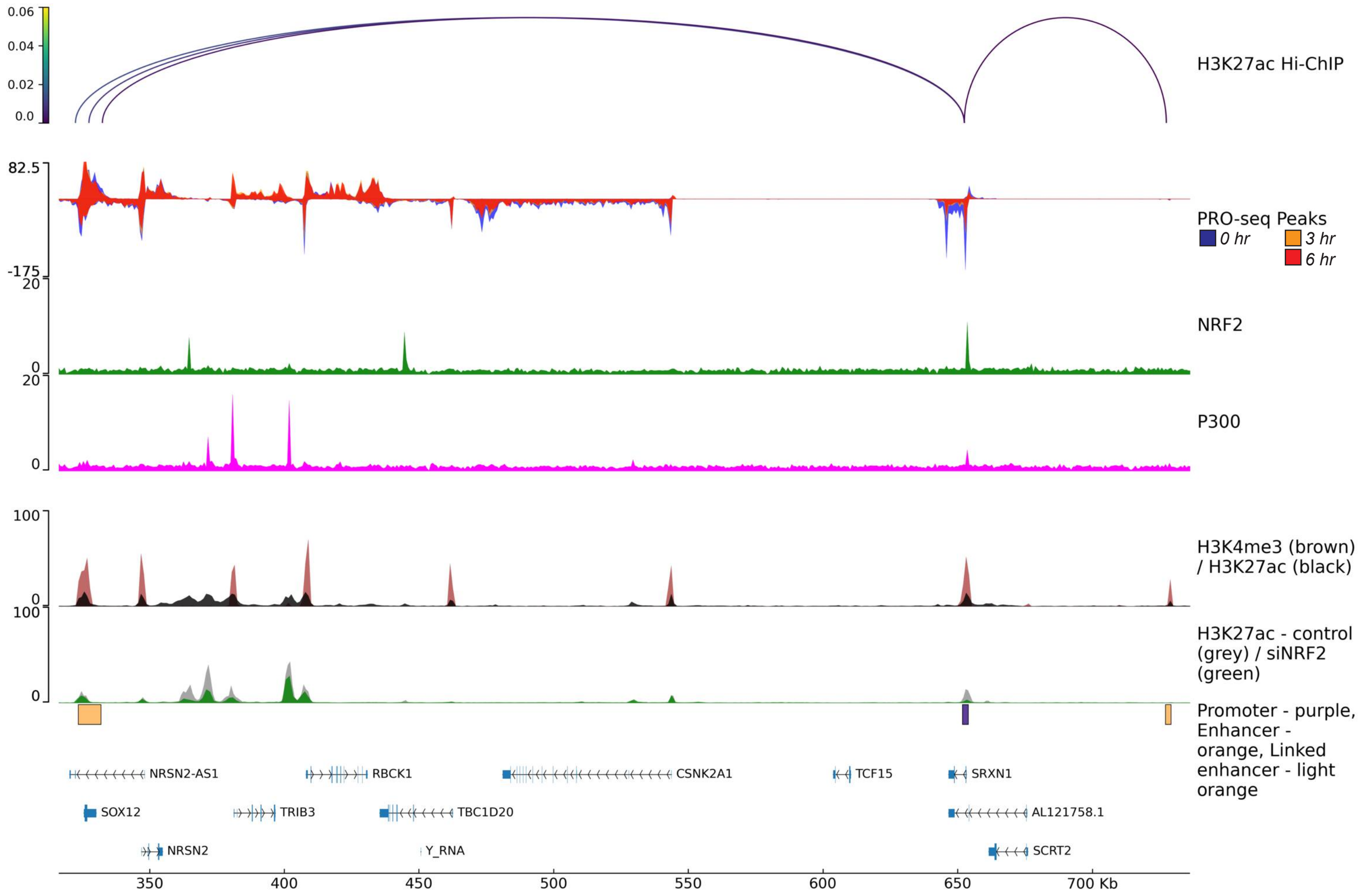

0.06
0.04
0.02
0.0
H3K27ac Hi-ChIP
82.5
-175
PRO-seq Peaks
0 hr
3 hr
6 hr
20
0
NRF2
20
0
P300
100
0
H3K4me3 (brown) / H3K27ac (black)
100
0
H3K27ac - control (grey) / siNRF2 (green)
Promoter - purple, Enhancer - orange, Linked enhancer - light orange
NRSN2-AS1
RBCK1
CSNK2A1
TCF15
SRXN1
SOX12
TRIB3
TBC1D20
AL121758.1
NRSN2
Y_RNA
SCRT2
350
400
450
500
550
600
650
700 Kb

chr20

SRXN1

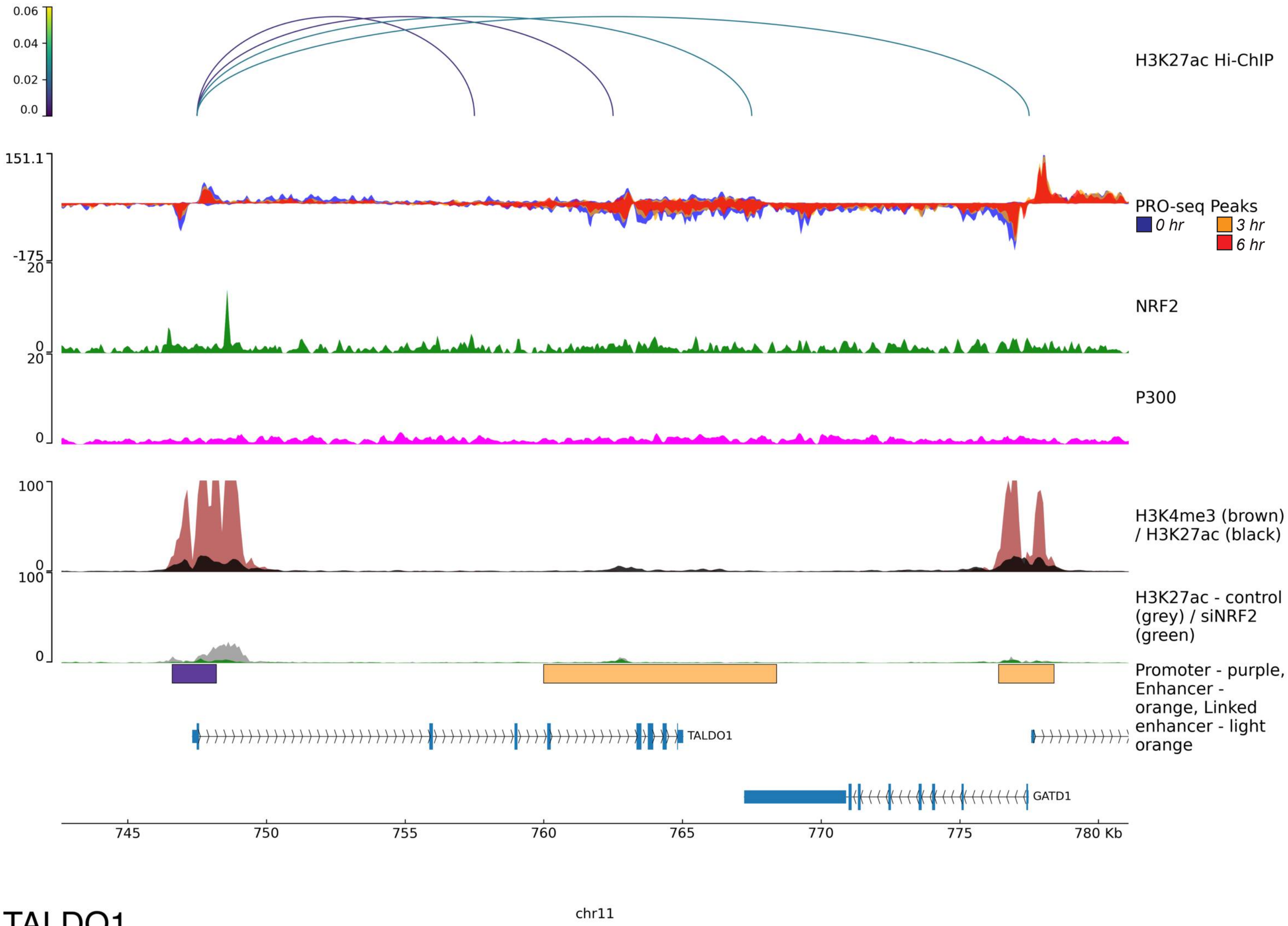
0.06
0.04
0.02
0.0
H3K27ac Hi-ChIP
151.1
-175
PRO-seq Peaks
0 hr
3 hr
6 hr
20
0
NRF2
20
0
P300
100
0
H3K4me3 (brown) / H3K27ac (black)
100
0
H3K27ac - control (grey) / siNRF2 (green)
Promoter - purple, Enhancer - orange, Linked enhancer - light orange
TALDO1
GATD1
745
750
755
760
765
770
775
780 Kb
chr11

TALDO1

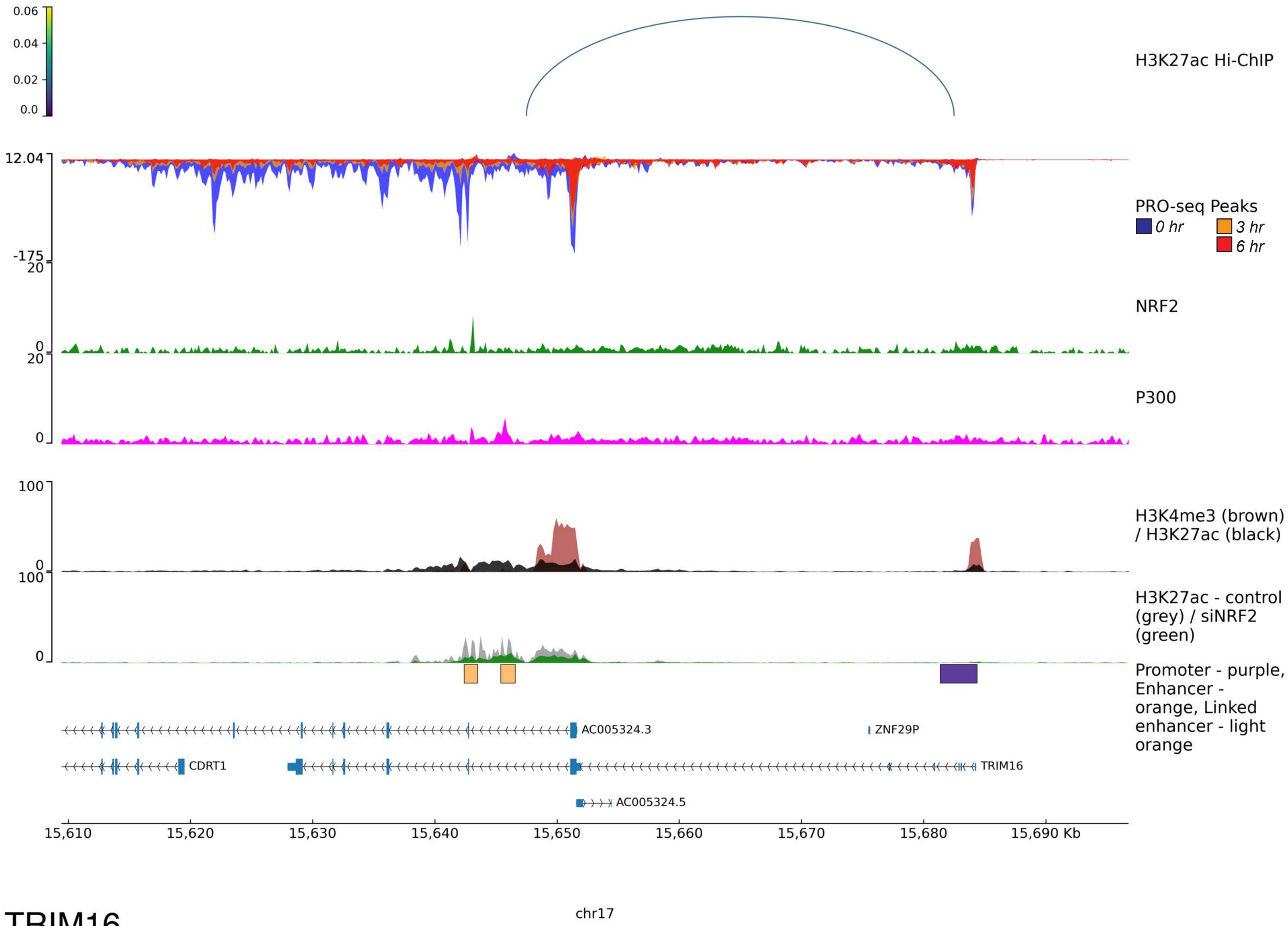

0.06
0.04
0.02
0.0
H3K27ac Hi-ChIP
12.04
-175
PRO-seq Peaks
0 hr
3 hr
6 hr
20
0
NRF2
20
0
P300
100
0
H3K4me3 (brown) / H3K27ac (black)
100
0
H3K27ac - control (grey) / siNRF2 (green)
Promoter - purple, Enhancer - orange, Linked enhancer - light orange
AC005324.3
ZNF29P
CDRT1
TRIM16
AC005324.5
15,610
15,620
15,630
15,640
15,650
15,660
15,670
15,680
15,690 Kb
chr17

TRIM16

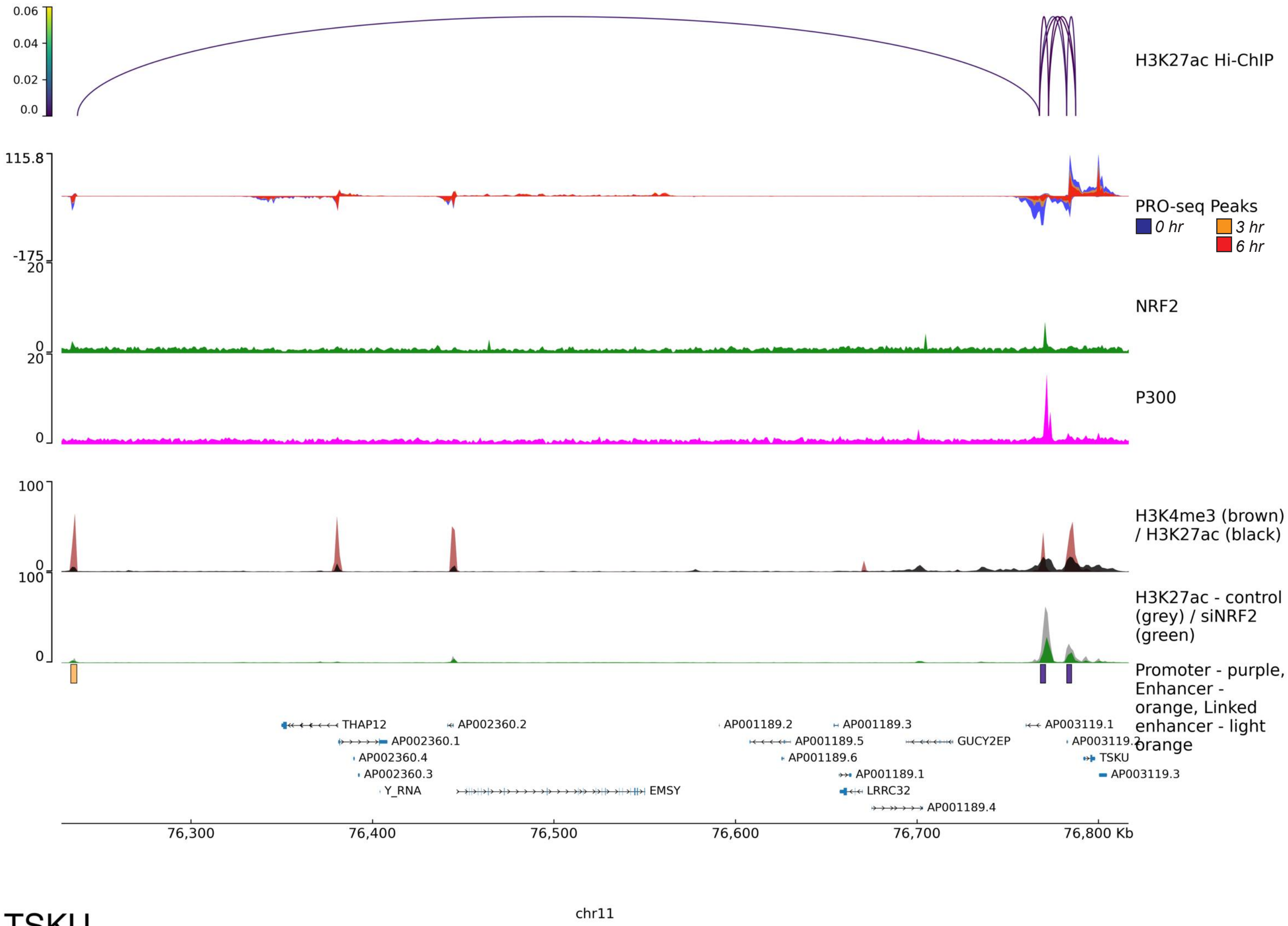

H3K27ac Hi-ChIP
0.06
0.04
0.02
0.0
PRO-seq Peaks
0 hr
3 hr
6 hr
115.8
-175
NRF2
20
0
P300
20
0
H3K4me3 (brown) / H3K27ac (black)
100
0
H3K27ac - control (grey) / siNRF2 (green)
100
0
Promoter - purple, Enhancer - orange, Linked enhancer - light orange
THAP12
AP002360.2
AP002360.1
AP002360.4
AP002360.3
Y_RNA
EMSY
AP001189.2
AP001189.3
AP001189.5
AP001189.6
AP001189.1
LRRC32
AP001189.4
GUCY2EP
AP003119.1
AP003119.2
TSKU
AP003119.3
76,300
76,400
76,500
76,600
76,700
76,800 Kb
chr11

TSKU

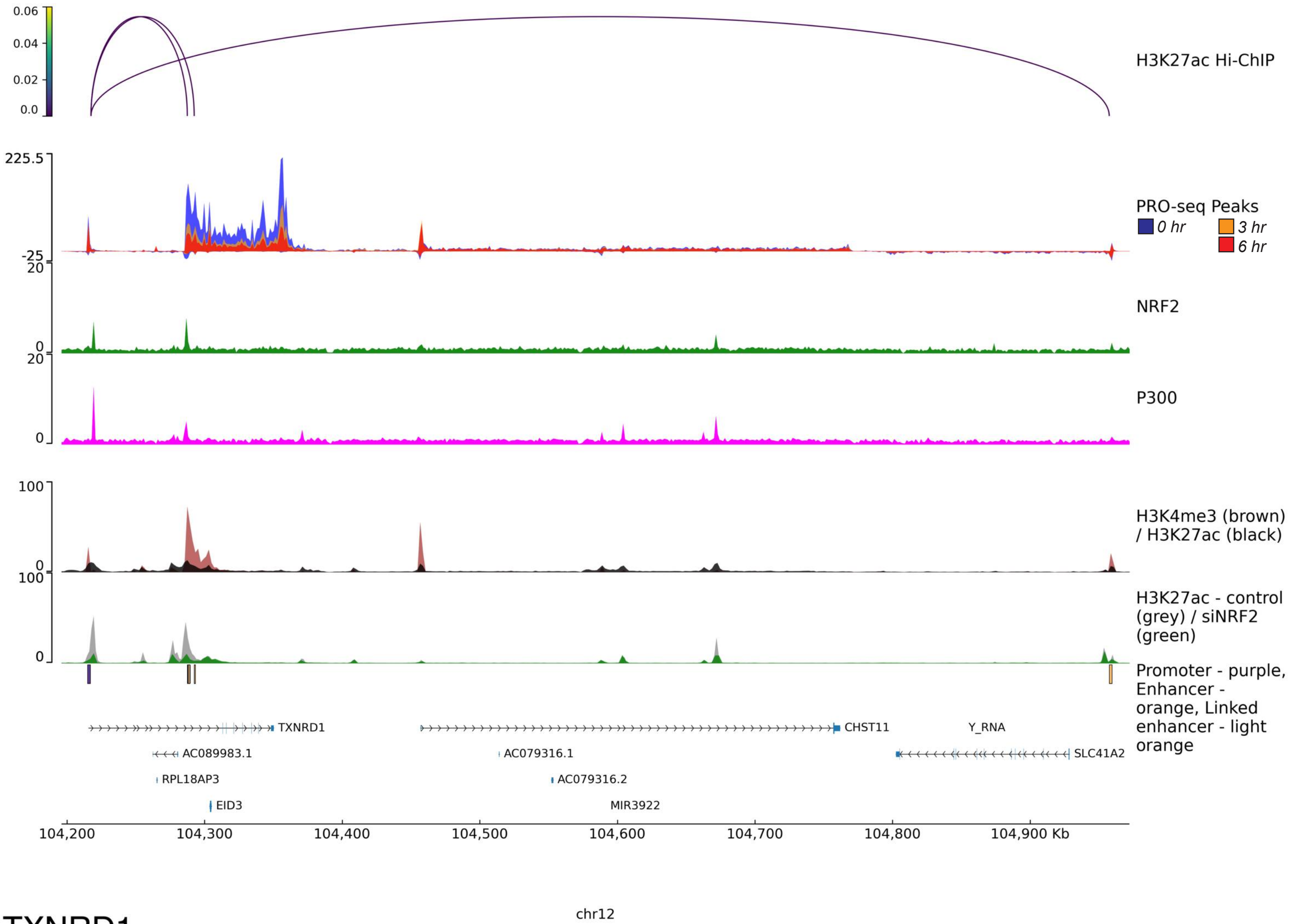
H3K27ac Hi-ChIP
PRO-seq Peaks
0 hr
3 hr
6 hr
NRF2
P300
H3K4me3 (brown) / H3K27ac (black)
H3K27ac - control (grey) / siNRF2 (green)
Promoter - purple, Enhancer - orange, Linked enhancer - light orange
TXNRD1
AC089983.1
RPL18AP3
EID3
CHST11
AC079316.1
AC079316.2
MIR3922
Y_RNA
SLC41A2
104,200
104,300
104,400
104,500
104,600
104,700
104,800
104,900 Kb
chr12

TXNRD1

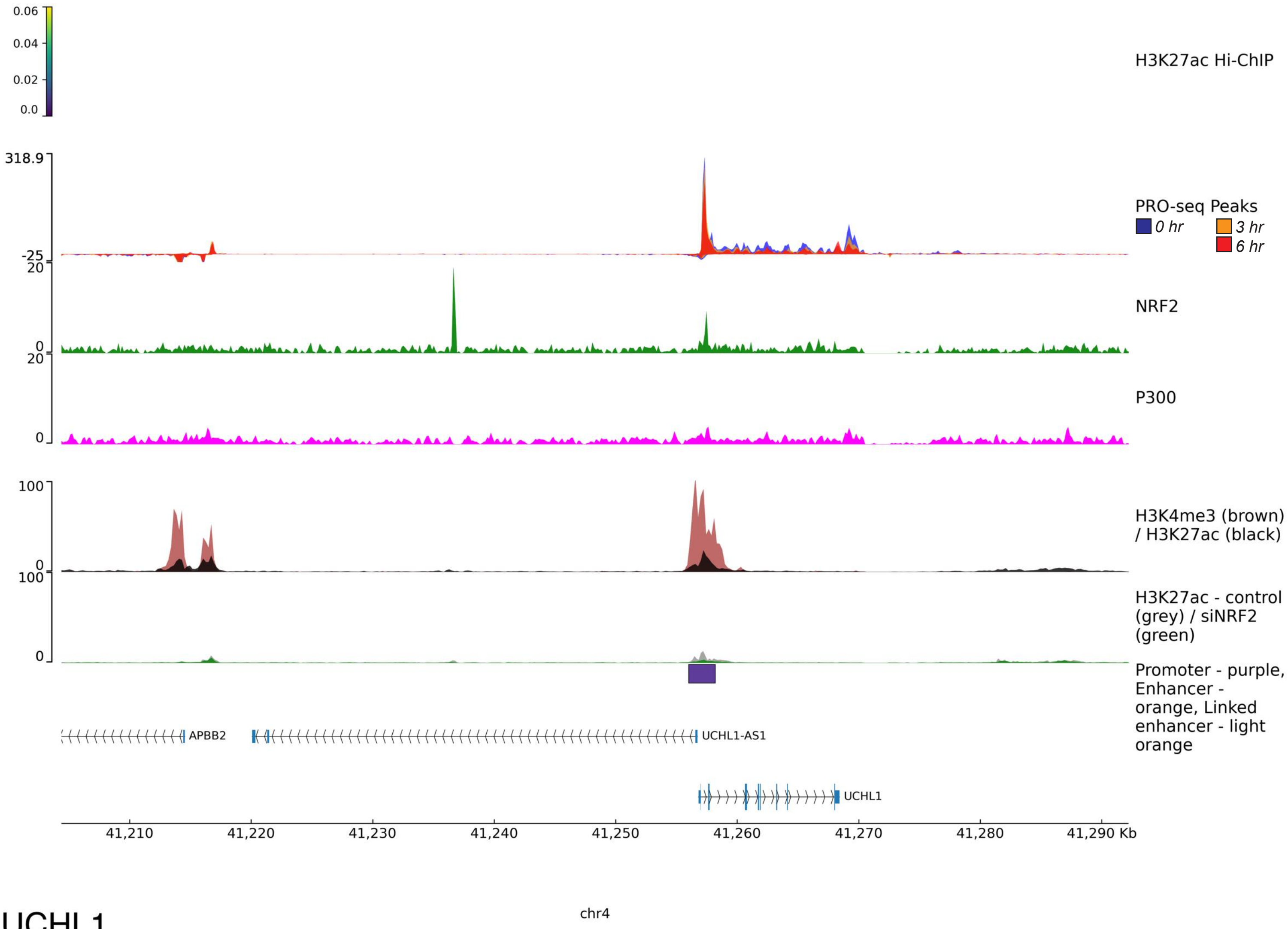
0.06
0.04
0.02
0.0
H3K27ac Hi-ChIP
318.9
-25
PRO-seq Peaks
0 hr
3 hr
6 hr
20
0
NRF2
20
0
P300
100
0
H3K4me3 (brown) / H3K27ac (black)
100
0
H3K27ac - control (grey) / siNRF2 (green)
Promoter - purple, Enhancer - orange, Linked enhancer - light orange
APBB2
UCHL1-AS1
UCHL1
41,210
41,220
41,230
41,240
41,250
41,260
41,270
41,280
41,290 Kb
chr4

UCHL1

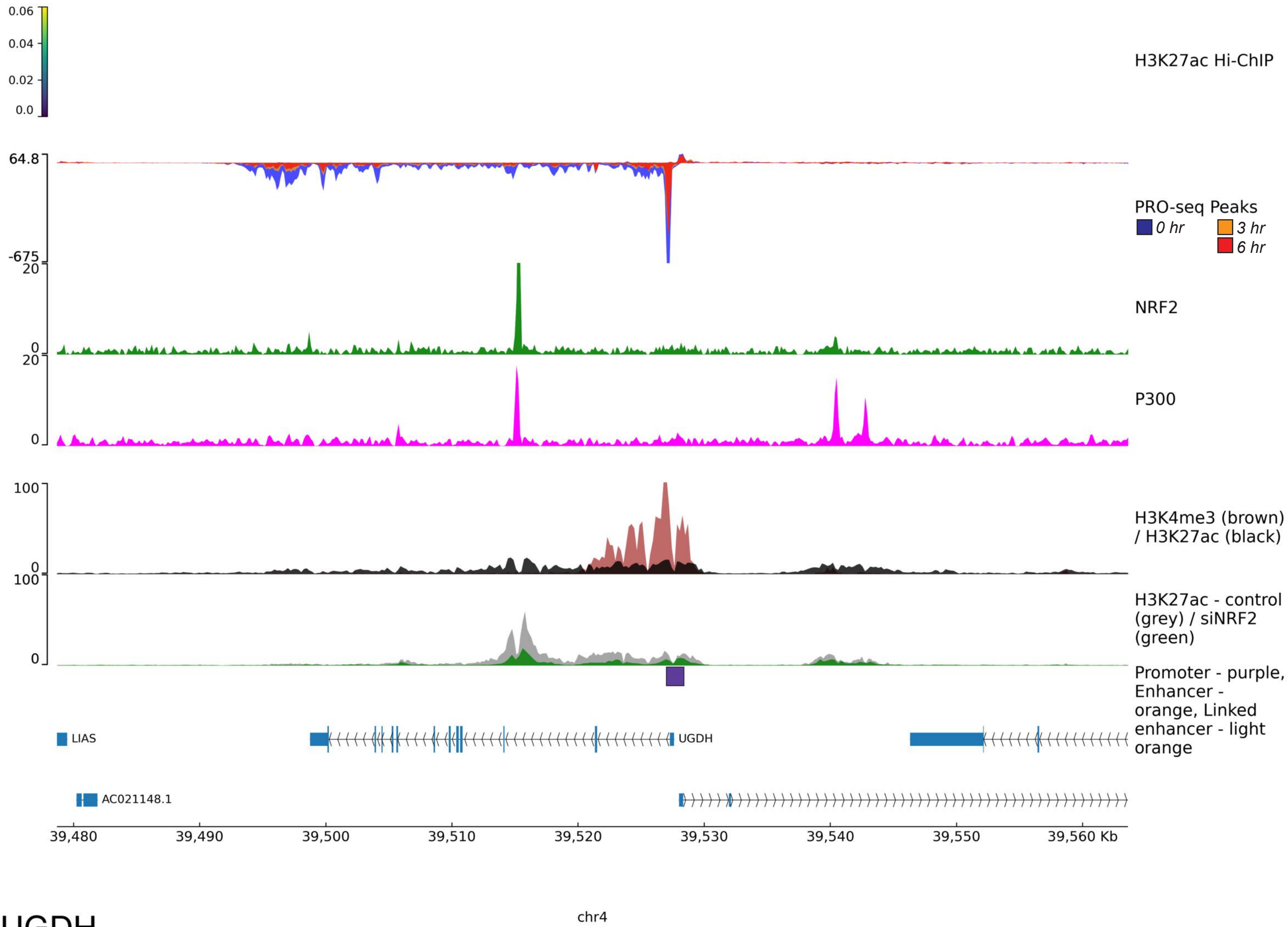

0.06
0.04
0.02
0.0
H3K27ac Hi-ChIP
64.8
-675
PRO-seq Peaks
0 hr
3 hr
6 hr
20
0
NRF2
20
0
P300
100
0
H3K4me3 (brown) / H3K27ac (black)
100
0
H3K27ac - control (grey) / siNRF2 (green)
Promoter - purple, Enhancer - orange, Linked enhancer - light orange
LIAS
UGDH
AC021148.1
39,480
39,490
39,500
39,510
39,520
39,530
39,540
39,550
39,560 Kb
chr4

UGDH

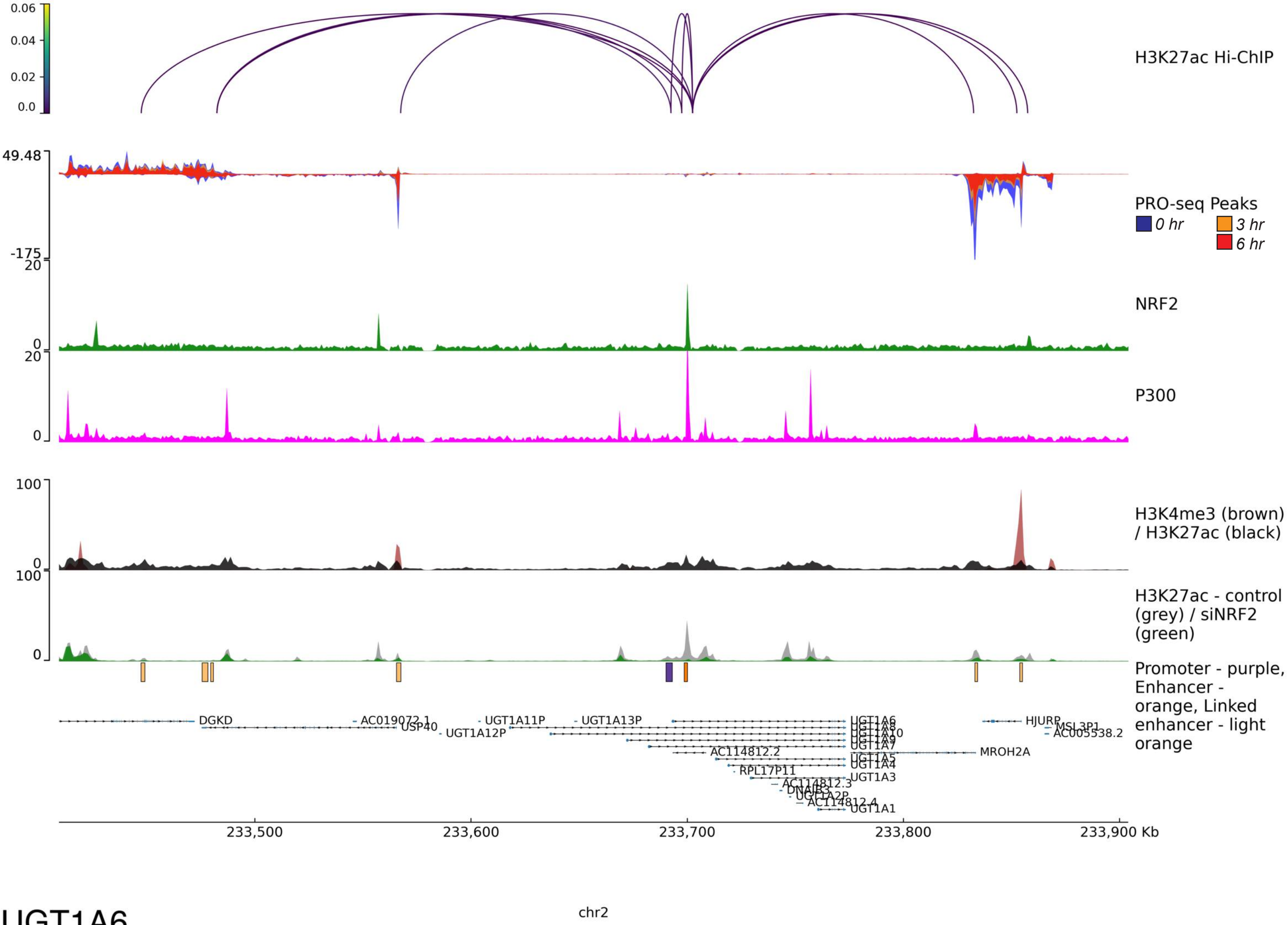

H3K27ac Hi-ChIP
0.06
0.04
0.02
0.0
49.48
-175
PRO-seq Peaks
0 hr
3 hr
6 hr
20
0
NRF2
20
0
P300
100
0
H3K4me3 (brown) / H3K27ac (black)
100
0
H3K27ac - control (grey) / siNRF2 (green)
Promoter - purple, Enhancer - orange, Linked enhancer - light orange
DGKD
AC019072.1
USP40
UGT1A11P
UGT1A12P
UGT1A13P
UGT1A6
UGT1A8
UGT1A10
UGT1A9
UGT1A7
AC114812.2
UGT1A5
UGT1A4
RPL17P11
UGT1A3
AC114812.3
DNAJB3
UGT1A2P
AC114812.4
UGT1A1
MROH2A
HJURP
MSL3P1
AC005538.2
233,500
233,600
233,700
233,800
233,900 Kb
chr2

UGT1A6
