# Supplementary figures and images for: NRF2 supports non-small cell lung cancer growth independently of CBP/p300-enhanced glutathione synthesis
Source: EMBO Rep. 2025 May 14;26(12):3106–37. doi: 10.1038/s44319-025-00463-z (PMC12187939; doi:10.1038/s44319-025-00463-z)

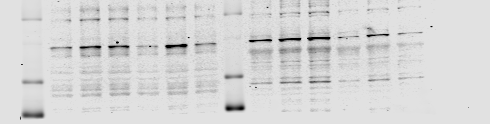

Supplement: Supplementary file 13 — Source data Fig. 3 [file 44319_2025_463_MOESM13_ESM.zip › Figure 3/Figure_3G/3.9.23_A549sh_BIND_rescue_48_dox_rep2_3_GCLC.tif]

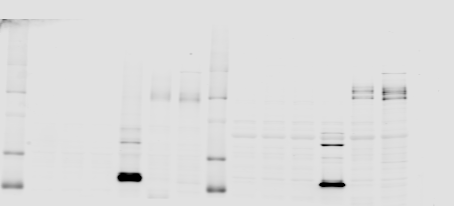

Supplement: Supplementary file 13 — Source data Fig. 3 [file 44319_2025_463_MOESM13_ESM.zip › Figure 3/Figure_3G/3.9.23_A549sh_BIND_rescue_48_dox_rep2_3_FLAG.tif]

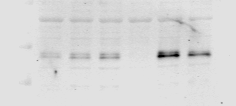

Supplement: Supplementary file 13 — Source data Fig. 3 [file 44319_2025_463_MOESM13_ESM.zip › Figure 3/Figure_3G/3.14.23_A549sh_BIND_rescue_48_dox_rep2_3_DAX1.tif]

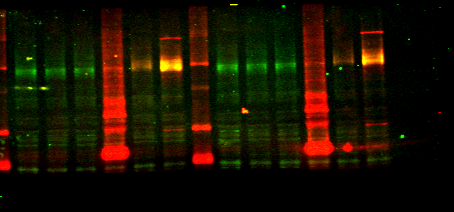

Supplement: Supplementary file 13 — Source data Fig. 3 [file 44319_2025_463_MOESM13_ESM.zip › Figure 3/Figure_3G/3.15.23_A549sh_BIND_rescue_48_dox_rep4_5_overlay.tif]

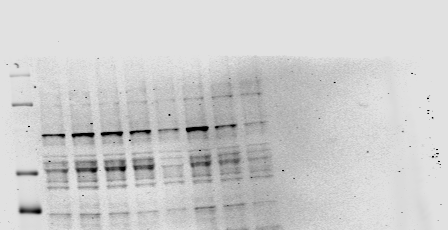

Supplement: Supplementary file 13 — Source data Fig. 3 [file 44319_2025_463_MOESM13_ESM.zip › Figure 3/Figure_3G/12.9.22_A549sh_BIND_Neh45mut_48h_GCLC_G6PD.tif]

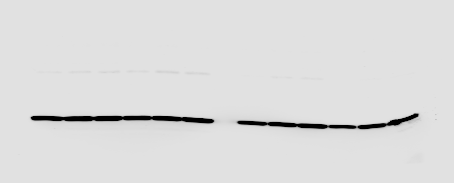

Supplement: Supplementary file 13 — Source data Fig. 3 [file 44319_2025_463_MOESM13_ESM.zip › Figure 3/Figure_3G/3.9.23_A549sh_BIND_rescue_48_dox_rep2_3_H2B.tif]

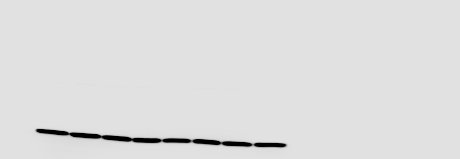

Supplement: Supplementary file 13 — Source data Fig. 3 [file 44319_2025_463_MOESM13_ESM.zip › Figure 3/Figure_3G/12.9.22_A549sh_BIND_Neh45mut_48h_H2B.tif]

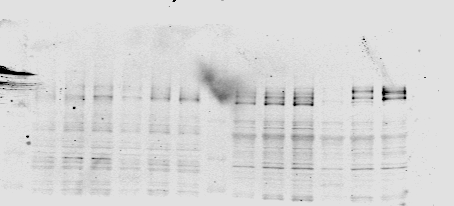

Supplement: Supplementary file 13 — Source data Fig. 3 [file 44319_2025_463_MOESM13_ESM.zip › Figure 3/Figure_3G/3.9.23_A549sh_BIND_rescue_48_dox_rep2_3_NRF2.tif]

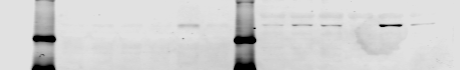

Supplement: Supplementary file 13 — Source data Fig. 3 [file 44319_2025_463_MOESM13_ESM.zip › Figure 3/Figure_3G/3.9.23_A549sh_BIND_rescue_48_dox_rep2_3_SQSTM1.tif]

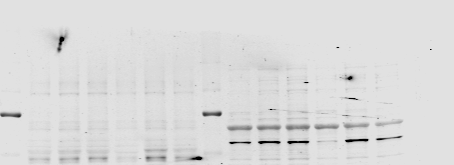

Supplement: Supplementary file 13 — Source data Fig. 3 [file 44319_2025_463_MOESM13_ESM.zip › Figure 3/Figure_3G/3.9.23_A549sh_BIND_rescue_48_dox_rep2_3_TXNRD1.tif]

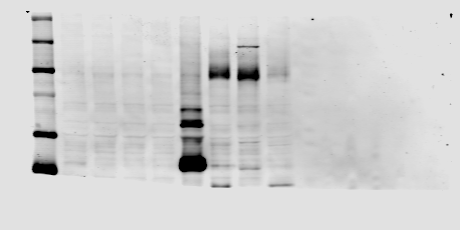

Supplement: Supplementary file 13 — Source data Fig. 3 [file 44319_2025_463_MOESM13_ESM.zip › Figure 3/Figure_3G/12.9.22_A549sh_BIND_Neh45mut_48h_FLAG.tif]

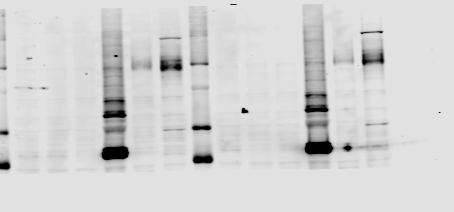

Supplement: Supplementary file 13 — Source data Fig. 3 [file 44319_2025_463_MOESM13_ESM.zip › Figure 3/Figure_3G/3.15.23_A549sh_BIND_rescue_48_dox_rep4_5_FLAG.tif]

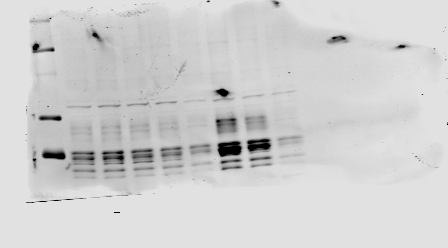

Supplement: Supplementary file 13 — Source data Fig. 3 [file 44319_2025_463_MOESM13_ESM.zip › Figure 3/Figure_3G/12.9.22_A549sh_BIND_Neh45mut_48h_TALDO1_DAX1.tif]

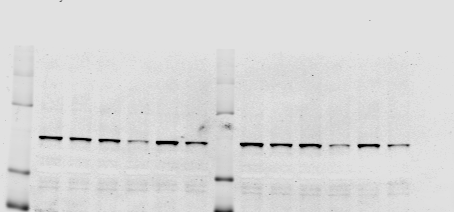

Supplement: Supplementary file 13 — Source data Fig. 3 [file 44319_2025_463_MOESM13_ESM.zip › Figure 3/Figure_3G/3.15.23_A549sh_BIND_rescue_48_dox_rep4_5_GCLC.tif]

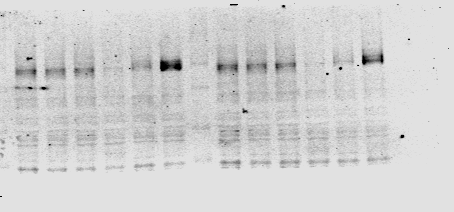

Supplement: Supplementary file 13 — Source data Fig. 3 [file 44319_2025_463_MOESM13_ESM.zip › Figure 3/Figure_3G/3.15.23_A549sh_BIND_rescue_48_dox_rep4_5_NRF2.tif]

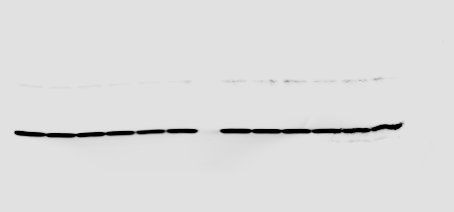

Supplement: Supplementary file 13 — Source data Fig. 3 [file 44319_2025_463_MOESM13_ESM.zip › Figure 3/Figure_3G/3.15.23_A549sh_BIND_rescue_48_dox_rep4_5_H2B.tif]

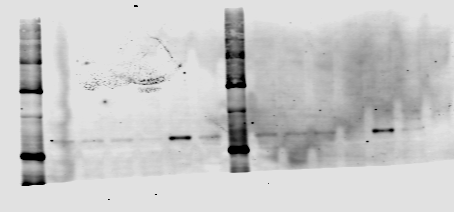

Supplement: Supplementary file 13 — Source data Fig. 3 [file 44319_2025_463_MOESM13_ESM.zip › Figure 3/Figure_3G/3.15.23_A549sh_BIND_rescue_48_dox_rep4_5_SQSTM1.tif]

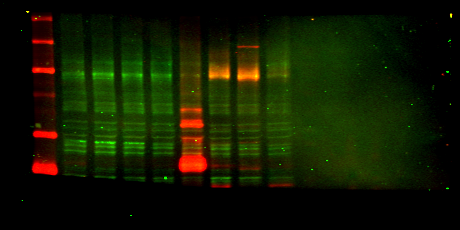

Supplement: Supplementary file 13 — Source data Fig. 3 [file 44319_2025_463_MOESM13_ESM.zip › Figure 3/Figure_3G/12.9.22_A549sh_BIND_Neh45mut_48h_overlay.tif]

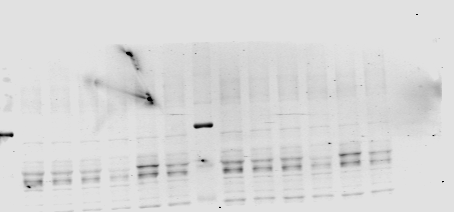

Supplement: Supplementary file 13 — Source data Fig. 3 [file 44319_2025_463_MOESM13_ESM.zip › Figure 3/Figure_3G/3.15.23_A549sh_BIND_rescue_48_dox_rep4_5_TXNRD1.tif]

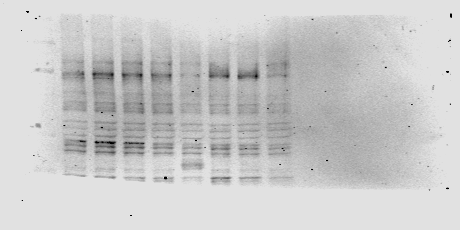

Supplement: Supplementary file 13 — Source data Fig. 3 [file 44319_2025_463_MOESM13_ESM.zip › Figure 3/Figure_3G/12.9.22_A549sh_BIND_Neh45mut_48h_NRF2.tif]

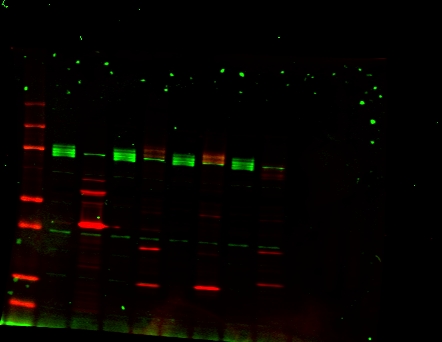

Supplement: Supplementary file 13 — Source data Fig. 3 [file 44319_2025_463_MOESM13_ESM.zip › Figure 3/Figure_3E/A549sh_BIND_rescue_rep1_overlay.jpg]

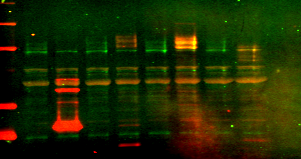

Supplement: Supplementary file 13 — Source data Fig. 3 [file 44319_2025_463_MOESM13_ESM.zip › Figure 3/Figure_3E/A549sh_BIND_dox_24h_rep2_overlay.tif]

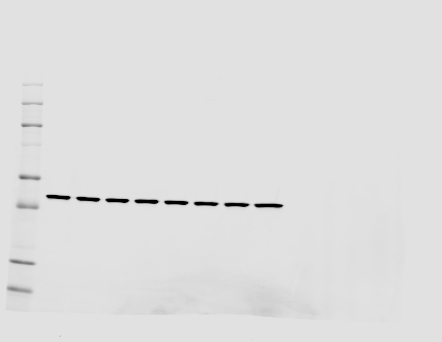

Supplement: Supplementary file 13 — Source data Fig. 3 [file 44319_2025_463_MOESM13_ESM.zip › Figure 3/Figure_3E/A549sh_BIND_rescue_rep1_actin.jpg]

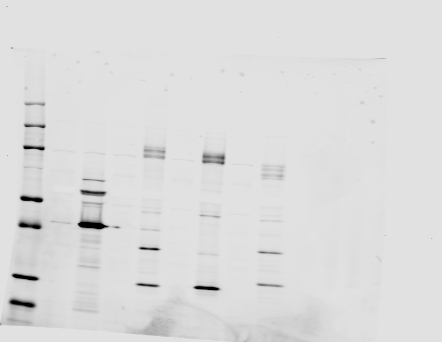

Supplement: Supplementary file 13 — Source data Fig. 3 [file 44319_2025_463_MOESM13_ESM.zip › Figure 3/Figure_3E/A549sh_BIND_rescue_rep1_FLAG.jpg]

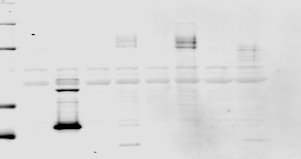

Supplement: Supplementary file 13 — Source data Fig. 3 [file 44319_2025_463_MOESM13_ESM.zip › Figure 3/Figure_3E/A549sh_BIND_dox_24h_rep2_FLAG.tif]

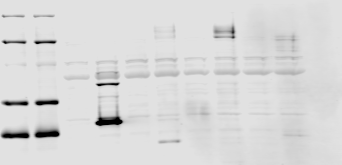

Supplement: Supplementary file 13 — Source data Fig. 3 [file 44319_2025_463_MOESM13_ESM.zip › Figure 3/Figure_3E/A549sh_BIND_dox_24h_rep3_FLAG.tif]

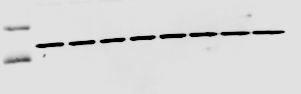

Supplement: Supplementary file 13 — Source data Fig. 3 [file 44319_2025_463_MOESM13_ESM.zip › Figure 3/Figure_3E/A549sh_BIND_dox_24h_rep2_actin.tif]

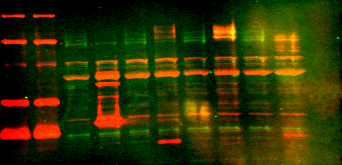

Supplement: Supplementary file 13 — Source data Fig. 3 [file 44319_2025_463_MOESM13_ESM.zip › Figure 3/Figure_3E/A549sh_BIND_dox_24h_rep3_overlay.tif]

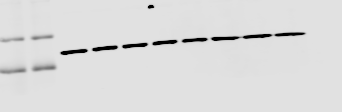

Supplement: Supplementary file 13 — Source data Fig. 3 [file 44319_2025_463_MOESM13_ESM.zip › Figure 3/Figure_3E/A549sh_BIND_dox_24h_rep3_actin.tif]

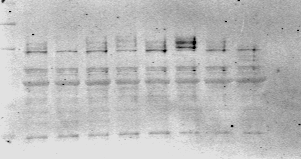

Supplement: Supplementary file 13 — Source data Fig. 3 [file 44319_2025_463_MOESM13_ESM.zip › Figure 3/Figure_3E/A549sh_BIND_dox_24h_rep2_NRF2.tif]

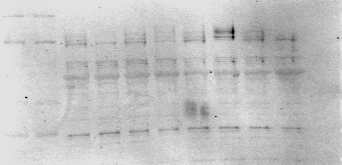

Supplement: Supplementary file 13 — Source data Fig. 3 [file 44319_2025_463_MOESM13_ESM.zip › Figure 3/Figure_3E/A549sh_BIND_dox_24h_rep3_NRF2.tif]

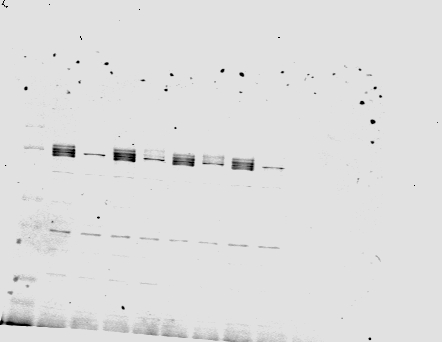

Supplement: Supplementary file 13 — Source data Fig. 3 [file 44319_2025_463_MOESM13_ESM.zip › Figure 3/Figure_3E/A549sh_BIND_rescue_rep1_NRF2.jpg]

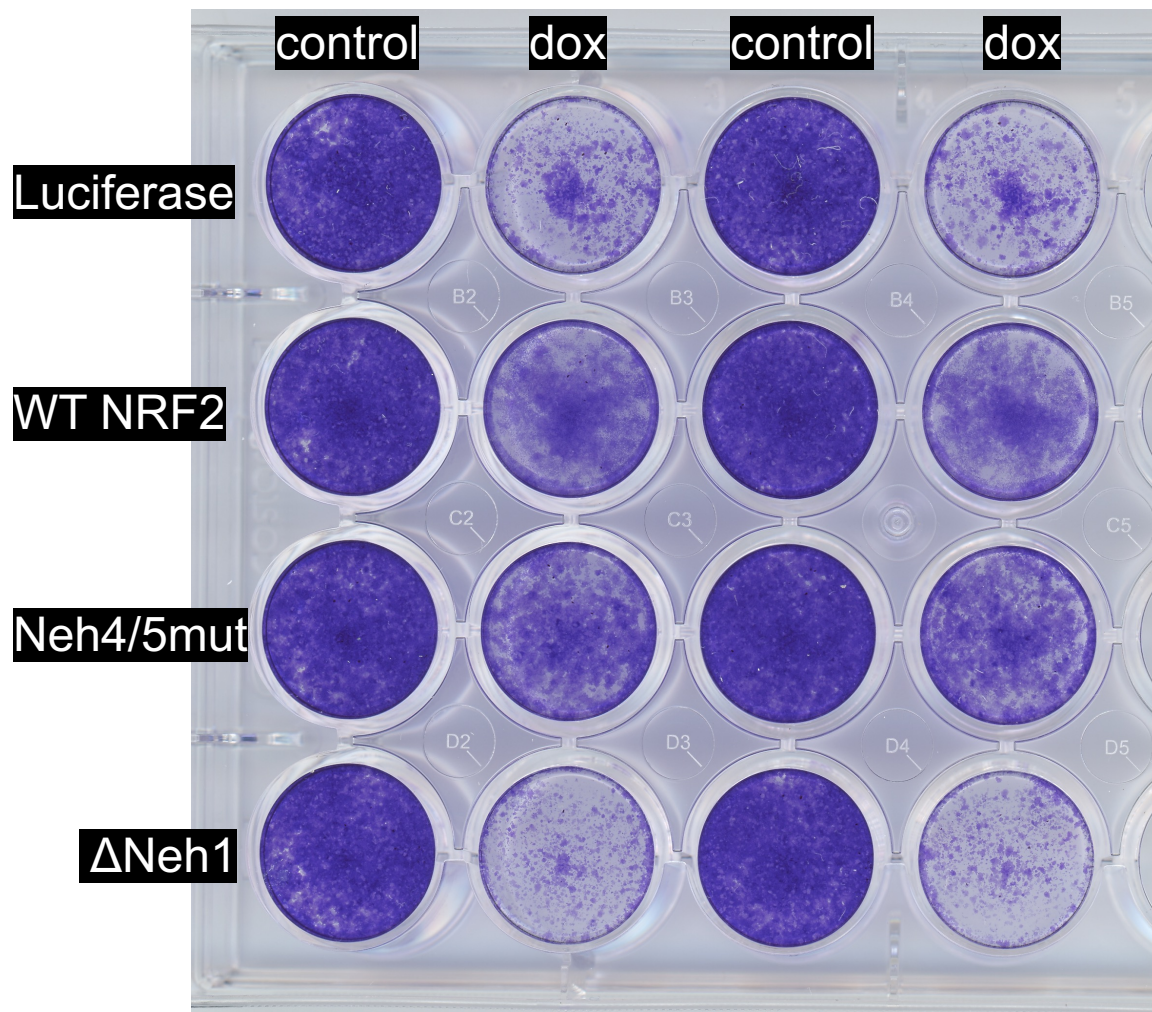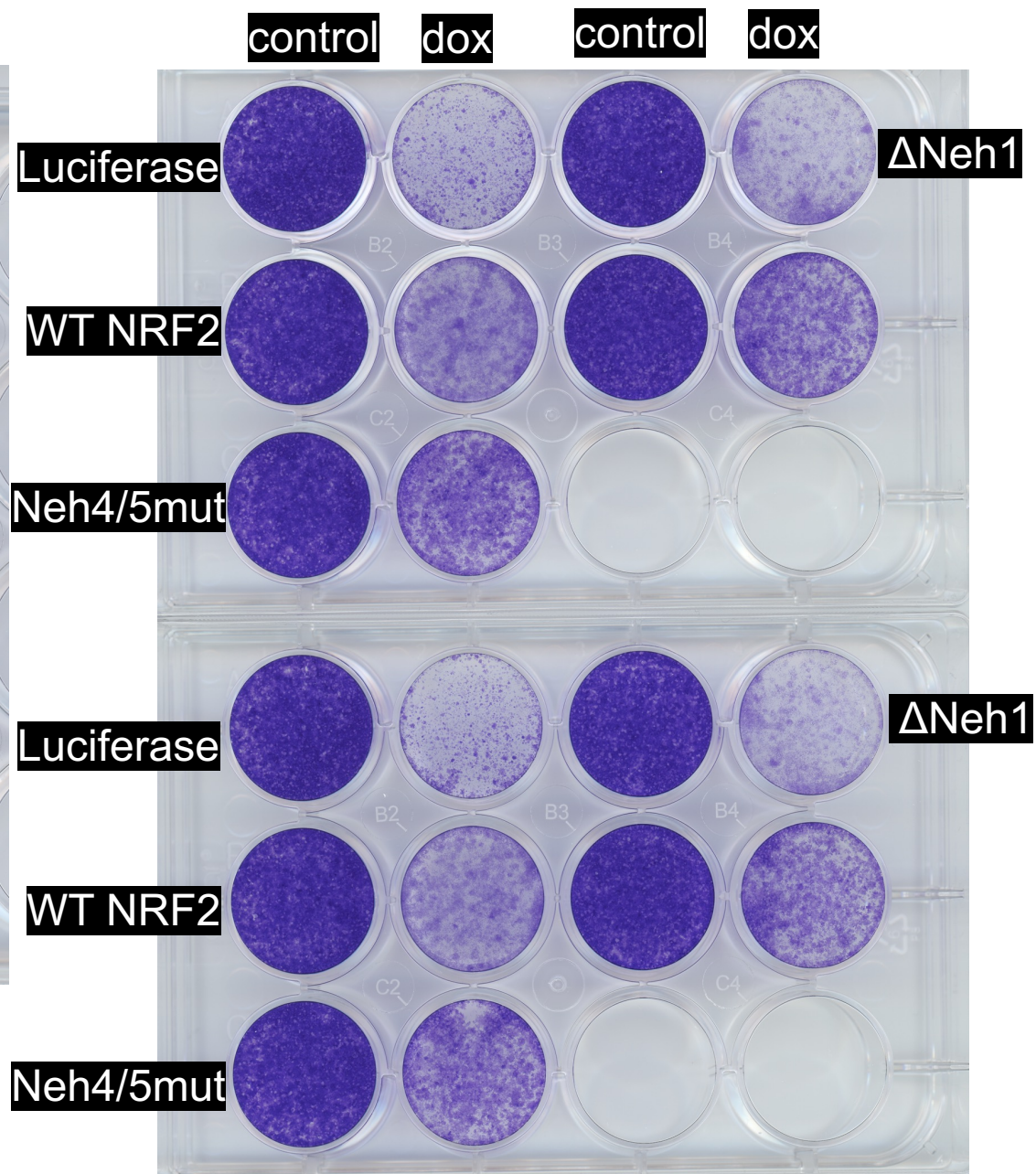

Supplement: Supplementary file 14 — Source data Fig. 4 [file 44319_2025_463_MOESM14_ESM.zip › Figure 4/Figure_4B.pdf]
