# Supplementary material for: NRF2 supports non-small cell lung cancer growth independently of CBP/p300-enhanced glutathione synthesis
Source: EMBO Rep. 2025 May 14;26(12):3106–37. doi: 10.1038/s44319-025-00463-z (PMC12187939; doi:10.1038/s44319-025-00463-z)
Supplement: Supplementary file 17 — Expanded View Figures [file 44319_2025_463_MOESM17_ESM.pdf]

Expanded View Figures

Figure EV1. Characterization of an optimized NRF2 transcriptional signature.

(**A**) Comparison of expression features (red) predicting NRF2 dependency from the multi-omic feature analysis compared to NRF2 target genes identified in the literature (gray). Left - Proportional overlap of expression features with literature datasets. Number of genes identified in each literature set noted above the bars. Right - Proportion of genes that are identified in multiple literature sets that are also identified as top expression features. (**B**) Number of literature references for each NRF2 signature gene. (**C**) Western blot of shNRF2 or shNTC A549 and NCI-H460 cells $+/-400$ ng/mL dox. (**D**) Incucyte growth curves of shNTC or shNRF2 A549 or NCI-H460 cells with indicated dox concentrations. Validation data are from one experiment, with mean and SEM derived from 16 images/well indicated. (**E**) Volcano plots of RNA-seq values of NRF2 signature genes (pink) and compared to non-signature other genes (gray) in A549 and NCI-H460 cells shNRF2 cells treated with 500 ng/mL dox for indicated time points. Data are expressed relative to pre-treatment control Time points are relative to pre-treatment (0 h). Data are derived from two independent samples. (**F**) Distribution and density of gene counts across promoter NRF2 ChIP signal levels. The histogram represents the number of genes at each ChIP signal. The peak (dashed line) of the estimated probability density function (gray region) serves as the cut-off point for classification into group I (low promoter NRF2, green) and group II (high promoter NRF2, purple).

 © The Author(s)

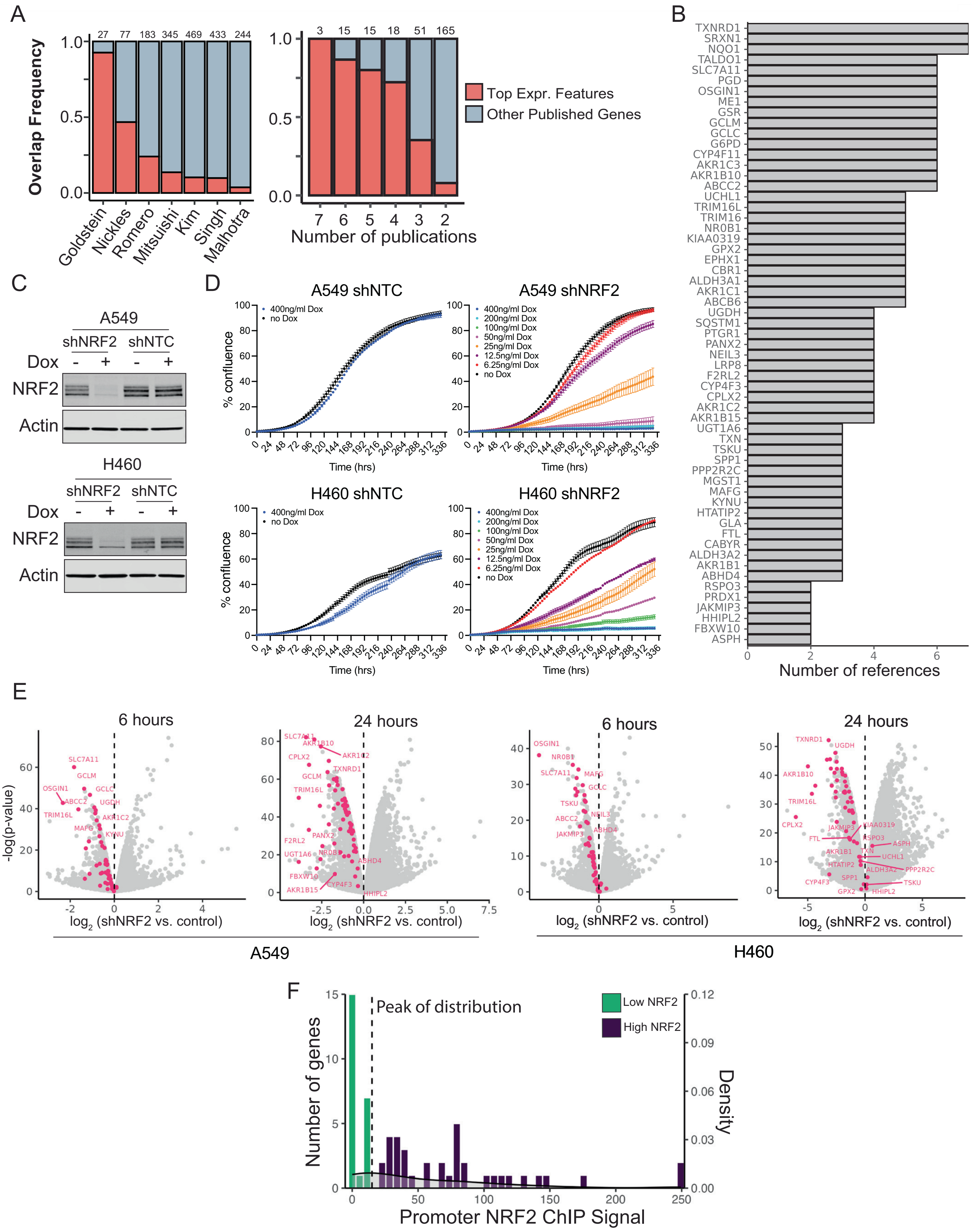
A
Overlap Frequency
1.0
0.5
0.0
27 77 183 345 469 433 244
Goldstein
Nickles
Romero
Mitsuishi
Kim
Singh
Malhotra
3 15 15 18 51 165
7 6 5 4 3 2
Number of publications
Top Expr. Features
Other Published Genes
B
TXNRD1
SRXN1
NQO1
TALDO1
SLC7A11
PGD
OSGIN1
ME1
GSR
GCLM
GCLC
G6PD
CYP4F11
AKR1C3
AKR1B10
ABCC2
UCHL1
TRIM16L
TRIM16
NR0B1
KIAA0319
GPX2
EPHX1
CBR1
ALDH3A1
AKR1C1
ABCB6
UGDH
SQSTM1
PTGR1
PANX2
NEIL3
LRP8
F2RL2
CYP4F3
CPLX2
AKR1C2
AKR1B15
UGT1A6
TXN
TSKU
SPP1
PPP2R2C
MGST1
MAFG
KYNU
HTATIP2
GLA
FTL
CABYR
ALDH3A2
AKR1B1
ABHD4
RSPO3
PRDX1
JAKMIP3
HHIPL2
FBXW10
ASPH
0 2 4 6
Number of references
C
A549
shNRF2 shNTC
Dox - + - +
NRF2
Actin
H460
shNRF2 shNTC
Dox - + - +
NRF2
Actin
D
A549 shNTC
A549 shNRF2
H460 shNTC
H460 shNRF2
400ng/ml Dox
200ng/ml Dox
100ng/ml Dox
50ng/ml Dox
25ng/ml Dox
12.5ng/ml Dox
6.25ng/ml Dox
no Dox
% confluence
Time (hrs)
E
6 hours
24 hours
-log(p-value)
log2 (shNRF2 vs. control)
A549
H460
F
Number of genes
Peak of distribution
Low NRF2
High NRF2
Density
Promoter NRF2 ChIP Signal

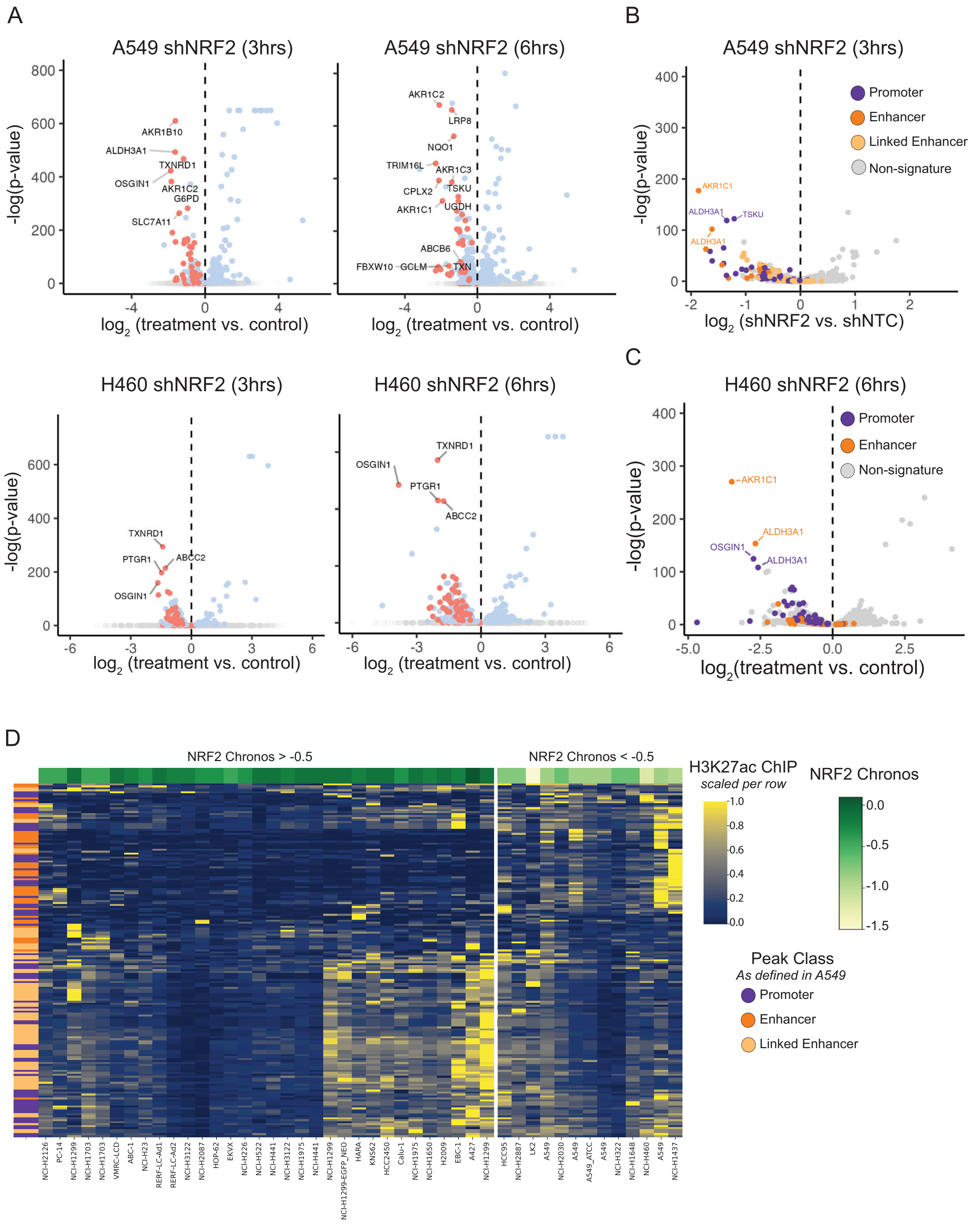
A
A549 shNRF2 (3hrs)
A549 shNRF2 (6hrs)
-log(p-value)
log2 (treatment vs. control)
AKR1B10
ALDH3A1
TXNRD1
OSGIN1
AKR1C2
G6PD
SLC7A11
AKR1C2
LRP8
NQO1
TRIM16L
AKR1C3
CPLX2
TSKU
AKR1C1
UGDH
ABCB6
FBXW10
GCLM
TXN
B
A549 shNRF2 (3hrs)
log2 (shNRF2 vs. shNTC)
Promoter
Enhancer
Linked Enhancer
Non-signature
AKR1C1
ALDH3A1
TSKU
H460 shNRF2 (3hrs)
H460 shNRF2 (6hrs)
TXNRD1
PTGR1
ABCC2
OSGIN1
C
H460 shNRF2 (6hrs)
log2(treatment vs. control)
D
NRF2 Chronos > -0.5
NRF2 Chronos < -0.5
H3K27ac ChIP
scaled per row
NRF2 Chronos
Peak Class
As defined in A549
NCI-H2126
PC-14
NCI-H1299
NCI-H1703
NCI-H1703
VMRC-LCD
ABC-1
NCI-H23
RERF-LC-Ad1
RERF-LC-Ad2
NCI-H3122
NCI-H2087
HOP-62
EKVX
NCI-H226
NCI-H522
NCI-H441
NCI-H3122
NCI-H1975
NCI-H441
NCI-H1299
NCI-H1299-EGFP_NEO
HARA
KNS62
HCC2450
Calu-1
NCI-H1975
NCI-H1650
H2009
EBC-1
A427
NCI-H1299
HCC95
NCI-H2887
LK2
A549
NCI-H2030
A549
A549_ATCC
A549
NCI-H322
NCI-H1648
NCI-H460
A549
NCI-H1437

Figure EV2. NRF2 is required for eRNA transcription and H3K27ac deposition.

(**A**) Volcano plot of the $\log_2$ fold change of the sum of all PRO-seq reads within a gene region. A549 and H460 shNRF2 treatment is 250 ng/mL dox at 3 and 6 h relative to control (water). Data are derived from three replicates for A549, and from two individual replicates for H460. (**B**) Volcano plot of individual (gene-agnostic) PRO-seq peaks in A549 shNRF2 at 3 h vs control (water). Data are derived from three replicates for A549, and from two individual replicates for H460. (**C**) Volcano plot of individual (gene-agnostic) PRO-seq peaks in H460 shNRF2 at 6 h vs control (water). Data are derived from three replicates for A549, and from two individual replicates for H460. (**D**) Heatmap of H3K27ac ChIP (fold change compared to input control) with 1 representing the highest value for every row. Rows represent genomic regions containing NRF2 responsive PRO-seq peaks and are colored by classification.

© The Author(s)

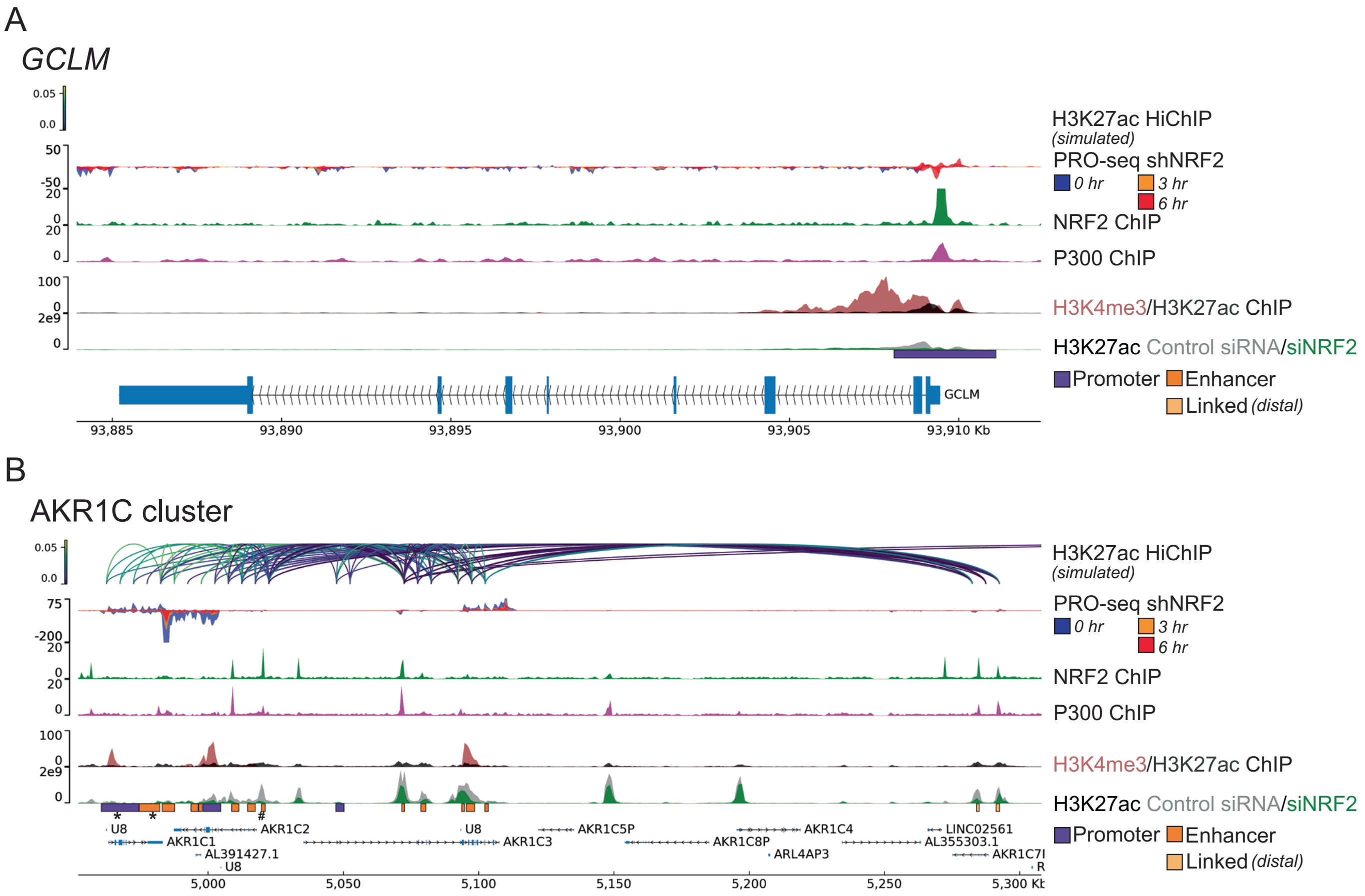

Figure EV3. Illustrative genome browser snapshots of group I and group II signature genes.

(**A**) Genome browser snapshots of the group II NRF2 signature gene GCLM highlighting the neighboring chromatin landscape. Simulated H3K27ac HiChIP links are colored by *p*-value. (**B**) Genome browser snapshots of the group I NRF2 signature genes AKR1C1-3 highlighting the highly linking chromatin landscape of this gene cluster. Simulated H3K27ac HiChIP links are colored by *p*-value. * denotes PRO-seq peaks from Fig. 2A. # denotes H3K27ac peak that is NRF2 responsive from Fig. 2D.

 © The Author(s)

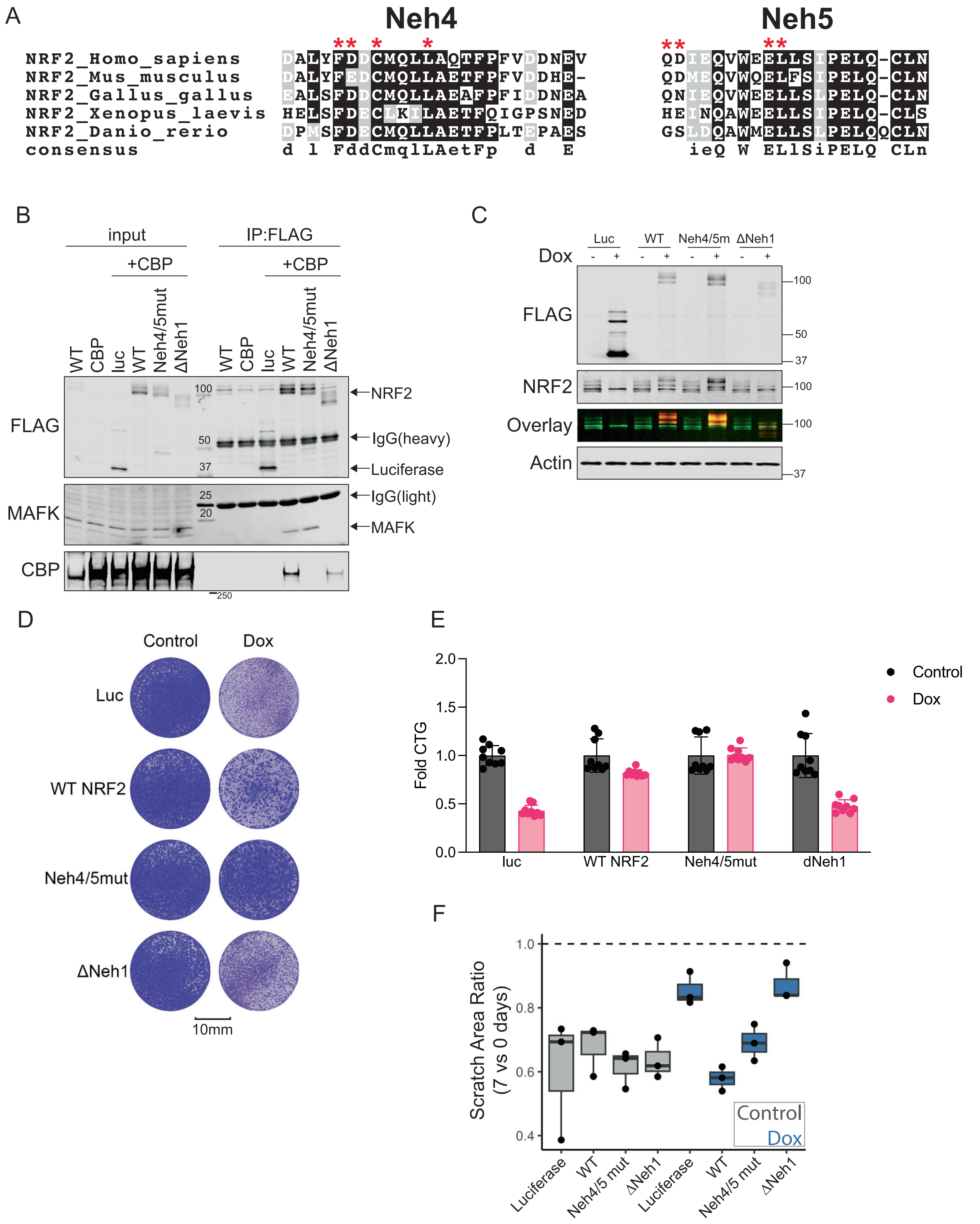
A
Neh4
Neh5
NRF2_Homo_sapiens DALYFDDCMQLLAQTFPFVDDNEV QDIEQVWEELLSIPELQ-CLN
NRF2_Mus_musculus DALYFEDCMQLLAETFPFVDDHE- QDMEQVWQELFSIPELQ-CLN
NRF2_Gallus_gallus EALSFDDCMQLLAEAFPFIDDNEA QNIEQVWEELLSLPELQ-CLN
NRF2_Xenopus_laevis HELSFDECLKILAETFQIGPSNED HEINQAWEELLSIPELQ-CLS
NRF2_Danio_rerio DPMSFDECMQLLAETFPLTEPAES GSLDQAWMELLSLPELQQCLN
consensus d l FddCmqlLAetFp d E ieQ W ELlSiPELQ CLn
B
input
IP:FLAG
+CBP
+CBP
WT
CBP
luc
WT
Neh4/5mut
ΔNeh1
FLAG
MAFK
CBP
100
50
37
25
20
250
NRF2
IgG(heavy)
Luciferase
IgG(light)
MAFK
C
Dox
Luc
WT
Neh4/5m
ΔNeh1
FLAG
NRF2
Overlay
Actin
100
50
37
D
Control
Dox
Luc
WT NRF2
Neh4/5mut
ΔNeh1
10mm
E
Fold CTG
2.0
1.5
1.0
0.5
0.0
luc
WT NRF2
Neh4/5mut
dNeh1
Control
Dox
F
Scratch Area Ratio
(7 vs 0 days)
1.0
0.8
0.6
0.4
Control
Dox
Luciferase
WT
Neh4/5 mut
ΔNeh1

Figure EV4. Definition and validation of a CBP/p300-deficient NRF2, Neh4/5mut.

(**A**) Alignment of metazoan NRF2 Neh4 and Neh5 segments with Neh4/5mut substitutions indicated. Mutation of QD in Neh5 was derived from (Zhang et al, 2007). (**B**) Western blot of input (left) and FLAG-immunoprecipitations (right) of FLAG-tagged WT, Neh4/5mut, ΔNeh1, or luciferase control expressing HEK293 cells co-transfected with or without GFP-tagged CBP. Data are representative of three independent experiments. (**C**) Western blotting of H460-shNRF2-BIND-luc, -BIND-WT NRF2, -BIND-Neh4/5mut, or -BIND-ΔNeh1 cells treated with water (control) or 2.5 ng/mL dox for 24 h probing with FLAG, NRF2, or β-actin antibodies. Representative data from three independent experiments is shown. (**D**) Crystal violet staining of H460-shNRF2-BIND-luc, -BIND-WT NRF2, -BIND-Neh4/5mut, or -BIND-ΔNeh1 treated with water (control) or 2.5 ng/mL dox for 8 days. Representative data from three independent experiments is shown. (**E**) CellTiter-Glo measurements of H460-shNRF2-BIND-luc, -BIND-WT NRF2, -BIND-Neh4/5mut, or -BIND-ΔNeh1 treated with water (control) or 2.5 ng/mL dox for 5 days. Data are expressed as a fold of control values, with SD indicated and are derived from three individual experiments. (**F**) Quantification of scratch assay wound healing in A549shNRF2-BIND cells +/−2.5 ng/mL dox imaged over 7 days on the Incucyte system. Wound healing rate is shown as the ratio of the scratch area at day 7 vs day 0, with a ratio of 1 representing no migration of cells into the scratch area. Data are derived from three individual replicates.

 © The Author(s)

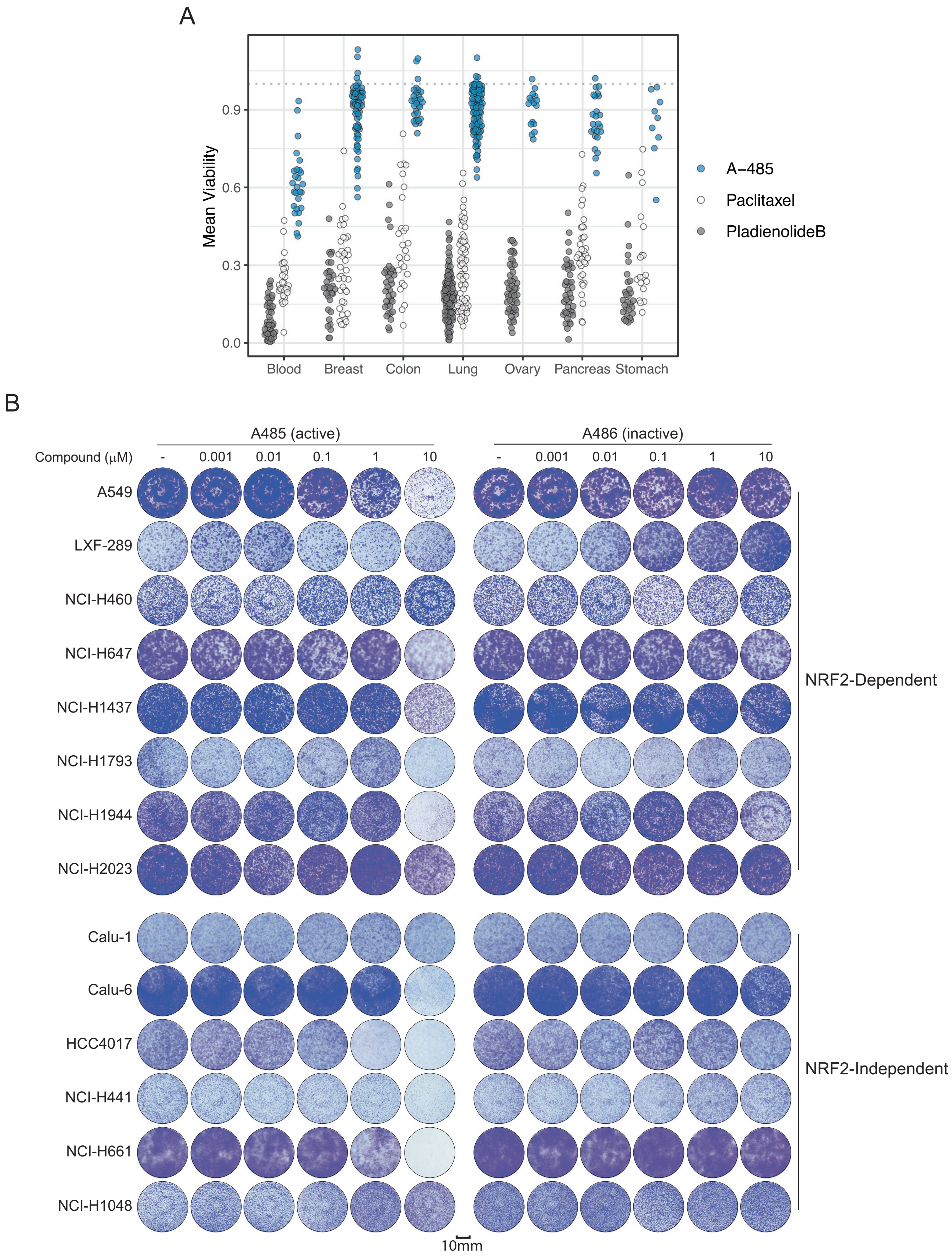
A
Mean Viability
0.0
0.3
0.6
0.9
Blood
Breast
Colon
Lung
Ovary
Pancreas
Stomach
A–485
Paclitaxel
PladienolideB
B
A485 (active)
A486 (inactive)
Compound (μM)
-
0.001
0.01
0.1
1
10
A549
LXF-289
NCI-H460
NCI-H647
NCI-H1437
NCI-H1793
NCI-H1944
NCI-H2023
Calu-1
Calu-6
HCC4017
NCI-H441
NCI-H661
NCI-H1048
NRF2-Dependent
NRF2-Independent
10mm

Figure EV5. Cell viability responses upon A-485 treatment across diverse cancer cell lines.

(**A**) Mean viability across 535 cell lines treated with a dose response of A-485 or the broadly active compounds (Paclitaxel or Pladienolide B) in 5-day viability assays determined by CellTiter-Glo. (**B**) Crystal violet staining of 14 cell lines, eight NRF2-dependent (Chronos < -0.5) and 6 NRF2-independent (Chronos > -0.5) treated with indicated concentrations of A-485 or A-486 control for 7 days. Data are representative of 2 independent experiments.

 © The Author(s)

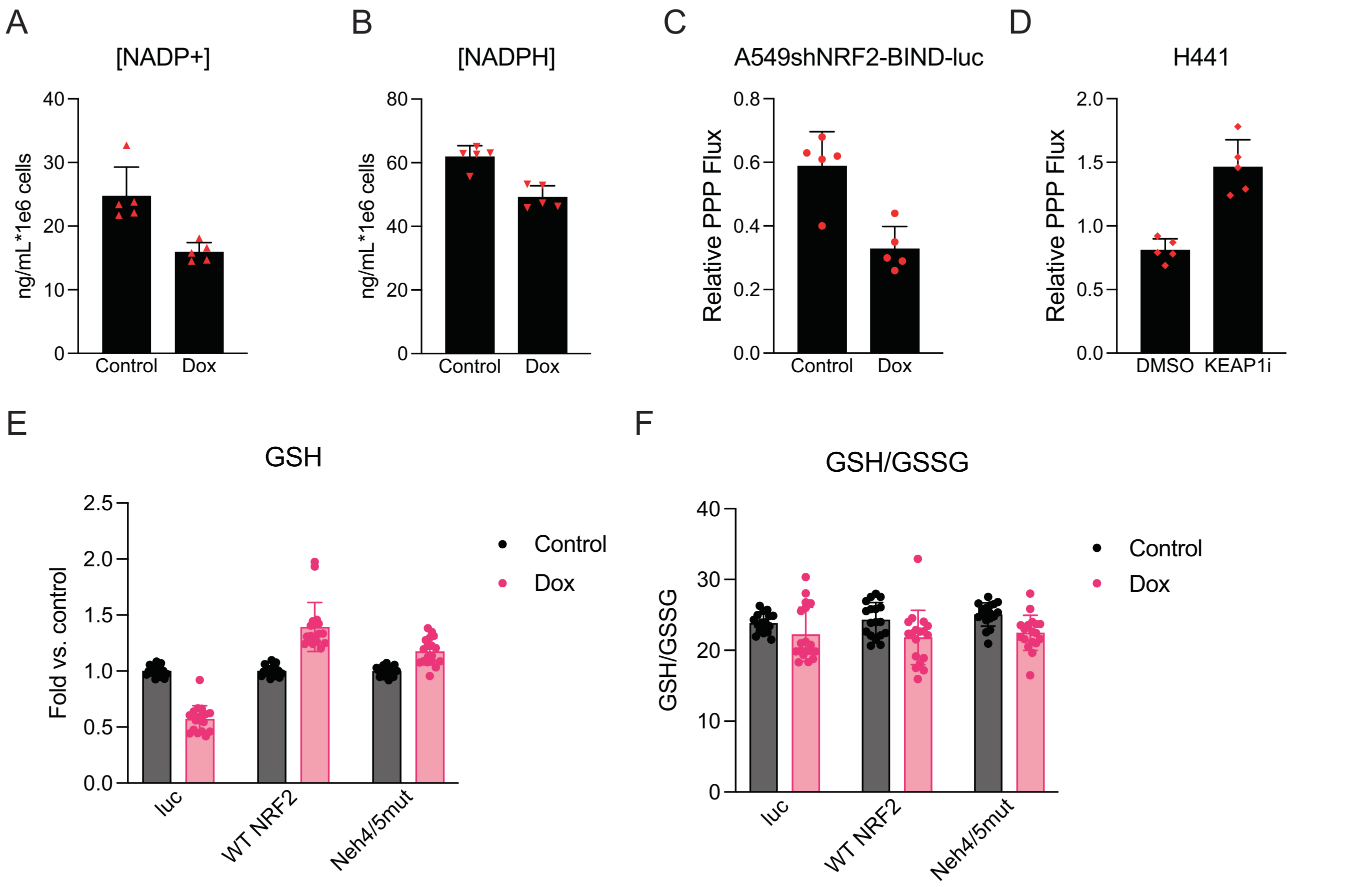

Figure EV6. Metabolic characterization of A549shNRF2-/H460shNRF2-BIND rescue cell models.

Mean NADP+ (**A**) and NADPH (**B**) levels in A549shNRF2-BIND-luc cells treated with water (Control) or 2.5 ng/mL doxycycline (Dox) for 48 h. Data are derived from 5 individual replicates with SD indicated. (**C**) Mean relative PPP Flux in A549shNRF2-BIND-luc cells treated with water (Control) or 2.5 ng/mL doxycycline (Dox) for 48 h and labeled with [1,2-^{13}C]-glucose for 14.5 h. Data are derived from 5 individual replicates with SD indicated. (**D**) Mean relative PPP Flux in H441 cells treated with DMSO control or 1 µM KEAPi (Davies et al, 2016) for 48 h and labeled with [1,2-^{13}C]-glucose for 14.5 h. Data are derived from 5 individual replicates with SD indicated. (**E**) Mean GSH levels in A549shNRF2-BIND-luc, -BIND-WT NRF2, -BIND-Neh4/5mut cells treated with water (control) or 2.5 ng/mL dox for 48 h. 2. Data represent three independent experiments with SD indicated. (**F**) Mean GSH/GSSG ratios in A549shNRF2-BIND-luc, -BIND-WT NRF2, -BIND-Neh4/5mut cells treated with water (control) or 2.5 ng/mL dox for 48 h. rescued with luciferase control, WT or Neh4/5mut NRF2. Data represent three independent experiments with SD indicated.

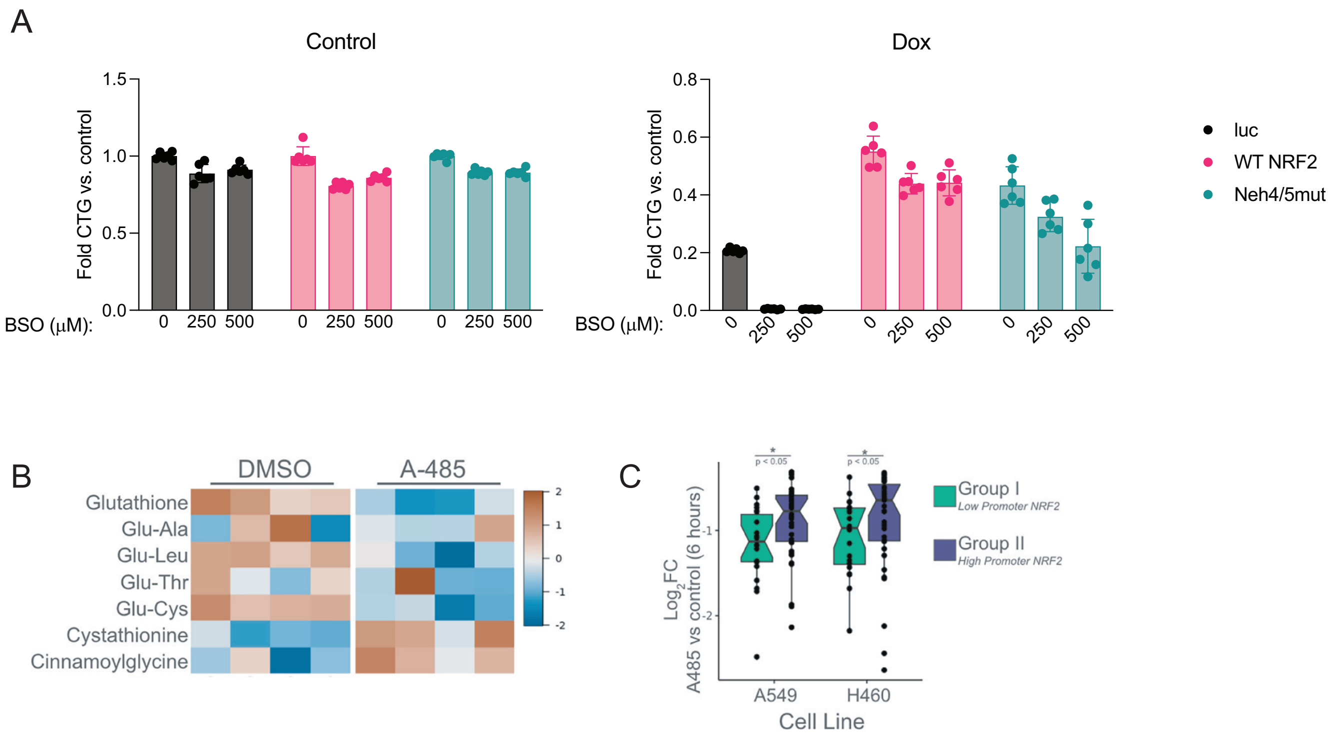

Figure EV7. BSO sensitivity in the A549shNRF2-BIND rescue system and mechanistic characterization of A-485 treatment in NRF2-dependent NSCLC models.

(**A**) Mean CellTiter-Glo measurements of A549shNRF2-BIND-luc, -BIND-WT NRF2, -BIND-Neh4/5mut cells treated with water (control) or 2.5 ng/mL dox for 48 h, then treated with indicated concentrations of BSO for 96 h. Data are expressed as a fold relative to parental control (no dox, no BSO), and are derived from two individual replicates performed in triplicate with SD indicated. (**B**) Heatmap of GSH pathway metabolites intensity normalized by row as a z score from A549shNRF2-BIND-luc cells treated with 1 μM A-485 for 24 h. Each column represents an individual replicate. (**C**) RNA-seq values of group I vs group II (Fig. 1B) genes from A549 and H460 cells treated with 1 μM A-485 for 6 h. Values show limma-voom $\log_2$ fold changes between A-485 treatment vs DMSO. Data are derived from three individual replicates. *P*-values derived from a Wilcoxon rank-sum test, followed by adjustment by the Benjamini–Hochberg procedure to correct for multiple testing. Box represents the first and third quartiles, with the median shown as a horizontal line. Whiskers extend to the smallest and largest value within 1.5 * the interquartile range. Notches extend 1.58 *IQR/sqrt(*n*). Group I $n = 23$, group II $n = 36$.

 © The Author(s)
